# Spatial grounding of symbolic arithmetic: an investigation with optokinetic stimulation

**DOI:** 10.1007/s00426-018-1053-0

**Published:** 2018-07-18

**Authors:** Elvio Blini, Marco Pitteri, Marco Zorzi

**Affiliations:** 1 ImpAct Team, Lyon Neuroscience Research Center, INSERM U1028, CNRS UMR5292, University of Lyon 1, Lyon, France; 2 Neurology section, Department of Neurosciences, Biomedicine and Movement Sciences, University of Verona, Verona, Italy; 3 Department of General Psychology and Padova Neuroscience Center, University of Padova, via Venezia 8, 35131 Padova, Italy; 4 IRCCS San Camillo Hospital Foundation, Lido-Venice, Italy

## Abstract

Growing evidence suggests that mental calculation might involve movements of attention along a spatial representation of numerical magnitude. Addition and subtraction on nonsymbolic numbers (numerosities) seem to induce a “momentum” effect, and have been linked to distinct patterns of neural activity in cortical regions subserving attention and eye movements. We investigated whether mental arithmetic on symbolic numbers, a cornerstone of abstract mathematical reasoning, can be affected by the manipulation of overt spatial attention induced by optokinetic stimulation (OKS). Participants performed additions or subtractions of auditory two-digit numbers during horizontal (experiment 1) or vertical OKS (experiment 2), and eye movements were concurrently recorded. In both experiments, the results of addition problems were underestimated, whereas results of subtractions were overestimated (a pattern that is opposite to the classic Operational Momentum effect). While this tendency was unaffected by OKS, vertical OKS modulated the occurrence of decade errors during subtractions (i.e., fewer during downward OKS and more frequent during upward OKS). Eye movements, on top of the classic effect induced by OKS, were affected by the type of operation during the calculation phase, with subtraction consistently leading to a downward shift of gaze position and addition leading to an upward shift. These results highlight the pervasive nature of spatial processing in mental arithmetic. Furthermore, the preeminent effect of vertical OKS is in line with the hypothesis that the vertical dimension of space–number associations is grounded in universal (physical) constraints and, thereby, more robust than situated and culture-dependent associations with the horizontal dimension.

**Electronic supplementary material:**

The online version of this article (10.1007/s00426-018-1053-0) contains supplementary material, which is available to authorized users.

## Introduction

The Spatial Numerical Association of Response Codes (SNARC) effect (Dehaene, Bossini, & Giraux, 1993) is a classic paradigm used in experimental psychology to probe number–space interactions. Typically, when asked to classify a number according to its magnitude (smaller vs. larger than a reference) or its parity (odd vs. even), participants are faster and more accurate when responding to smaller numbers in the left side of space, and to larger numbers in the right side of space (see Wood, Willmes, Nuerk, & Fischer, 2008, for a review). The hypothesis that number–space interactions tap an intrinsic property of the mental representation of numbers is still debated (e.g., Gevers, Verguts, Reynvoet, Caessens, & Fias, 2006; Gevers et al., 2010), even though supported by converging evidence from behavioural, neuropsychological (Zorzi et al., 2012), and neuroimaging studies (Cutini, Scarpa, Scatturin, Dell’Acqua, & Zorzi, 2012). It has been proposed that numerical magnitudes are mentally represented in a spatially ordered manner along a continuum, referred to as the Mental Number Line (MNL; Dehaene, 1992; Restle, 1970; Zorzi, Priftis, & Umiltà, 2002), and that number processing involves orienting of attention in this “number space” (Hubbard, Piazza, Pinel, & Dehaene, 2005; Umiltà, Priftis, & Zorzi, 2009; Zorzi et al., 2012; 2002). The link has been traced back to infancy in the form of spontaneous association between numerosity and spatial extent (de Hevia & Spelke, 2010; Lourenco & Longo, 2010). A spatial mapping of symbolic numbers emerges during the early preschool period and it appears to be crucial for understanding magnitude relationships for exact numbers (Sella, Berteletti, Lucangeli, & Zorzi, 2016). However, the specific spatial layout for the ordering of magnitudes is known to be strongly influenced by cultural habits such as reading/writing direction (Dehaene et al., 1993; Göbel, Shaki, & Fischer, 2011; Shaki, Fischer, & Petrusic, 2009). According to a recent theoretical framework (Fischer, 2012; Myachykov, Scheepers, Fischer, & Kessler, 2014), at the origins of this spatial arrangement may reside “situated” aspects of cognition, reflecting flexible representations rapidly changing as a function of task demands, experimental context, or available resources (e.g., Bächtold, Baumüller, & Brugger, 1998; Fischer, Mills, & Shaki, 2010; Pfister, Schroeder, & Kunde, 2013; Vuilleumier, Ortigue, & Brugger, 2004). In contrast, a more deeply rooted aspect of numerical cognition, reflecting “grounded” aspects and hence physical invariants of the surrounding environment (Fischer, 2012; sometimes referred to “tropisms”, Myachykov et al., 2014), is the universal tendency to associate small magnitudes with lower space and large magnitudes with upper space (Ito & Hatta, 2004; Shaki & Fischer, 2012). This implies that vertical spatial–numerical associations should be more robust than horizontal ones, a prediction which has received some initial support (Fischer & Brugger, 2011; Wiemers, Bekkering, & Lindemann, 2014). Note that this discussion is cast into the broader theoretical framework of embodied cognition, which opposes the traditional view of cognition as abstract symbol manipulation (Barsalou, 2008) and inspired the notion of embodied number processing (Fischer, 2012; Fischer & Brugger, 2011; Lakoff & Núñez, 2000; Marghetis, Núñez, & Bergen, 2014).

An intriguing extension to the literature on number–space interactions comes from the study of spatial biases in mental arithmetic (Fischer & Shaki, 2014, for review). The Operational Momentum (OM) effect, first documented by McCrink et al., (2007), refers to the tendency to overestimate the result of additions and underestimate the result of subtractions. The authors suggested that the OM might be ascribed to attentional movements along an internal (mental) numerical continuum, thereby mirroring other forms of perceptual and representational momentum (see Hubbard, 2014, for a review; for an overview of different accounts see Knops, Zitzmann, & McCrink, 2013; McCrink & Wynn, 2009). The OM has been replicated in a variety of experimental conditions, although most typically when the task involves nonsymbolic operands (e.g., Knops, Dehaene, Berteletti, & Zorzi, 2014; Knops, Viarouge, & Dehaene, 2009; McCrink et al., 2007; McCrink & Wynn, 2009). The OM has been also observed using a variety of response modalities, such as multiple choice (e.g., Knops et al., 2014; McCrink et al., 2007), pointing to a location along a visual segment (Klein, Huber, Nuerk, & Moeller, 2014; Pinhas & Fischer, 2008), and dot production (Lindemann & Tira, 2011). Notably, Pinhas, Shaki, and Fischer (2014) observed spatial associations even for the operation symbol: the addition operator (plus) was associated to faster right-sided responses, whereas the subtraction operator (minus) was associated to faster left-sided responses. Neuroimaging evidence suggests that cortical structures devoted to attention/eye movements are recruited during mental arithmetic (Knops, Thirion, Hubbard, Michel, & Dehaene, 2009). Knops et al. (2009) observed that the pattern of brain activity in parietal cortex related to right-sided eye movements was also present during addition. Finally, addition and subtraction have been shown to produce spatial biases (leftwards and rightward, respectively) on attention orienting (Masson & Pesenti, 2014; Mathieu, Gourjon, Couderc, Thevenot, & Prado, 2016) and oculomotor behaviour (Hartmann, Mast, & Fischer, 2015; Holmes, Ayzenberg, & Lourenco, 2016; Werner & Raab, 2014). Note that the latter effects are in line with those triggered by isolated digits (e.g., Blini, Cattaneo, & Vallar, 2013; Casarotti, Michielin, Zorzi, & Umiltà, 2007; Fischer, Castel, Dodd, & Pratt, 2003; Klein et al., 2014; but see Fattorini, Pinto, Rotondaro, & Doricchi, 2015; Zanolie & Pecher, 2014).

The hypothesis that mental arithmetic might be rooted in sensorimotor mechanisms is very intriguing, but it remains unclear which specific processes might be affected and to what extent. In this regard, an important contribution towards a better understanding of the spatial underpinnings of mental arithmetic comes from neuropsychological studies of patients with Unilateral Spatial Neglect (USN), a disorder consisting in the failure to report, respond to or orient towards stimuli in the contralesional side of space (more commonly the left one, Heilman, Watson, & Valenstein, 1985; Vallar, 1998). The initial finding that USN patients show deficits in number processing that are readily interpreted in terms of neglect for the number space (Zorzi et al., 2002; see Umiltà, Priftis, & Zorzi, 2009, for a review) has been followed-up by many studies (e.g. Bonato, Priftis, Marenzi, & Zorzi, 2008; Priftis et al., 2008; Priftis, Zorzi, Meneghello, Marenzi, & Umiltà, 2006; Rossetti et al., 2004; van Dijck, Gevers, Lafosse, Doricchi, & Fias, 2011; Vuilleumier, Ortigue, & Brugger, 2004; Zorzi et al., 2012; Zorzi, Priftis, Meneghello, Marenzi, & Umiltà, 2006). The recent extension to mental arithmetic has revealed that USN patients present an increased error rate when performing subtractions with respect to additions (Benavides-Varela et al., 2014; Dormal, Schuller, Nihoul, Pesenti, & Andres, 2014). Dormal et al. (2014) observed that performance in subtractions was particularly affected when the second operand was large (and a borrowing operation was involved); the authors thus suggested that patients could present difficulties in accessing numerical representations that are located to the left (contralesional) side of the first operand along a continuum transiently built in working memory. A recent single-case report also described a patient with right-sided neglect who showed a specific impairment for additions (Masson, Pesenti, Coyette, Andres, & Dormal, 2017). This is in line with several neuropsychological studies that—starting with the seminal study by Hécaen, Angelergues, & Houillier (1961), who introduced the term “spatial acalculia”—describe calculation deficits that are secondary to visuospatial ones (Ardila & Rosselli, 1994; Boller & Grafman, 1983; de Hevia, Vallar, & Girelli, 2008, for review). Typical errors of these patients include the incorrect alignment of numbers in column, difficulties in maintaining the decimal places, and so on, thus being at least partly dissociable from non-strategic, approximation-related errors (Benavides-Varela et al., 2016; de Hevia et al., 2008).

The crucial test for the hypothesis that attentional movements are functionally involved in mental arithmetic is to assess whether the latter can be affected by explicit experimental manipulations of visuospatial attention in healthy participants (in contrast to “nature’s experiments” based on patients with brain damage). Notably, the embodied cognition framework predicts that conceptual processing should be influenced by the sensorimotor mechanisms they rely on (Barsalou, 1999; Lakoff & Johnson, 1999) and that the influences between semantic and sensorimotor processing should be bi-directional (Gentilucci & Gangitano, 1998). For example, Stoianov, Kramer, Umiltà, and Zorzi (2008) observed that lateralized, irrelevant spatial cues can modulate symbolic number comparison performance when they temporally overlap with the processing of numerical stimuli (also see Kramer, Stoianov, Umiltà, & Zorzi, 2011). Masson and Pesenti (2016) recently extended this observation to mental arithmetic by showing that lateralized distracters interfered with calculation in a side-specific manner (i.e., left distracters affected performance in subtraction, whereas right distracters affected additions).

Another way to explicitly manipulate spatial attention is to exploit optokinetic stimulation (OKS) (Pizzamiglio, Frasca, Guariglia, Incoccia, & Antonucci, 1990) and thus eye movements (e.g., Casarotti, Lisi, Umiltà, & Zorzi, 2012; Moore, Armstrong, & Fallah, 2003). OKS consists of full-field visual stimuli (e.g., vertical stripes) moving coherently towards one specific direction. Such stimulation imposes eye movements by means of a peculiar physiological reflex (the optokinetic nystagmus) consisting in two alternating phases: first, the eyes start following the movement of the stimulation (pursuit phase); second, after a variable amount of time a compensatory saccade is made in the opposite direction, allowing the return to the initial position. The mean position of the eyes during OKS is usually shifted towards the side of saccadic eye movements (beating field), possibly as a compensatory attempt to re-orient the eyes in the direction which is implied by the optic flow (Watanabe, 2001). The direction of slow, pursuit eye movements, on the other hand, triggers spatial attention shifts (e.g., leftward for leftward coherent motion) that have been widely exploited for neglect rehabilitation (e.g., Kerkhoff, Keller, Ritter, & Marquardt, 2006). When applied to USN patients, alongside with other rehabilitation techniques, number processing has been found to improve together with visuospatial processing (Priftis, Pitteri, Meneghello, Umiltà, & Zorzi, 2012; Rossetti et al., 2004; Salillas, Granà, Juncadella, Rico, & Semenza, 2009; Vuilleumier et al., 2004; but see Pitteri et al., 2015). Notably, the OKS technique is also effective in modulating number processing in healthy participants. Ranzini et al. (2015) found that rightward OKS during number comparison abolished the typical response time disadvantage for large numbers compared to smaller numbers. Ranzini, Lisi, & Zorzi (2016) found that eye movements (both saccades and pursuit) modulated comparison response times for large numbers as a function of motion direction. In the unique previous study that investigated the effect of horizontal OKS on arithmetic performance, Masson, Pesenti, and Dormal (2016) found that rightward OKS speeded addition problems, but only those involving carry operations. OKS did not influence subtraction problems. Moreover, there was no effect of OKS on errors, possibly because the error rate was low (about 6% across conditions) and it was not broken down into procedural vs. estimation-related errors. In contrast, in the present study we investigated whether the strong manipulation of eye movements and spatial attention provided by OKS may affect the distribution of error responses to challenging mental arithmetic problems (i.e., addition and subtraction of pairs of two-digit numbers).

To this aim, we examined the distribution of error responses to assess the prediction that leftward OKS would induce underestimation of the correct result, whereas rightward OKS would induce overestimation when compared to a neutral condition (i.e., static OKS). In the mental addition and subtraction of two-digits numbers, two main procedures have been documented (Beishuizen, Van Putten, & Van Mulken, 1997; Fuson, 1992). The “base-ten” procedure is based on decomposition of each operand into tens and ones, which are then separately processed and recombined only in the last step. The “sequential” procedure starts instead with counting by tens up or down from the first operand. As mental operations with the tens represent a crucial aspect in both procedures, we operationally considered decade errors (i.e., errors differing from the correct answers by a multiple of ten units) as a signature of procedural errors, which are known to be particularly affected by spatial deficits (Dormal et al., 2014). Furthermore, we considered all remaining errors (i.e., in the unit range) as index of estimation errors, and also predicted a possible modulation by OKS. Concerning the direction of this modulation, both (voluntary) saccadic and pursuit eye movements have been found to impact number processing (Ranzini et al., 2016). However, based on previous studies with OKS (Masson et al., 2016; Ranzini et al., 2015), we predicted shifts in the number space coherent with the OKS direction (i.e., in the direction of pursuit eye movements, and away from the beating field). Specifically, in keeping with the “moving along the mental number line” analogy (McCrink et al., 2007), we hypothesized rightward OKS to induce overestimation of the correct result (i.e., more pronounced rightward movement along the line) and leftward OKS to induce underestimation, similarly for additions and subtractions and on top of the OM effect. Finally, we hypothesized that eye movements would be spatially biased as a function of the type of operation (addition vs. subtraction) (Hartmann, Mast, & Fischer, 2015; Klein et al., 2014; Pinhas & Fischer, 2008), independently of OKS conditions. In particular, we predicted a shift in the mean position of the eyes (leftward for subtractions, rightward for additions), but only during the calculation phase (Liu, Cai, Verguts, & Chen, 2017; Masson, Letesson, & Pesenti, 2017).

To the best of our knowledge, no previous study has simultaneously probed the existence of bi-directional links between mental calculation and eye movements. Additionally, in the present study we sought to investigate the impact of a vertical, beside the more canonical horizontal, direction of OKS. Vertical OKS has never been used in previous studies on number processing or calculation, but assessing its effects is of primary theoretical significance in light of the hypothesis that changes in magnitude along the vertical axis (where “up” represents “more”) are more directly linked to human sensorimotor experience, as in the actions of stacking or removing objects from a pile (Fischer & Brugger, 2011). Vertical OKS might be more prone to affect participants’ performance than horizontal OKS because the former refers to a grounded aspect (as opposed to a situated) of numerical cognition (Fischer, 2012; Myachykov et al., 2014). With respect to the predictions, we expected upward OKS to induce overestimation of the correct result (similarly to rightward OKS), and downward OKS to induce underestimation of the correct result (similarly to leftward OKS).

To summarize, we sought to concurrently assess: (1) the effect of overt shifts of attention/eye movements on mental calculation, as well as the reciprocal effect (i.e., the effect of solving arithmetic operations on oculomotor control); (2) differential effects of horizontal vs. vertical OKS; (3) differential effects of the spatial manipulation in terms of types of error.

## Experiment 1

The first experiment assessed the effect of horizontal OKS on mental calculation. Participants performed both additions and subtractions with auditorily presented two-digit numbers.

### Participants

Twenty-four students recruited at the University of Padua, native Italian speakers, with no history of neurological disorders, and normal or corrected to normal vision, were enrolled in this study. Participants were not given explanations about the rationale of the study until the debriefing, at the end of the experiment. Sample size was similar to a previous study exploring the effects of OKS in mental arithmetic (*N* = 21, with one drop-out; Masson et al., 2016). All participants joined voluntarily and gave written informed consent prior to participate. The study followed the Declaration of Helsinki standards and was approved by the Ethical Committee of the University of Padua. The sample was composed of 10 males (41%), mean age was 21.71 years (range 20–27, SD 1.57), mean years of formal education were 13.64 (range 13–16, SD 1.25).

### Apparatus and stimuli

Eye movements were monitored online and recorded at 60 Hz with a Tobii T120 screen-based eye-tracker (Tobii Technology, Sweden), which was also used to present OKS (moving bars) through its embedded 17-inch TFT monitor. The sampling rate of 60 Hz is not optimal for the study of saccadic eye movements, but it is appropriate for our aim of assessing changes in mean eye position as a function of experimental conditions (see below). E-Prime 2.0 software (Psychology Software Tools, Pittsburgh, PA) was used to run the arithmetic tasks. OKS consisted of white vertical stripes (width: ~ 1.4°, height: ~ 25°, inter-stripe distance: ~ 1.4°) presented against a black background and moving leftward or rightward on the horizontal plane at a constant speed of 8.4 cm/s (~ 12°/s).

Each participant solved 60 arithmetic problems for each of the three OKS conditions (plus 6 practice items taken from a different set of stimuli at the beginning, 6 + 60 + 60 + 60 = 186 items overall). Within each block, participants performed 30 additions and 30 subtractions in random order; all problems’ results were large (> 20) to induce higher rates of calculation errors. Subtractions were mostly obtained by inverting additions (e.g., 27 + 29 = 56, 56–29 = 27) to balance the overall magnitude of the problem (as in Wiemers, Bekkering, & Lindemann, 2014), although we used some flexibility to avoid results deemed too easy (e.g., multiple than 10). As a result, the mean correct result was higher for addition than for subtraction (83.5 and 39.9, respectively). In addition, also note that the average magnitude of the first operand was larger for subtractions. Stimuli are reported in Table 2 in Appendix 1. Participants performed the same operations, with the same numbers, in each OKS condition, to allow for the comparison of the performance across OKS conditions between items with identical difficulty.

### Procedure

**Fig. 1:**
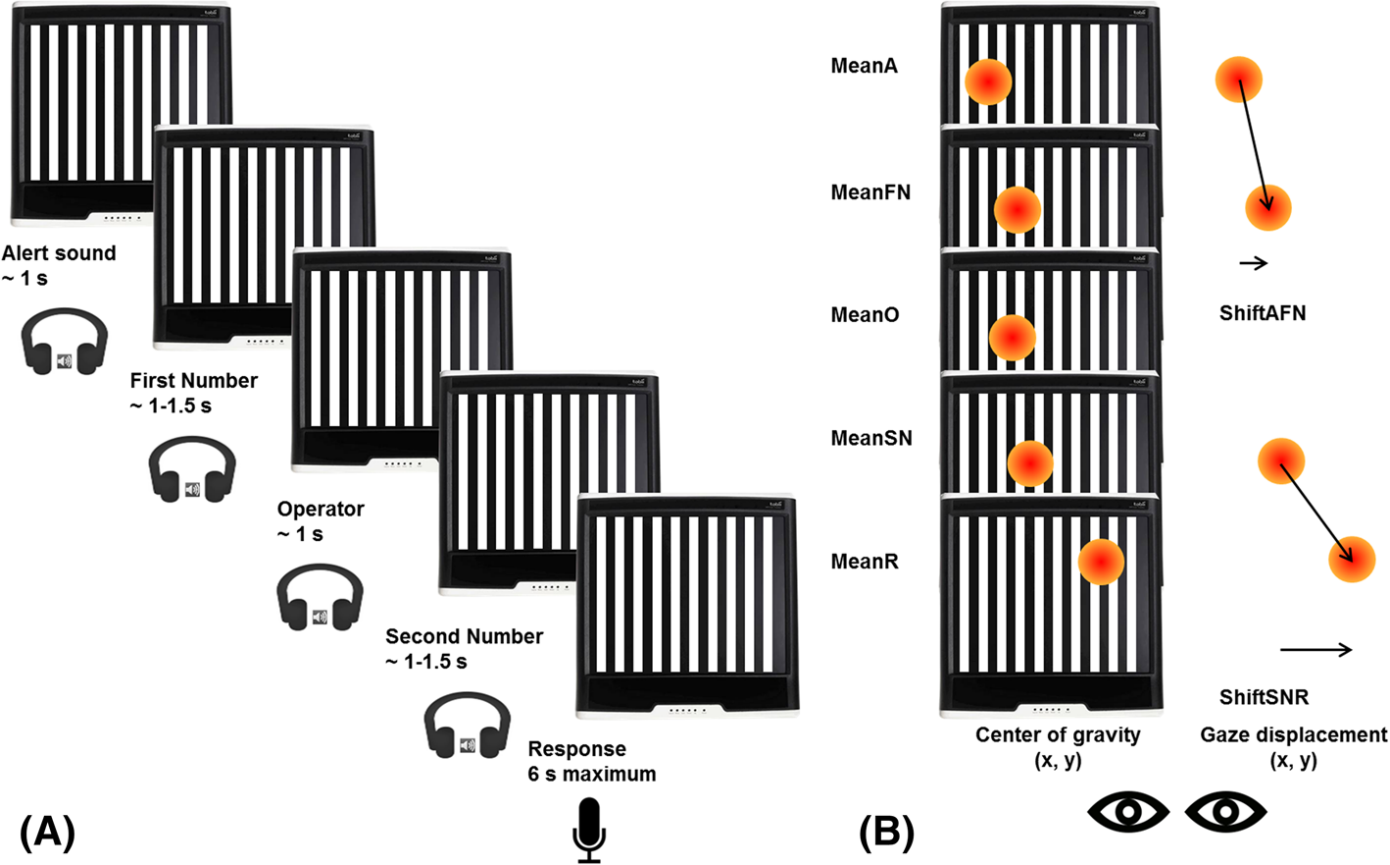
A graphical depiction and time course of a typical trial is depicted in panel **a**. Panel **b** depicts schematically the eye-tracking indices we used, namely the center of gravity of gaze during each phase of the trial and the displacement occurring between different phases, both on the horizontal and vertical axes

The experiment was carried out in a quiet and dimly lit room. The participant was sitting in front of the screen at a distance of approximately 40 cm. At the beginning of each OKS moving block, the experimenter first presented the moving bars and ensured that the participant’s optokinetic nystagmus was triggered before the beginning of the task. The task consisted in performing arithmetical operations (additions and subtractions) while OKS was visually presented. Stimuli were presented acoustically via stereo headphones, in the following order (depicted in Fig. 1, panel a): (1) an alert sound (“beep”), preceding the three elements of the operation; (2) a first numerical operand; (3) an operator (“plus” or “minus”); (4) a second numerical operand. Stimuli were obtained from a vocal synthesizer. Responses were given vocally, and latencies were collected via a microphone that triggered a voice key. Participants were asked to be as accurate as possible, but also fast; they were strongly encouraged to provide an estimate if one operation took too long, and, in any case, to provide a response before proceeding with the next trial. All the elements were presented to the participants while observing OKS in three conditions: static, leftward, and rightward. The static OKS (control condition) was always performed as first or last (counterbalanced across participants); the order of moving OKS conditions (left- or rightward) was also counterbalanced across participants. Within each block, stimuli were randomly presented to the participants, and three breaks (one every 20 items) were provided as to allow the participant to rest their eyes. The whole experiment lasted from 50 to 60 min.

The duration of the alert sound and of the two operators was of about 1 s; numbers lasted from about 1 s to about 1.5 s, depending on the length of the number word (note, however, that even if within the same block different numbers require different presentation times we are comparing the same stimuli across each OKS condition). Starting from the presentation of the second number, participants had 6 s to provide an answer. When a response was not provided within the time limit, the experimenter urged for an estimated response (after the practice phase this occurred in less than 1% of the trials). The response deadline was introduced to discourage the use of complex arithmetic procedures as well as to increase error probability, in line with our aim to focus on response distribution.

### Behavioural data

Participants generally complied with the instructions by providing a response within the time limit of 6 s in the large majority of cases during experimental trials (> 99%). We excluded from analysis trials with deviant eye movements (that may indicate difficulties in maintaining the nystagmus or lapse of attention). Trials with deviant eye movements (3.16%) were automatically identified by the eye tracker and later discarded during offline analysis. Horizontal eye-wandering was tolerated along the full length of the screen, while vertical eye-wandering had to be limited to an area covering 2/3 of the vertical screen, and centered in the middle of the vertical axis. More than two samples in a row outside this confidence area resulted in the eye tracker reporting eye movements as deviant. This procedure ensured that optokinetic nystagmus was effectively triggered. Few trials in which participants were not able to respond (even when urged to do so) were also discarded (0.19%).

**Fig. 2:**
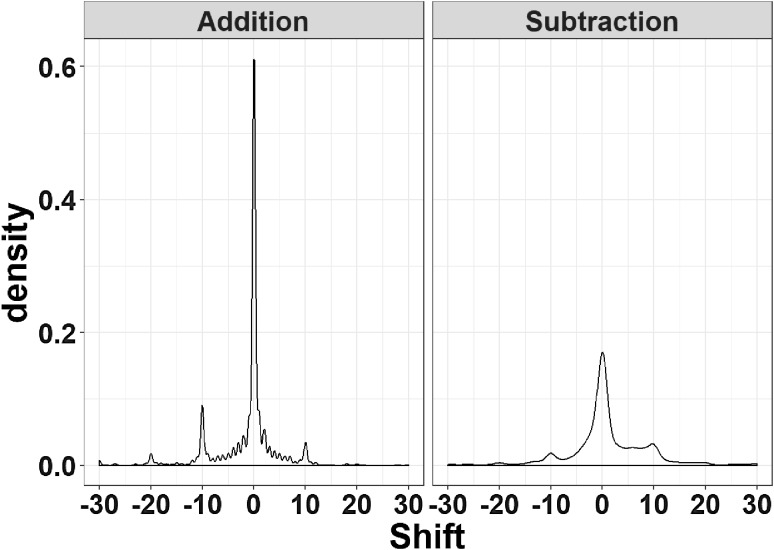
The distribution of responses is depicted as a function of Operation Type, collapsed across OKS conditions and participants. A value of 0 for Shift corresponds to a correct response, whereas negative and positive values reflect under- or overestimation of the correct result, respectively. Additions (right panel) were more accurate than subtractions (left panel), but in both cases the distribution is multimodal, mainly because of decade errors. Negative decade errors occurred more often in additions, whereas in subtractions participants committed more frequently positive decade errors

The distribution of responses was computed in terms of deviation from the correct arithmetic result (correct response minus participant response). The distribution, depicted in Fig. 2, is multimodal and characterized by multiple relative peaks. Indeed, though the majority of responses are distributed around the correct response, with relatively small deviations in the units range, there are other peaks in correspondence of large deviations of a multiple of 10 units on both sides of the correct result. We therefore considered the latter as “decade errors”, either positive or negative, and computed their proportion as an index of procedural errors during calculation. Conversely, the deviation from the correct result after excluding all decade errors reflects under- or overestimation in the units range and it was used as an index of estimation errors.

#### Description of indices and analyses

##### Deviation

The mean deviation from the correct result was used to assess the presence of OM. Trials in which decade errors were made (10.95% of the valid trials, see below) were discarded to isolate estimation errors from procedural ones. Trials in which a correct response was provided (Shift equal to 0) were included in the analyses.^1^ The mean values of Shift for each OKS (left, right, static) and Operation Type (addition, subtraction) conditions were then submitted to a 3 × 2 repeated measures ANOVA. A Bayesian counterpart for the ANOVA was also performed (Rouder, Morey, Speckman, & Province, 2012) to complement the analyses with an index that quantifies the strength of evidence (i.e., the Bayes Factor, BF; Kass & Raftery, 2012). A Bayesian analysis also allows one to assess the evidence for the null hypothesis, whereas a frequentist one is unable to do so (Kass & Raftery, 2012). BFs favour the alternative hypothesis if larger than 1, the null hypothesis if smaller; they are regarded as not conclusive if fall in the 0.33–3 interval. Post hoc analyses were carried using both Bonferroni corrected and Bayesian *t* tests (Rouder, Speckman, Sun, Morey, & Iverson, 2009) through the BayesFactor package for R (Morey, Rouder, & Jamil, 2015). All Bayesian tests exploited objective Cauchy distributed priors (i.e., assuming that 50% of observed normalized effect sizes might fall in the − 0.7 to + 0.7 interval).

##### Decade errors

The proportion of procedural errors (namely, where the observed Shift value was a multiple of 10, either positive or negative) was computed with respect to the overall number of trials. The mean values for each OKS × Operation Type conditions were then arcsine transformed—as the distributions of proportions were skewed—and submitted to a 3 × 2 repeated measures ANOVA.

#### Results

##### Accuracy and RTs

Analyses of accuracy and response times (RTs) only showed an advantage for additions over subtractions for the former (main effect of Operation type: *F*(1,23) = 51.7, *p* < 0.001, *η*_p_^2^ = 0.69); there were no other effects due to OKS or its interaction with Operation type (all *ps* > 0.17). Graphical depictions can be retrieved in the supplementary materials (Figure **S1** for accuracy, Figure **S2** for RTs).

##### Deviation

**Fig. 3:**
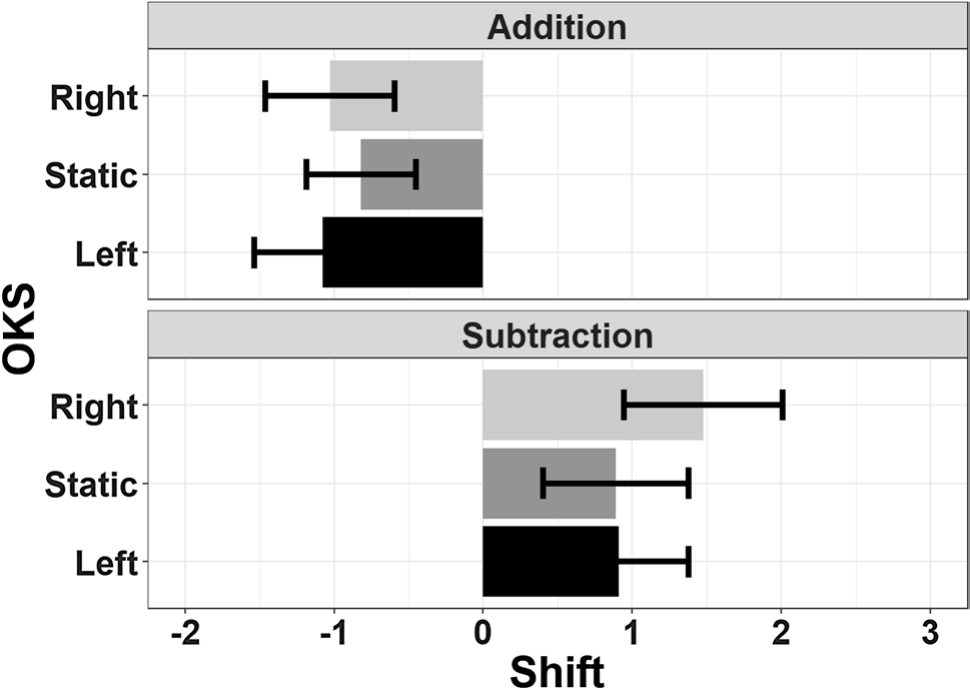
The mean estimation error is depicted as a function of Operation Type and OKS. Note that the OM effect was reversed, with underestimation for additions and overestimation for subtractions. No effects of horizontal OKS were found. Error bars represent within-subjects SEM (Morey, 2008)

The main effect of Operation Type was the only significant effect (*F*(1,23) = 9.03, *p* = 0.006, *η*_p_^2^ = 0.282, BF = 3.3_10_^5^); additions were overall linked to underestimation, while subtractions were more likely to induce overestimations, which is a pattern with the opposite direction with respect to the classic OM. Neither the main effect of OKS (*F*(2,46) = 0.57, *p* = 0.57, *η*_p_^2^ = 0.024, BF = 0.08) nor the Operation Type by OKS interaction (*F*(2,46) = 0.89, *p* = 0.42, *η*_p_^2^ = 0.037, BF = 0.15) were significant (Fig. 3).

##### Decade errors

**Fig. 4:**
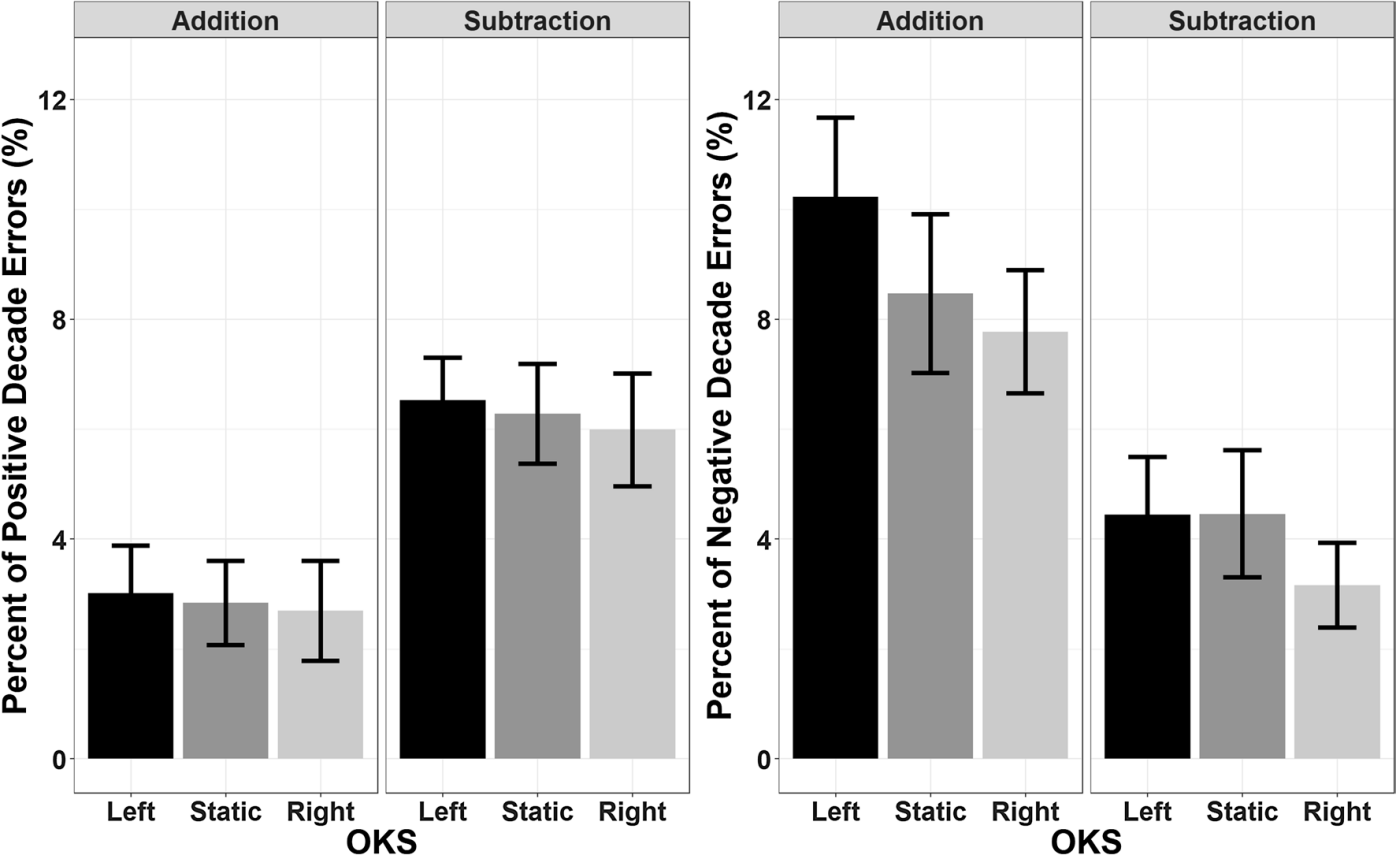
The percentage of decade errors (i.e., responses deviating from the correct result by a multiple of 10 units) across all trials is depicted as a function of Operation Type and OKS; the left panel reports negative decade errors, whereas the right panel reports positive decade errors. No modulatory effect of horizontal OKS was found. Error bars represent within-subjects SEM (Morey, 2008)

The main effect of Operation Type (*F*(1,23) = 11.96, *p* = 0.002, *η*_p_^2^ = 0.342, BF = 1735) yielded significance when analysing negative decade errors, with no main effect of OKS (*F*(2,46) = 1.93, *p* = 0.16, *η*_p_^2^ = 0.08, BF = 0.2) or interactions (*F*(2,46) = 0.14, *p* = 0.87, *η*_p_^2^ = 0.006, BF = 0.12). Specifically, negative decade errors were more frequent (8.8% of all responses) during addition than subtraction (4%). A similar pattern was obtained for positive decade errors, in which Operation Type was found to be significant (*F*(1,23) = 8.56, *p* = 0.008, *η*_p_^2^ = 0.271, BF = 1440) and an effect of OKS was not highlighted, neither alone (*F*(2,46) = 1.42, *p* = 0.25, *η*_p_^2^ = 0.058, BF = 0.15) nor in interaction (*F*(2,46) = 0.04, *p* = 0.97, *η*_p_^2^ = 0.002, BF = 0.12) with Operation Type. Subtractions were in this case more likely to induce positive decade errors (6.2%) than additions (2.8%, see Fig. 4).

### Eye-tracking data

We analysed, as dependent variables, both the mean position of the eyes along the horizontal or vertical axis of the screen (MeanFN and MeanR for the mean center of gravity during the presentation of the First Number and Response phases, respectively) and the shift in the mean eye position occurring between different phases of the trial (ShiftAFN, from Alert to the First Number presentation, and ShiftSNR, from the presentation of the Second Number to the Response stage; Fig. 1, panel B, and Table 1).

**Table 1:** Summary and description of eye-tracking indices

Index	Description	Interpretation
MeanFN	The mean center of gravity of eye position during the presentation of the first number	Low values indicate relatively leftward/downward spatial exploration High values indicate relatively rightward/upward spatial exploration
MeanR	The mean center of gravity of eye position during the response phase	Low values indicate relatively leftward/downward spatial exploration High values indicate relatively rightward/upward spatial exploration
ShiftAFN	The displacement of mean eye position occurring from the alert sound to the presentation of the first number	Negative values indicate a leftward/downward displacement of spatial attention Positive values indicate a rightward/upward displacement of spatial attention
ShiftSNR	The displacement of mean eye position occurring from the presentation of the second number to the response phase	Negative values indicate a leftward/downward displacement of spatial attention Positive values indicate a rightward/upward displacement of spatial attention

#### Description of indices and analyses

Deviant eye movements were first excluded from analysis (3.16%). The following dependent variables were computed: (1) the mean center of gravity of eye position (i.e., mean position both on the horizontal and vertical axis) during the response phase (MeanR); (2) the mean shift of the eyes from the presentation of the second number to the response phase (ShiftSNR), obtained by subtracting the mean eye position during the presentation of the second number from MeanR. We expected strong effects of OKS on such indices by design. Specifically, and perhaps counterintuitively, the mean position of the eyes is known to be tilted towards the direction which is opposite to the direction of optic flow (beating field; Watanabe, 2001). For example, rightward stimulation is associated to leftward center of gravity, even if attentional shifts occur away from it (Pizzamiglio et al., 1990). Note, however, that the contrast of interest is not within the different OKS directions, but rather within the different operation types. If arithmetical operations are performed along a mental number line, with subtractions linked with a leftward/downward movement and additions with a rightward/upward movement, then, within each OKS condition, the two operations should independently influence oculomotor control as revealed by gaze position. Therefore, the smallest MeanR and ShiftSNR values were expected for subtractions, indicating a leftward bias, while additions should be linked with higher values, indicating a rightward bias (downward or upward, respectively, when considering the vertical axis).

As a control analysis we also considered gaze indices for a phase of the trial in which no effect of Operation Type is expected. In particular, we computed the center of gravity during the presentation of the first number (MeanFN) and the mean shift observed from the alert sound to the first number presentation phase (ShiftAFN, see Fig. 1, panel B).^2^

Other data exclusions depended on eye-tracker failures in recording a sufficient number of gaze samples at each phase of the trial (namely: alert, first number, operator, second number, and response phase). First, 3.22% of trials in which less than 70% of gaze data were collected during the response phase were discarded. Then, from the remaining phases, further exclusions were made stepwise when analysing ShiftSNR (1.94% additional trials discarded because insufficient data was collected during the presentation of the second number), MeanFN (1.13%, insufficient data during the presentation of the first number) or ShiftFNA (2.68%, insufficient data during either the presentation of the first number or the alert phase).

All eye-movement indices were submitted to a 3 × 2 ANOVA (OKS x Operation Type).

#### Results

##### Horizontal axis

Not surprisingly, when submitting MeanR and MeanFN to ANOVA the main effect of OKS was significant (for MeanR: *F*(2,46) = 7.69, *p* = 0.001, *η*_p_^2^ = 0.25, BF = 25,701; for MeanFN: *F*(2,46) = 24.45, *p* < 0.001, *η*_p_^2^ = 0.515, BF = 3.7_10_^16^): the mean eye position was tilted towards the beating field. The effect of Operation Type was not significant for both MeanR (*F*(1,23) = 3.66, *p* = 0.068, *η*_p_^2^ = 0.137, BF = 0.2) and MeanFN (*F*(1,23) = 1.93, *p* = 0.178, *η*_p_^2^ = 0.077, BF = 0.18); no interactions were found (all *ps* > 0.1, BFs < 0.13).

**Fig. 5:**
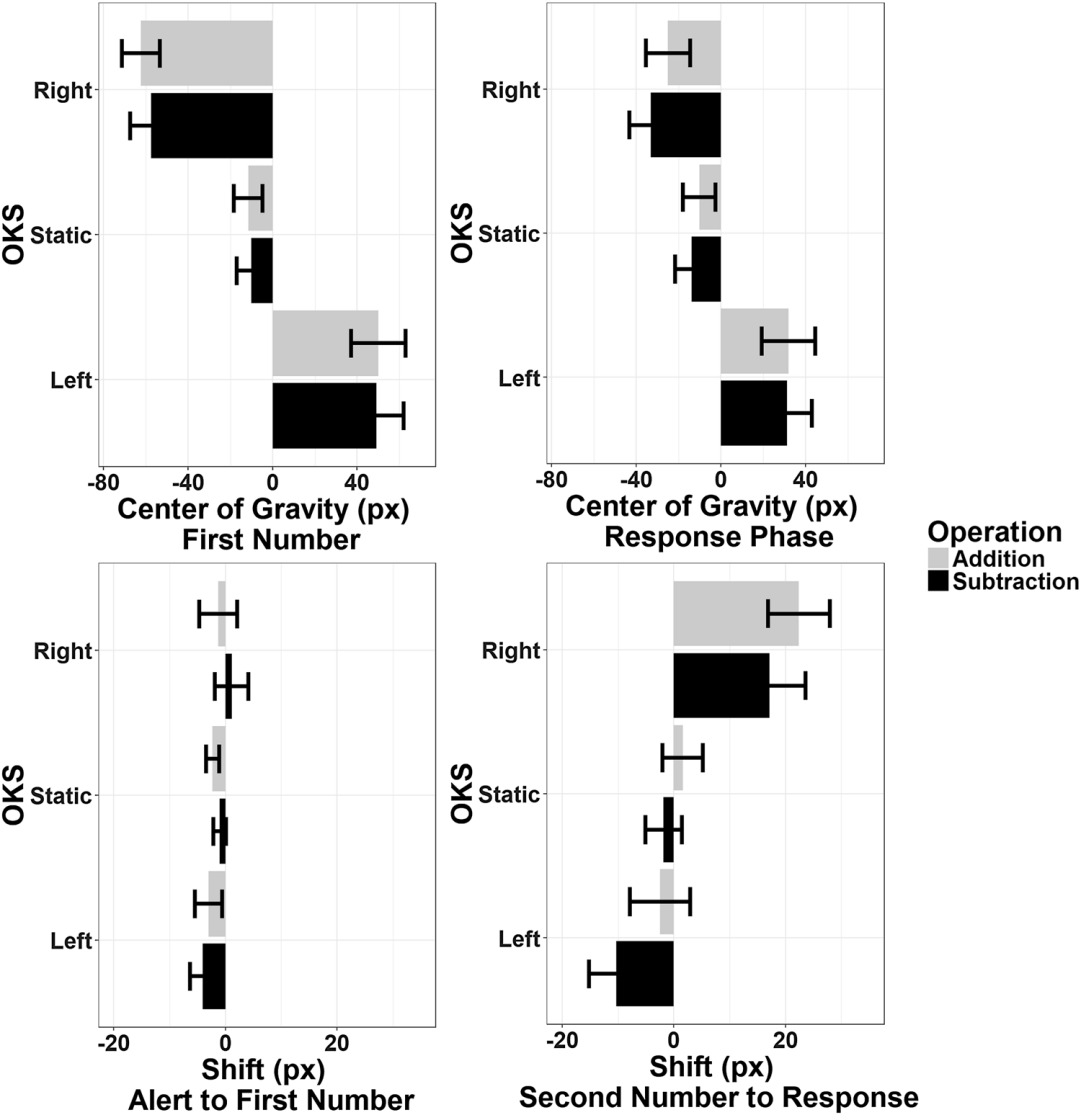
Pattern of eye movements along the horizontal axis. Top panels: mean position of the eyes (in pixels with respect to the center) during the presentation of the first operand and during the response phase. Note that the center of gravity is, as expected, tilted towards the OKS beating field, i.e., the direction opposite to pursuit eye movements and attentional shifts. Bottom panels: mean shift of gaze position in the same phases with respect to the preceding ones. The reference for the first operand phase was the presentation of the alert tone, whereas for the response phase the reference was the presentation of second operand. Negative values indicate leftward displacement and positive values a rightward one. Note that gaze position shifts during the response phase only, with a leftward shift of the eyes during subtraction and a rightward shift during addition regardless of OKS. Error bars represent within-subjects SEM (Morey, 2008)

The same analysis on the displacement (shift) indices yielded a significant effect of Operation Type for ShiftSNR (*F*(1,23) = 9.37, *p* = 0.006, *η*_p_^2^ = 0.29, BF = 0.35), suggesting a leftward displacement of the eye position during subtractions and a rightward displacement during additions in the response phase only (see Fig. 5). This effect was not present during the corresponding control (i.e., non-calculation) phase [ShiftAFN: *F*(1,23) = 0.43, *p* = 0.52, *η*_p_^2^ = 0.018, BF = 0.19]. The main effect of OKS was also present during the response phase only [ShiftSNR: *F*(2,46) = 7.21, *p* = 0.005—Greenhouse–Geisser corrected, *η*_p_^2^ = 0.24, BF = 17,646; ShiftAFN: *F*(2,46) = 0.79, *p* = 0.45, *η*_p_^2^ = 0.033, BF = 0.18], but with no interactions with Operation Type (all *p*s > 0.6, BFs < 0.15).

##### Vertical axis

**Fig. 6:**
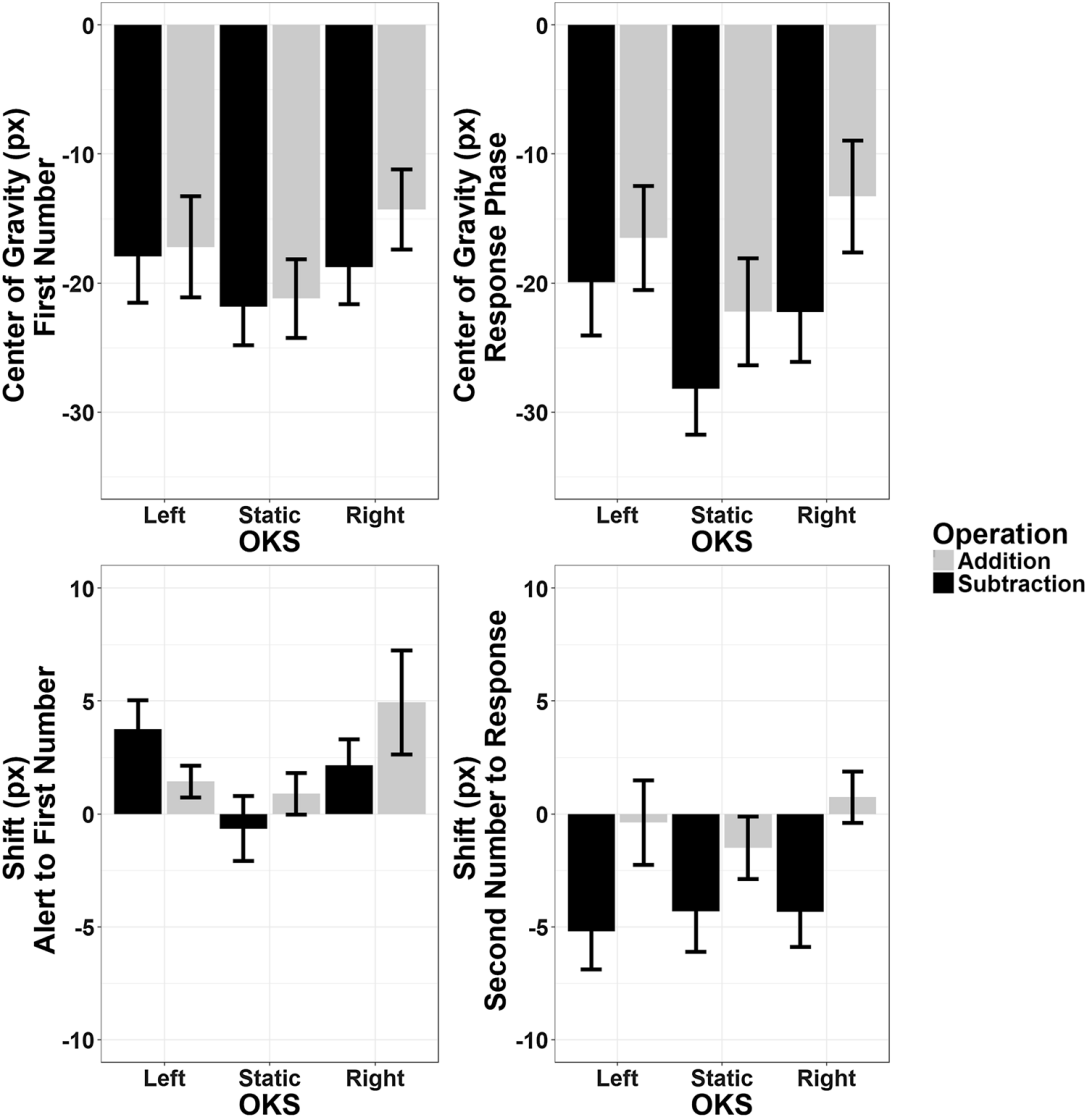
Pattern of eye movements along the vertical axis. Top panels: mean position of the eyes (in pixels with respect to the center) during the presentation of the first operand and during the response phase. Bottom panels: mean shift of gaze position in the same phases with respect to the preceding ones. Negative values indicate downward displacement and positive values an upward one. Note that gaze position shifts during the response phase only, with a downward shift of the eyes during subtraction and an upward shift during addition. Error bars represent within-subjects SEM (Morey, 2008)

Analysis of MeanFN and MeanR showed no effect of OKS (*p* = 0.58 and *p* = 0.395, respectively, BFs < 0.4). Nevertheless, the effect of Operation Type was significant for MeanR (*F*(1,23) = 16.5, *p* < 0.001, *η*_p_^2^ = 0.42, BF = 0.83), suggesting an overall downward displacement of the eyes position during subtractions with respect to additions (see Fig. 6).

Results were corroborated by the analysis of the displacement indices. ShiftAFN showed a significant effect of OKS, *F*(2,46) = 3.8, *p* = 0.03, *η*_p_^2^ = 0.141, BF = 1.26, but no effect of Operation Type, *F*(1,23) = 0.29, *p* = 0.59, *η*_p_^2^ = 0.013, and BF = 0.2. Conversely, ShiftSNR showed a significant effect of Operation Type, *F*(1,23) = 12.51, *p* = 0.002, *η*_p_^2^ = 0.352, and BF = 20.88, but no effect of OKS, *F*(2,46) = 0.25, *p* = 0.78, *η*_p_^2^ = 0.01, BF = 0.08). No interaction was found to be significant (all *p*s > 0.2, BFs < 0.5).

### Discussion

Horizontal nystagmus was not found to modulate participants’ responses (and specifically the distribution of errors). On the other hand, we observed that eye movements were affected by the type of operation, subtraction leading to leftward and downward displacement of gaze position, and addition leading to rightward and upward shifts. The effect of the arithmetic operation on the vertical axis (that was not explicitly manipulated so far, as the OKS was horizontal) prompted the second experiment, in which we administered a vertical stimulation.

## Experiment 2

Experiment 1 provided evidence for a link between Operation Type and oculomotor behaviour in the vertical dimension, with additions leading to upward movements of the eyes with respect to subtractions. In Experiment 2, we administered vertical OKS to explore whether the explicit manipulation of spatial attention along the vertical axis may affect mental calculation and modulate the distribution of responses.

### Participants

Participants of experiment 2 were enrolled with the same modalities of experiment 1. The sample was composed by 24 participants (as for Exp. 1), 7 males (29%), mean age was 24.62 years (range 20–32, SD 3.11), mean years of formal education were 14.45 (range 13–18, SD 1.66).

### Apparatus, stimuli, and procedure

Setting, stimuli, and procedures were identical to experiment 1, except for the directions of OKS: we administered downward and upward OKS, in addition to the static/baseline condition. Counterbalancing remained the same as well.

### Behavioural data

#### Description of indices and analysis

The mean shift from the correct result was computed. We excluded deviant eye movements (9.23%); differently from Experiment 1, participants were free to wander along the vertical axis but confined to the 2/3 of the horizontal axis length. This was to ensure that a vertical optokinetic nystagmus was triggered. Trials in which participants did not respond were also discarded (0.07%). Finally, trials in which decade errors were made were also discarded (9.7% of all responses), to operationally isolate estimation errors from procedural ones.

#### Results

##### Accuracy and RTs

Analyses of accuracy (see Figure **S3**) showed an advantage for additions over subtractions (main effect of Operation type: *F*(1,23) = 37.14, *p* < 0.001, *η*_p_^2^ = 0.62); there were no other effects due to OKS or its interaction with Operation type (all *ps* > 0.35). When assessing response times (see Figure **S4**), 3 participants had to be removed from analyses because they did not provide enough correct answers in one of the Operation type by OKS cells. The analysis highlighted an effect of Operation type (*F*(1,20) = 5.82, *p* = 0.026, *η*_p_^2^ = 0.23) consisting, coherently with accuracy results, in an advantage in solving additions with respect to subtractions. On the other hand, there were no effects of OKS or the interaction OKS by Operation type (all *ps* > 0.1).

##### Deviation

**Fig. 7:**
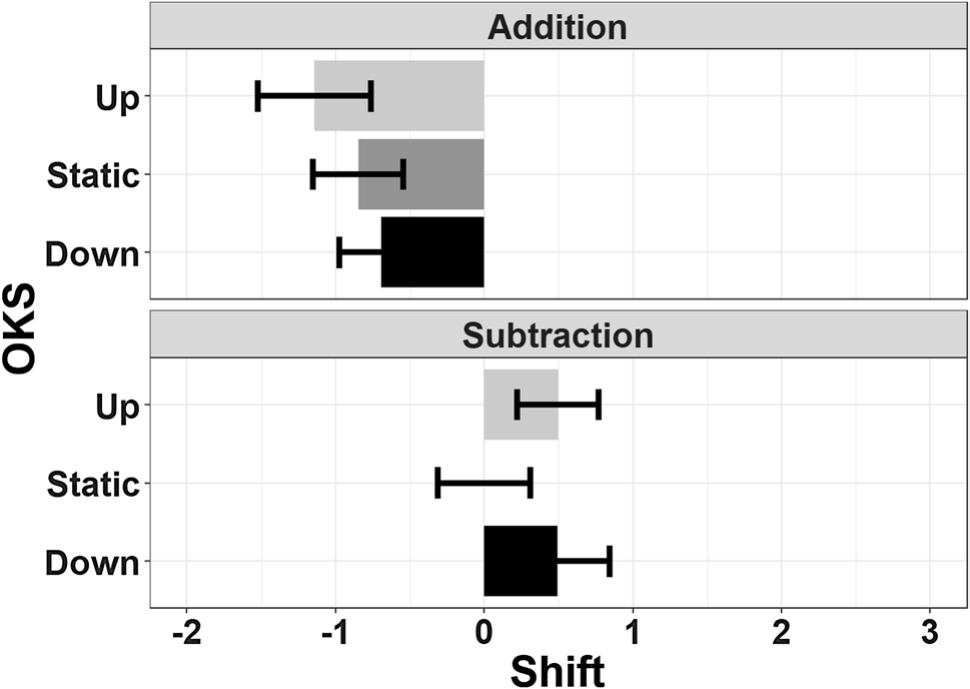
The mean estimation error is depicted as a function of Operation Type and OKS in Experiment 2. The pattern of underestimation for addition and overestimation for subtraction mirrors the results of Experiment 1. No effects of vertical OKS were found. Error bars represent within-subjects SEM (Morey, 2008)

The main effect of Operation Type was significant (*F*(1,23) = 8.04, *p* = 0.009, *η*_p_^2^ = 0.259, BF = 4890), as in Experiment 1, with addition showing a more pronounced underestimation of results with respect to subtraction. Neither the main effect of OKS (*F*(2,46) = 0.85, *p* = 0.43, *η*_p_^2^ = 0.035, BF = 0.1) nor the Operation Type by OKS interaction (*F*(2,46) = 1.62, *p* = 0.2, *η*_p_^2^ = 0.066, BF = 0.2) were significant (Fig. 7).

##### Decade errors

For positive decade errors, the main effect of Operation Type was significant (*F*(1,23) = 13.62, *p* = 0.001, *η*_p_^2^ = 0.372, BF = 240). There was no main effect of OKS (*F*(2,46) = 0.75, *p* = 0.48, *η*_p_^2^ = 0.03, BF = 0.11), but the OKS by Operation Type interaction was also significant (*F*(2,46) = 6.3, *p* = 0.004, *η*_p_^2^ = 0.215, BF = 4.15). Follow-up *t* test comparisons (Bonferroni corrected) showed that the effect of Operation Type (with subtractions yielding an overall increased rate of positive decade errors) was abolished by downward OKS (*t*(23) = 0.159, *p* = 1, BF = 0.22), whereas it was present in the static (*t*(23) = 2.99, *p* = 0.022, BF = 6.94) and upward OKS (*t*(23) = 3.86, *p* = 0.002, BF = 42.64). The null effect of Operation Type during downward OKS is supported by a Bayes Factor of 4.6 (1/0.22).

**Fig. 8:**
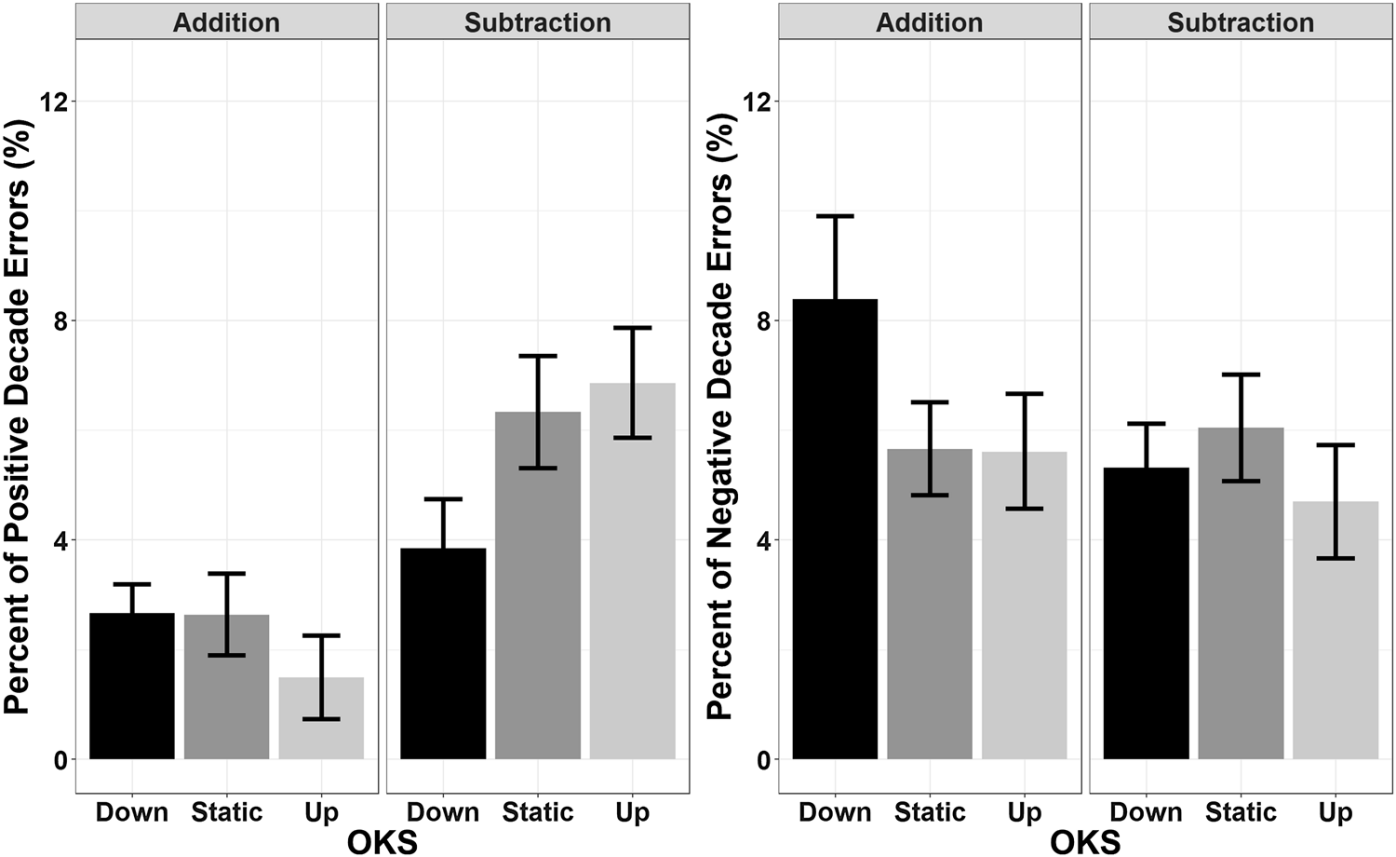
The percentage of decade errors (i.e., responses deviating from the correct result by a multiple of 10 units) across all trials is depicted as a function of Operation Type and OKS. Left panel: negative decade errors: right panel: positive decade errors. Positive decade errors were more frequent in subtractions, but they were also modulated by vertical OKS, with downward OKS abolishing this effect with respect to static and upward OKS. Error bars represent within-subjects SEM (Morey, 2008)

No significant effects were found for negative decade errors (Operation Type: *F*(1,23) = 0.99, *p* = 0.33, *η*_p_^2^ = 0.04, BF = 0.32; OKS: *F*(2,46) = 0.45, *p* = 0.63, *η*_p_^2^ = 0.02, BF = 0.1; interaction: *F*(2,46) = 1.65, *p* = 0.2, η_p_^2^ = 0.07, BF = 0.3). All results are depicted in Fig. 8.

### Eye-tracking data

#### Description of indices and analyses

When considering eye-tracking data, we adopted as dependent variables both the mean position of the eyes along the horizontal or vertical axis of the screen and the shift in eye position occurring between different phases of the trial, as in Experiment 1. Deviant eye movements (9.23%), and trials in which no gaze data were collected during the response phase (0.26%) were discarded. Then, exclusions were made stepwise when analysing ShiftSNR (0.48% of exclusions due to insufficient data during the presentation of the second number), MeanFN (0.6% of insufficient data when presenting the first number) or ShiftFNA (2.24% of insufficient data when presenting either the first number or the alert sound).

#### Results

##### Horizontal axis

No main effects or interactions were found to be significant in the ANOVAs on MeanR, MeanFN, and ShiftAFN (main effect of Operation Type: *ps* > 0.2, BFs < 0.2).

The analysis on ShiftSNR (please refer to Figure **S5** for graphical depiction) yielded a significant main effect of OKS (*F*(2,46) = 6.27, *p* = 0.004, *η*_p_^2^ = 0.21, BF = 127): both moving OKS conditions, with respect to the static one, were associated to a rightward shift of the eyes during the final stage of the calculation only. The effect of Operation Type was not significant (*F*(1,23) = 1.23, *p* = 0.28, *η*_p_^2^ = 0.05, BF = 0.28), nor was its interaction with OKS (*F*(2,46) = 0.16, *p* = 0.81, *η*_p_^2^ = 0.007, BF = 0.125).

##### Vertical axis

No main effect of OKS was found on MeanFN and MeanR. For the latter only there was an effect of Operation Type (*F*(1,23) = 6.7, *p* = 0.016, *η*_p_^2^ = 0.226, BF = 0.19), suggesting an overall downward displacement of the eye position during subtractions and an upward displacement during additions only during a late stage of the trial. No other effects or interactions were significant.

**Fig. 9:**
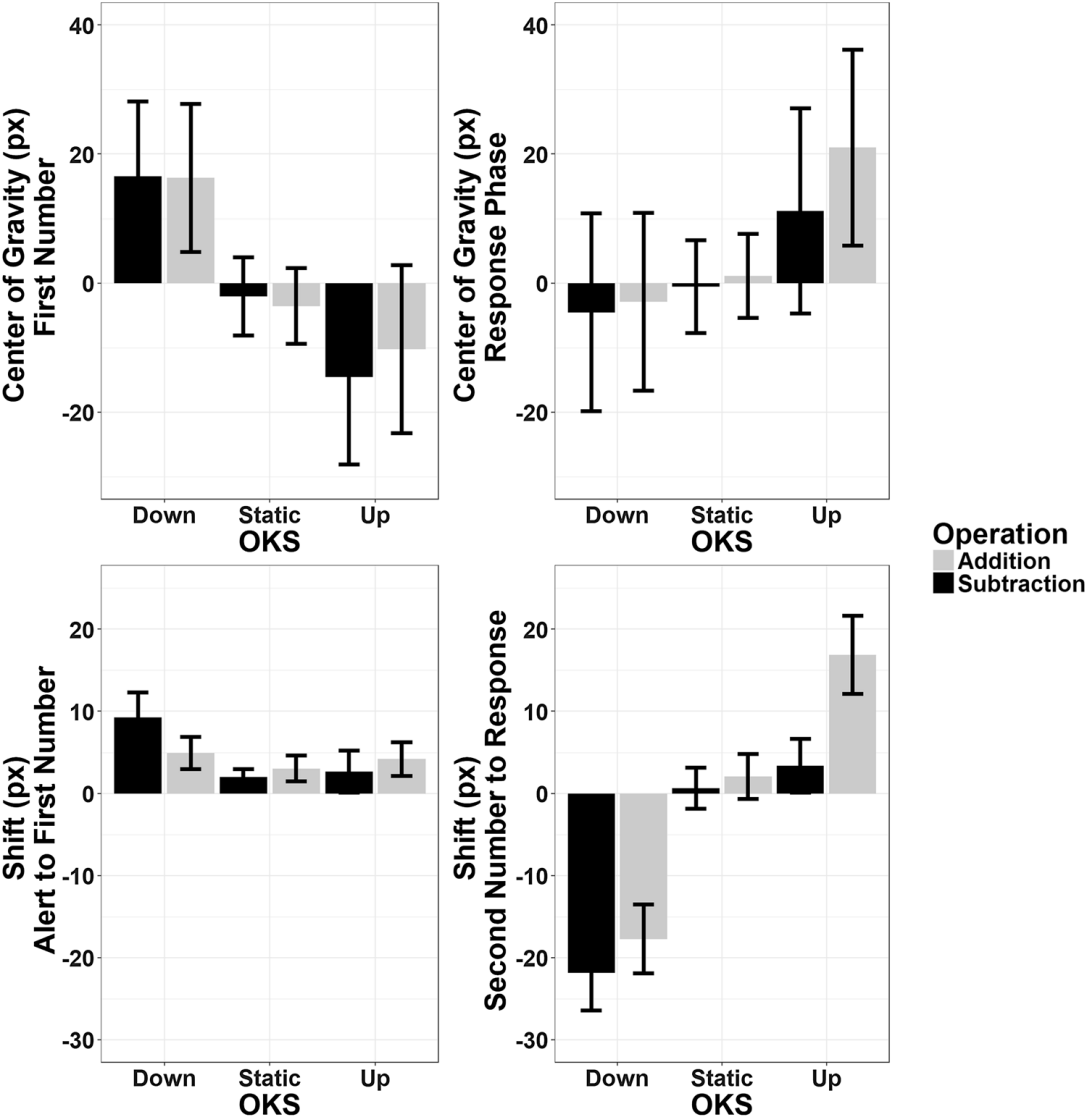
Pattern of eye movements along the vertical axis. Top panels: mean position of the eyes (in pixels with respect to the center) during the presentation of the first operand and during the response phase. Bottom panels: mean shift of gaze position in the same phases with respect to the preceding ones. Gaze position shifts during the response phase only, with a downward shift of the eyes during subtraction and an upward shift during addition; an interaction with OKS was also found, suggesting that this effect is only observed during upward optokinetic stimulation. Error bars represent within-subjects SEM (Morey, 2008)

The effect of Operation Type was corroborated by the analysis of the displacement indices, which was consistent for both ShiftAFN and ShiftSNR (main effect of Operation Type: *F*(1,23) = 0.12, *p* = 0.72, *η*_p_^2^ = 0.005, BF = 0.19 and *F*(1,23) = 16.11, *p* = 0.001, *η*_p_^2^ = 0.41, BF = 0.65, respectively). ShiftSNR also showed a significant OKS main effect (*F*(2,46) = 1.41, *p* = 0.25, *η*_p_^2^ = 0.057, BF = 0.66 and *F*(2,46) = 16.15, *p* < 0.001, *η*_p_^2^ = 0.41, BF = 1.68_10_^9^, respectively; Greenhouse–Geisser correction was applied in the latter case) showing that, during these late stages, eye gaze was more easily shifted towards the OKS direction. Finally, the interaction between OKS × Operation Type was only significant when analysing ShiftSNR (*F*(2,46) = 4.34, *p* = 0.019, *η*_p_^2^ = 0.159, BF = 0.23, Greenhouse–Geisser correction applied); post hoc (Bonferroni corrected) analysis showed that the Operation Type effect was present during upward OKS (*t*(23) =  3.07 *p* = 0.015, BF = 8.25) but not during downward OKS (*t*(23) = 1.82, *p* = 0.243, BF = 0.89) or at baseline (static condition, *t*(23) = 1.13, *p* = 0.81, BF = 0.38) (Fig. 9).

### Discussion

We have found that vertical OKS modulates participants’ responses and their distribution. Specifically, procedural, but not estimation errors, were significantly modulated so that positive decade errors (i.e., an overestimation that was a multiple of ten units, mostly seen during subtraction) were enhanced with upward stimulation and reduced with a downward one. When assessing eye movements we could not replicate the oculomotor bias found in the horizontal plane found in experiment 1, but we confirmed that subtraction leads to an overall downward shift of eye position with respect to addition.

## General discussion

In the present study we explored the effects of OKS, a technique that induces shifts of spatial attention secondary to shifts in eye movements (optokinetic nystagmus), on mental calculation. Our aim was to investigate both the potential modulation of arithmetic performance by OKS (from spatial attention/eye movements to the calculation domain) and the complementary effects of operation type (subtractions vs. additions) on eye movements (from the calculation domain to eye movements/spatial attention). As noted in the introduction, vertical OKS was used for the first time in the present study because assessing its effect (also in comparison to horizontal OKS) is of primary theoretical significance for embodied accounts of number processing and calculation.

The results of the arithmetic task, in terms of accuracy of response, showed an overall pattern of underestimation for additions and overestimation for subtractions. Note that this is the opposite with respect to the classic OM effect, which consists in overestimation of the result during addition and underestimation during subtraction (McCrink et al., 2007). The first important difference between our study and many others in literature is the modality of both stimuli presentation and response, which is auditory/verbal rather than visual/motor. Linguistic stimuli are thought to recruit partly different mechanisms and cognitive strategies (Hubbard, 2014); studies employing verbal stimuli often found “pseudoneglect” for mental number space (Göbel, Calabria, Farnè, & Rossetti, 2006; Loftus, Nicholls, Mattingley, Chapman, & Bradshaw, 2009; Lourenco & Longo, 2010), namely an overall tendency to underestimate the correct result. Interestingly, studies exploring the effect of scanning direction in visual line bisection indicate that “pseudoneglect” in physical space (leftward bisection error) is only found (or it is stronger) when the visual line is canonically explored from left to right, whereas forced leftward scanning typically leads to rightward bisection errors (or smaller “pseudoneglect”) (Brodie & Pettigrew, 1996; Chokron, Bartolomeo, Perenin, Helft, & Imbert, 1998; one of the largest performance modulator according to the meta-analysis by Jewell & McCourt, 2000). Given that subtractions could be characterized by a leftward movement along the MNL, the observed overestimation may be explained in light of this different “scanning” direction (right to left). This spatial account could provide a comprehensive description of our results, but it fails in describing cases in which the OM manifest itself in its canonical direction. Furthermore, the lack of modulation of this effect by OKS might also cast doubts on its spatial origin.

An alternative, non-spatial account of the results is offered by the anchoring effect (Tversky & Kahneman, 1975). In their seminal study, Tversky and Kahneman found that participants’ responses to a complex mental arithmetic problem are strongly modulated by the size of the first operand, yielding higher vs. lower estimates by simply swapping the order of the operands. Though the overall magnitude of the arithmetic problems was balanced across operations in our stimulus set, the first operand was more often larger for subtraction than for addition. As a result, anchoring on the first operand would lead to overestimation for subtraction (i.e., a result closer to the high number anchor) and underestimation for addition (i.e., a result closer to the low number anchor). Thus, the lack of a classic OM might eventually be due to the specific list of items adopted in our study. Note that at least one study showed the classic OM with a list of stimuli balanced for the magnitude of the result (Knops, et al., 2009, experiment 2); this would seem to be at odds with the anchoring effect. However, a reverse OM effect has also been found in recent studies of symbolic arithmetic for non-zero addition and subtraction problems (Shaki, Pinhas, and Fischer, 2017; also see Shaki and Fischer, 2017). The authors framed the OM effect in the context of at least three competing mental biases and heuristics: the more-or-less heuristic reflects ecological (grounded) associations of additions with larger outcomes and subtractions with smaller outcomes; the sign–space association captures a culturally driven tendency to associate additions with the right space and subtractions to the left space; finally, non-spatial biases such as the anchoring effect also have a preeminent role and, under certain conditions, prevail on spatial biases (Shaki, Pinhas, and Fischer, 2017). Crucially, this framework specifically predicts a reverse OM, at least for non-zero second operands, in line with our results. The lack of a modulation of this effect by OKS, furthermore, corroborates the notion that spatial biases may only have a marginal role in symbolic arithmetic, overruled by non-spatial ones.

Though many errors were distributed within a few units from the correct result, as expected for numerical estimation, we also observed a sizable portion of procedural errors, that is, responses that differed from the correct ones at the tens position (decade errors). This is in line with the evidence that procedural knowledge is important for mental addition and subtraction with two-digit numbers. Most notably, we found that vertical OKS influenced the probability of procedural errors during mental calculation: positive decade errors were more frequent for subtraction with respect to addition, but downward OKS abolished this bias. These results suggest that the procedures triggered by multi-digit calculation rely, to some extent, on spatial/attentional processes, in agreement with neuropsychological observations (Ardila & Rosselli, 1994; de Hevia et al., 2008, for review). Indeed, a spatial component has also been identified for procedural errors, for example, when carrying and borrowing procedures are required and numbers should be arranged in “mental columns”. Subtractions require borrowing; additions require carry. An error in the procedure requiring borrowing (e.g., leftovers are neglected, borrowing is not performed) yields more often positive decade errors; an error occurring in the carry procedure yields more often negative decade errors. Therefore, the similarity between the pattern of procedural (positive decade errors for subtraction and negative decade errors for addition) and the pattern of estimation errors (overestimation for subtractions, underestimation for additions) does not imply a shared origin, and does not necessarily relate to the MNL framework. Dissociations between procedural and estimation strategies have been reported in neuropsychological studies (Ardila & Rosselli, 1994; Boller & Grafman, 1983; de Hevia, Vallar, & Girelli, 2008, for review) and indeed we observed one in the present study in terms of the modulating effect of OKS.

In the recent study of Masson et al. (2016), rightward OKS was found to induce a relative speed-up of addition problems requiring a carrying procedure. Horizontal OKS had no effect in the present study, but it must be stressed that our calculation task was more difficult compared to that of Masson et al. (which involved adding/substracting a one-digit number to/from a two-digit number). As previously noted, there is variability in the procedures that can be used for addition and subtraction of two-digit numbers, which implies less reliability of response times; in addition, we explicitly favoured problems that were likely to yield error responses (more than half of the trials in some conditions, either estimation or decade errors), as to better assess error distribution, and as a consequence only a few trials per cell were left to compute response times. In our study, therefore, RTs should be interpreted with much caution because they are likely to be very noisy and unreliable. On top of this, additionally, our list of stimuli was not optimized to be split according to whether a carrying/borrowing procedure was involved (i.e., problems were unbalanced), and using this information as an additional factor in the analyses was simply not possible in light, again, of the insufficient number of observations per cell. Overall, it is our view that our study does not detract from that of Masson et al. because it is far from replicating its procedures, analyses, and experimental hypotheses. On the contrary, we suggest that both our results and those of Masson et al. represent a signature of the spatial/attentional processes that take place during mental calculation: attentional shifts occurring in a direction that is compatible to the putative movement along the MNL (e.g., downward for subtractions) may have facilitatory effects on mental calculation that extend to procedural components. Finally, it is conceivable that we observed an effect of vertical OKS, but not of horizontal OKS, because addition and subtraction might be grounded in early experiences with the physical world along the vertical axis, such as the universal (and culture-independent) actions of stacking and removing items from a pile (Fischer, 2012; Myachykov et al., 2014; but see Liu, Verguts, Li, Ling, & Chen, 2017, for contrasting results).

The role of spatial attention in numerical cognition is still hotly debated. Several non-spatial accounts have been proposed as alternative explanations for number–space interactions (e.g., Aiello et al., 2012; Fattorini, Pinto, Merola, D’Onofrio, & Doricchi, 2016; Fias, van Dijck, & Gevers, 2011; Proctor & Cho, 2006). However, there is now a wealth of studies showing that numbers or magnitudes (Blini et al., 2013; Cattaneo, Fantino, Mancini, Mattioli, & Vallar, 2012; Fischer et al., 2003; Ishihara, Jacquin-Courtois, Rode, Farnè, & Rossetti, 2013) or arithmetical operations (Hartmann, Mast, & Fischer, 2015; Klein et al., 2014; Liu et al., 2017; Masson & Pesenti, 2014; Pinhas & Fischer, 2008) may induce shifts of spatial attention (but see also Fattorini, Pinto, Rotondaro, & Doricchi, 2015; Zanolie & Pecher, 2014, for failed replication attempts). A second growing line of evidence is also contributing, in the other way around, by manipulating explicitly spatial attention to explore its potential influence on number processing. For example, Stoianov, Kramer, Umiltà, and Zorzi (2008) found that irrelevant lateralized visuospatial cues can influence number processing, with faster response times (RTs) to small numbers following left cues compared to right cues and the opposite pattern for large numbers (also see Kramer, Stoianov, Umiltà, & Zorzi, 2011). Similar results were observed by Hartmann, Grabherr, and Mast (2012, experiment 2) using a vestibular stimulation (which is known to affect the metric of spatial representations) during a magnitude comparison task (see also Loetscher, Schwarz, Schubiger, & Brugger, 2008). Ranzini et al. (2015) administered optokinetic stimulation (OKS) to healthy participants during number processing and observed that number comparison RTs were modulated by OKS direction (see also Ranzini, Lisi, & Zorzi, 2016). These effects seem to extend to simple arithmetic (Masson & Pesenti, 2016; Masson et al., 2016). Besides being in line with these observations, the present study joins for the first time the two lines of research by assessing concurrently both the effect of overt shifts of attention/eye movements on mental calculation and its reciprocal effect (i.e., the effect of solving arithmetic operations on oculomotor control), overall adding compelling evidence on the tight coupling between the number space and eye movements.

In this regard, our results on the eye movements data are clear-cut, and corroborate recent studies (Hartmann, Mast, & Fischer, 2015), including the finding of Knops et al. (2009) that saccade-related activity in the posterior parietal cortex was a good predictor of the type of mental operation participants were engaged into. In our study, subtractions, with respect to additions, were linked to a leftward displacement of gaze position, rightward for additions, when horizontal OKS was administered. Notably, this displacement was only observed during the response phase and not at other points of the trial, possibly because the MNL was activated only in the calculation phase (in line with Liu et al., 2017, and Masson et al., 2017). Moreover, the type of arithmetic operation had also an effect on the vertical dimension, with subtractions consistently linked to an overall downward displacement of the eyes with respect to additions. The latter effect was highly reliable—in contrast to that on the horizontal plane—and it was observed across experiments (independently of the type of OKS). These findings are in line with the predictions of a hierarchical model of number–space interactions (Fischer, 2012; Myachykov et al., 2014), which suggests that the vertical dimension is grounded in universal (physical) constraints (as opposed to the horizontal dimension, which is situated and culturally dependent; but see Liu, Verguts, Li, Ling, & Chen, 2017, for contrasting results). One caveat of the present study is that we cannot disentangle whether the modulation of eye movements observed during the calculation reflect the intrinsic calculation process or the magnitude of the result. Both factors might play a role (Klein et al., 2014; Loetscher, Bockisch, Nicholls, & Brugger, 2010), though recent studies suggest that the former has the major impact (Hartmann et al., 2015). In this regard, it is worth noticing that we did not observe spatial displacement of eye position in the time window corresponding to the processing of the first operand, despite the fact that it was typically, albeit not always, larger for subtractions than for addition (Liu et al., 2017; Masson, Letesson, et al., 2017). This corroborates the view that numerical magnitude might retain only a marginal effect on oculomotor behaviour during calculation. Finally, one may wonder if task difficulty could be at the roots of the overall downward displacement of gaze position during mental subtraction. We are not in the position to provide an answer on the basis of our data, as in this study subtractions were more difficult than addition problems (i.e., the former presented lower accuracy and slower response times than the latter). Other studies using much easier problems sets (e.g., operands < 10, Hartmann et al., 2015) or counting upward/downward by 3 (Hartmann, Mast, & Fischer, 2016) found similar results (i.e., additions, with respect to subtractions, leading to upward shifts of gaze position). However, behavioural measures revealed faster RTs for additions with respect to subtractions also in some of these cases (Hartmann et al., 2015), and indeed this difference in difficulty is an established finding in cognitive arithmetic (Ashcraft, 1992). It is important for future studies to be aware of this possible confound, and possibly create stimuli set that are roughly balanced for difficulty.

In summary, overt orienting of spatial attention/eye gaze along the vertical axis influenced the participants’ responses to two-digit mental arithmetic problems. Conversely, gaze position shifted during the calculation phase in a direction consistent with the type of operation. These results highlight the pervasive nature of spatial processing in mental arithmetic which, as neuropsychological studies suggest, may manifest itself through a range of diverse processes (and errors, e.g., procedural). Here we also provide evidence for the number space to be grounded in common sensorimotor mechanisms (i.e., eye movements). More studies are needed to shed light on these complex interactions; as a first step, we urge researchers to concurrently assess both the effects of spatial manipulations on mental arithmetic and their complementary effects (i.e., from the number space to oculomotor control or spatial attention).

### Electronic supplementary material

Below is the link to the electronic supplementary material.

Supplementary material 1 Fig. S1 Accuracy rate is depicted as a function of Operation Type and OKS. An advantage for additions over subtractions has been found, but no effect of OKS or interactions. Note the overall low success rate, due to the precautions taken to induce errors and thus better assess their distribution. Error bars represent within-subjects SEM (Morey, 2008) (PNG 22 KB)

Supplementary material 2 Fig. **S2**: Response times (RTs) are depicted as a function of Operation Type and OKS. There were no effects of Operation type, OKS or their interaction. Note, however, that RTs were calculated only for correct answers, which as seen in S1 were not common; this should cast caution about the reliability of RTs, calculated on the basis of very few trials. Error bars represent within-subjects SEM (Morey, 2008) (PNG 22 KB)

Supplementary material 3 **Fig. S3** Accuracy rate for Exp 2 is depicted as a function of Operation Type and OKS. An advantage for additions over subtractions has been found, but no effect of OKS or interactions. Error bars represent within-subjects SEM (Morey, 2008) (PNG 22 KB)

Supplementary material 4 **Fig. S4** Response times (RTs) for Exp 2 are depicted as a function of Operation Type and OKS. There were no effects of OKS, neither alone or in interaction with Operation type; Operation type, however, was significant and indicated faster responses for additions with respect to subtractions. Note, however, that RTs were calculated only for correct answers (and thus on the basis of very few trials) and three participants had to be discarded because they did not present enough observations. Error bars represent within-subjects SEM (Morey, 2008) (PNG 21 KB)

Supplementary material 5 Fig. S5 Pattern of eye movements along the horizontal axis. Top panels: mean position of the eyes (in pixels with respect to the center) during the presentation of the first operand and during the response phase. Bottom panels: mean shift of gaze position in the same phases with respect to the preceding ones. There was no effect of Operation type in modulating oculomotor behaviour along the horizontal axis, unlike in experiment 1. Error bars represent within-subjects SEM (Morey, 2008) (PNG 81 KB)

Supplementary material 6 (HTML 2378 KB)

Supplementary material 7 (HTML 2362 KB)

## Appendix

See Table 2.

**Table 2:** Stimuli used in the experiment

Stimulus	First number	Operator	Second number	Correct	Stimulus	First number	Operator	Second number	Correct
1	88	Minus	64	24	16	77	Minus	34	43
2	82	Minus	57	25	17	87	Minus	44	43
3	92	Minus	67	25	18	81	Minus	37	44
4	94	Minus	68	26	19	82	Minus	38	44
5	55	Minus	28	27	20	81	Minus	35	46
6	61	Minus	34	27	21	71	Minus	24	47
7	62	Minus	35	27	22	74	Minus	27	47
8	93	Minus	66	27	23	93	Minus	46	47
9	82	Minus	54	28	24	87	Minus	36	51
10	88	Minus	55	33	25	97	Minus	44	53
11	97	Minus	64	33	26	81	Minus	27	54
12	62	Minus	28	34	27	91	Minus	37	54
13	83	Minus	48	35	28	92	Minus	35	57
14	84	Minus	49	35	29	96	Minus	34	62
15	81	Minus	45	36	30	92	Minus	29	63
1	27	Plus	29	56	16	48	Plus	37	85
2	34	Plus	27	61	17	43	Plus	45	88
3	29	Plus	33	62	18	35	Plus	53	88
4	27	Plus	36	63	19	63	Plus	26	89
5	49	Plus	23	72	20	54	Plus	35	89
6	49	Plus	26	75	21	66	Plus	25	91
7	33	Plus	45	78	22	36	Plus	56	92
8	56	Plus	25	81	23	59	Plus	34	93
9	53	Plus	28	81	24	28	Plus	65	93
10	37	Plus	44	81	25	65	Plus	29	94
11	44	Plus	38	82	26	45	Plus	49	94
12	36	Plus	46	82	27	67	Plus	28	95
13	34	Plus	48	82	28	33	Plus	64	97
14	26	Plus	56	82	29	63	Plus	35	98
15	47	Plus	37	84	30	43	Plus	55	98

## Footnotes

1 Excluding correct trials from the analyses (Shift equal to zero) did not change the pattern of results.

2 These two indices were perfectly in line with indices obtained from other phases of the trial (e.g. during the presentation of the operator or of the second number), in line with Masson, Letesson, & Pesenti (2017), thus were the only retained for brevity reasons.
