# Supplementary figures and images for: Spatial grounding of symbolic arithmetic: an investigation with optokinetic stimulation
Source: Psychol Res. 2018 Jul 18;83(1):64–83. doi: 10.1007/s00426-018-1053-0 (PMC6373542; doi:10.1007/s00426-018-1053-0)

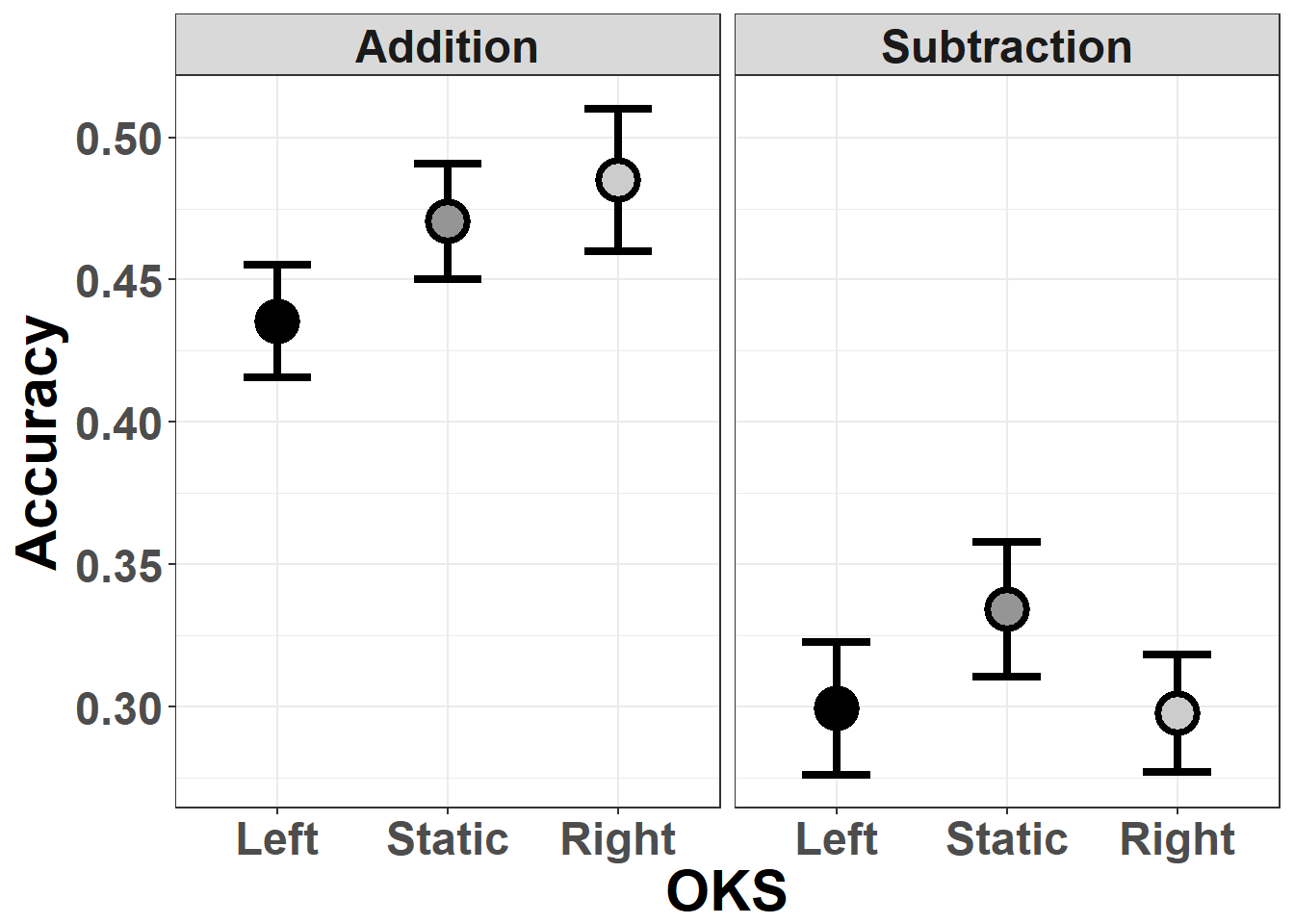

Supplement: Supplementary file 1 — Supplementary material 1 Fig. S1 Accuracy rate is depicted as a function of Operation Type and OKS. An advantage for additions over subtractions has been found, but no effect of OKS or interactions. Note the overall low success rate, due to the precautions taken to induce errors and thus better assess their distribution. Error bars represent within-subjects SEM (Morey, 2008) (PNG 22 KB) [file 426_2018_1053_MOESM1_ESM.png]

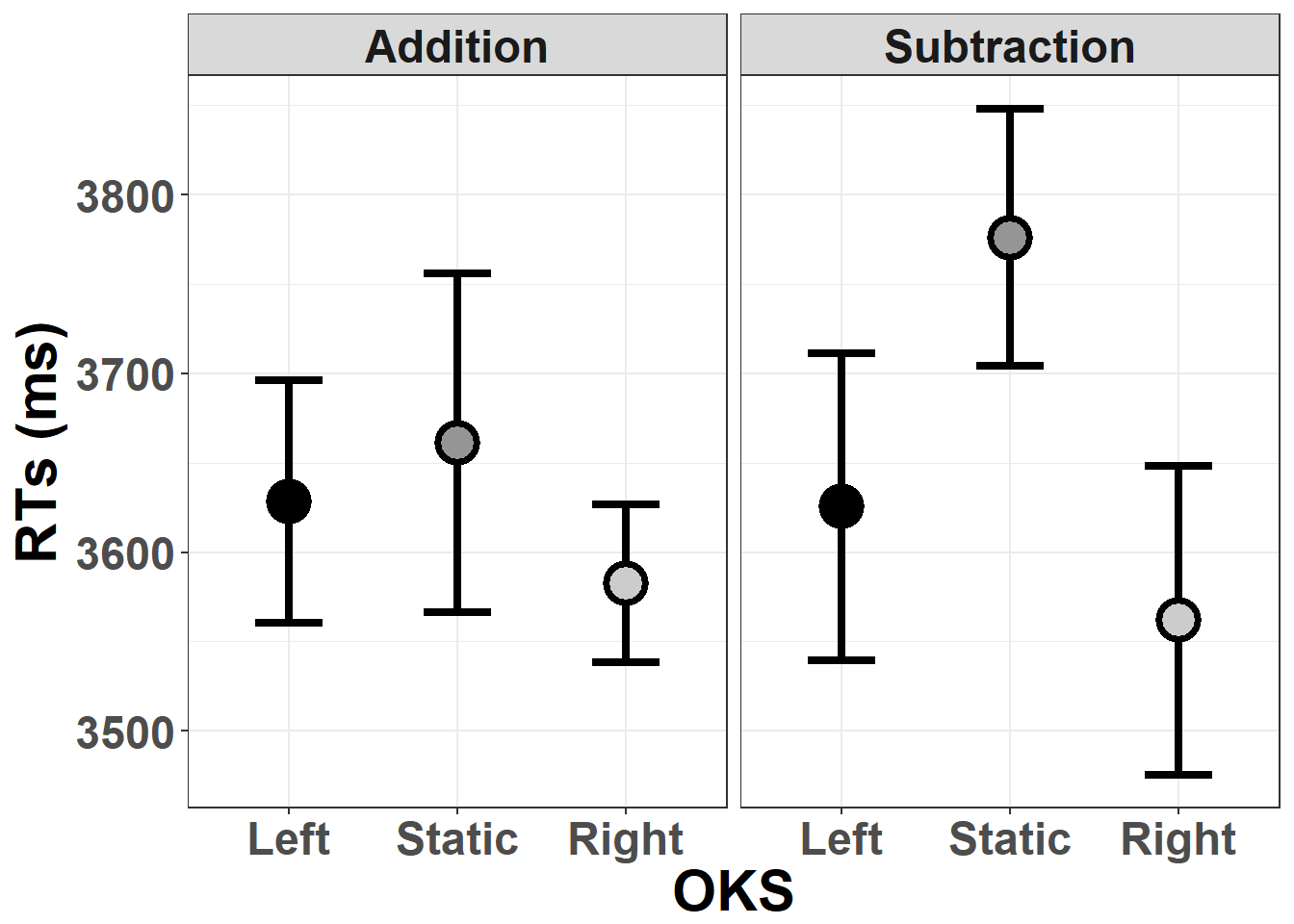

Supplement: Supplementary file 2 — Supplementary material 2 Fig. S2: Response times (RTs) are depicted as a function of Operation Type and OKS. There were no effects of Operation type, OKS or their interaction. Note, however, that RTs were calculated only for correct answers, which as seen in S1 were not common; this should cast caution about the reliability of RTs, calculated on the basis of very few trials. Error bars represent within-subjects SEM (Morey, 2008) (PNG 22 KB) [file 426_2018_1053_MOESM2_ESM.png]

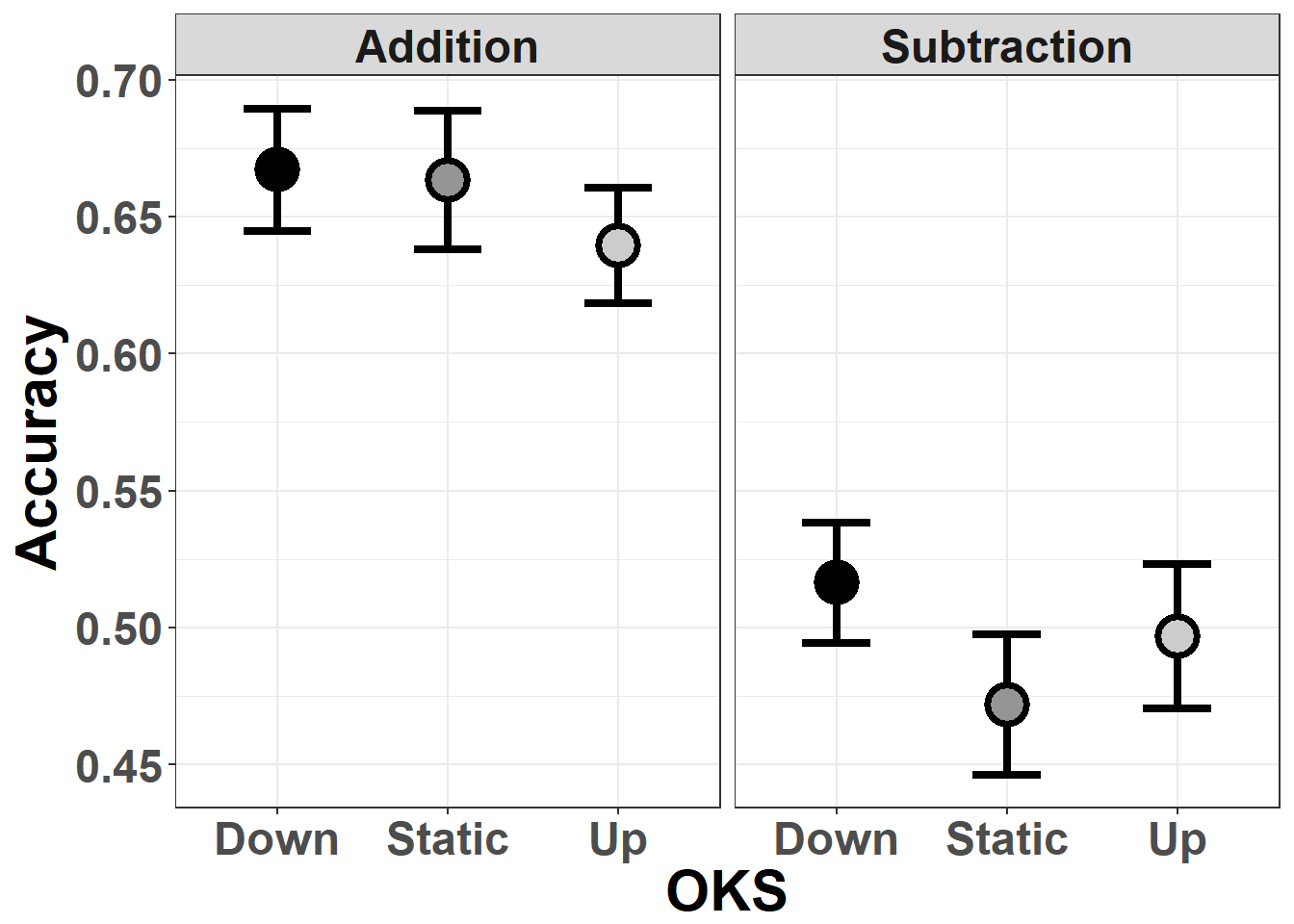

Supplement: Supplementary file 3 — Supplementary material 3 Fig. S3 Accuracy rate for Exp 2 is depicted as a function of Operation Type and OKS. An advantage for additions over subtractions has been found, but no effect of OKS or interactions. Error bars represent within-subjects SEM (Morey, 2008) (PNG 22 KB) [file 426_2018_1053_MOESM3_ESM.png]

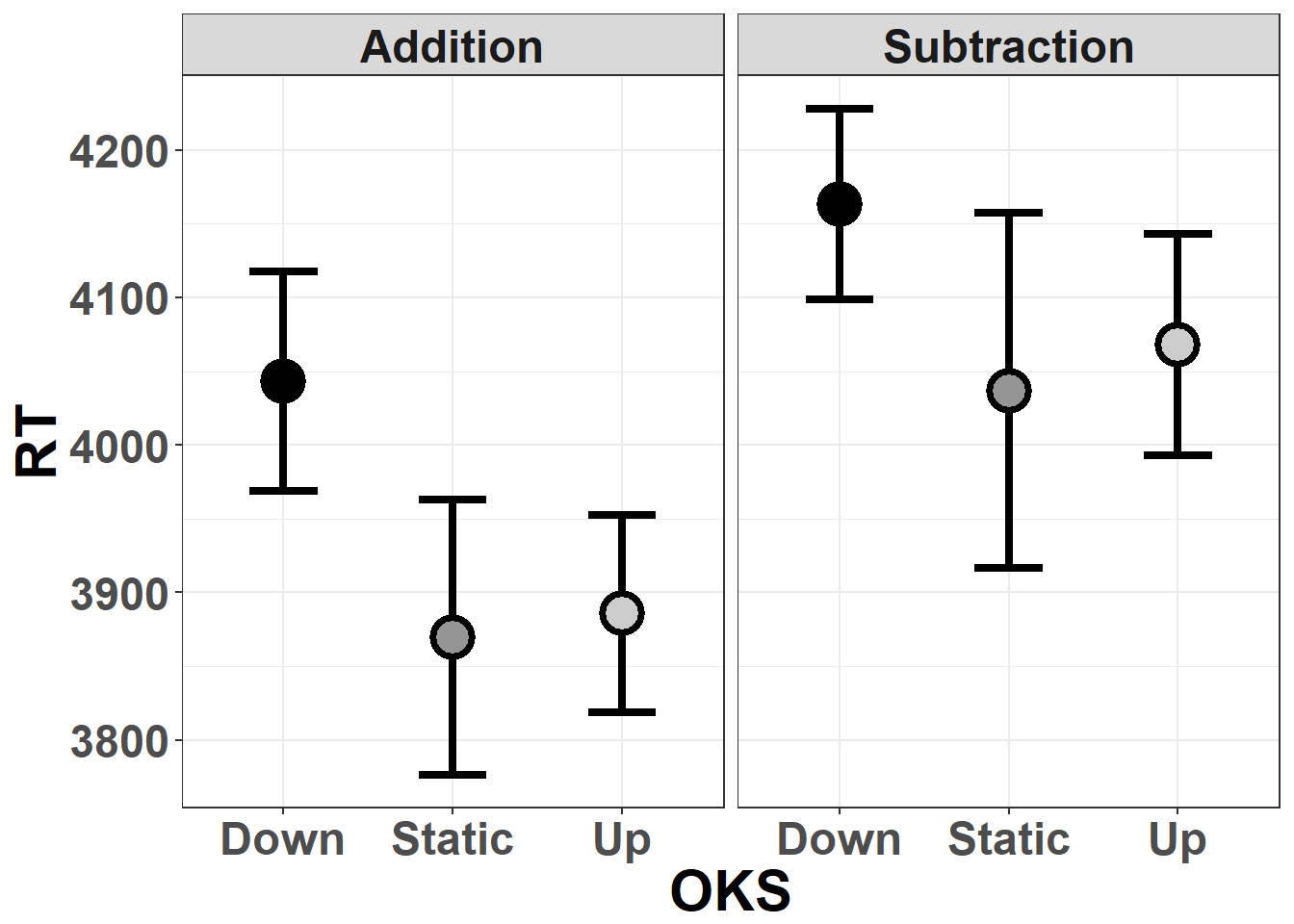

Supplement: Supplementary file 4 — Supplementary material 4 Fig. S4 Response times (RTs) for Exp 2 are depicted as a function of Operation Type and OKS. There were no effects of OKS, neither alone or in interaction with Operation type; Operation type, however, was significant and indicated faster responses for additions with respect to subtractions. Note, however, that RTs were calculated only for correct answers (and thus on the basis of very few trials) and three participants had to be discarded because they did not present enough observations. Error bars represent within-subjects SEM (Morey, 2008) (PNG 21 KB) [file 426_2018_1053_MOESM4_ESM.png]

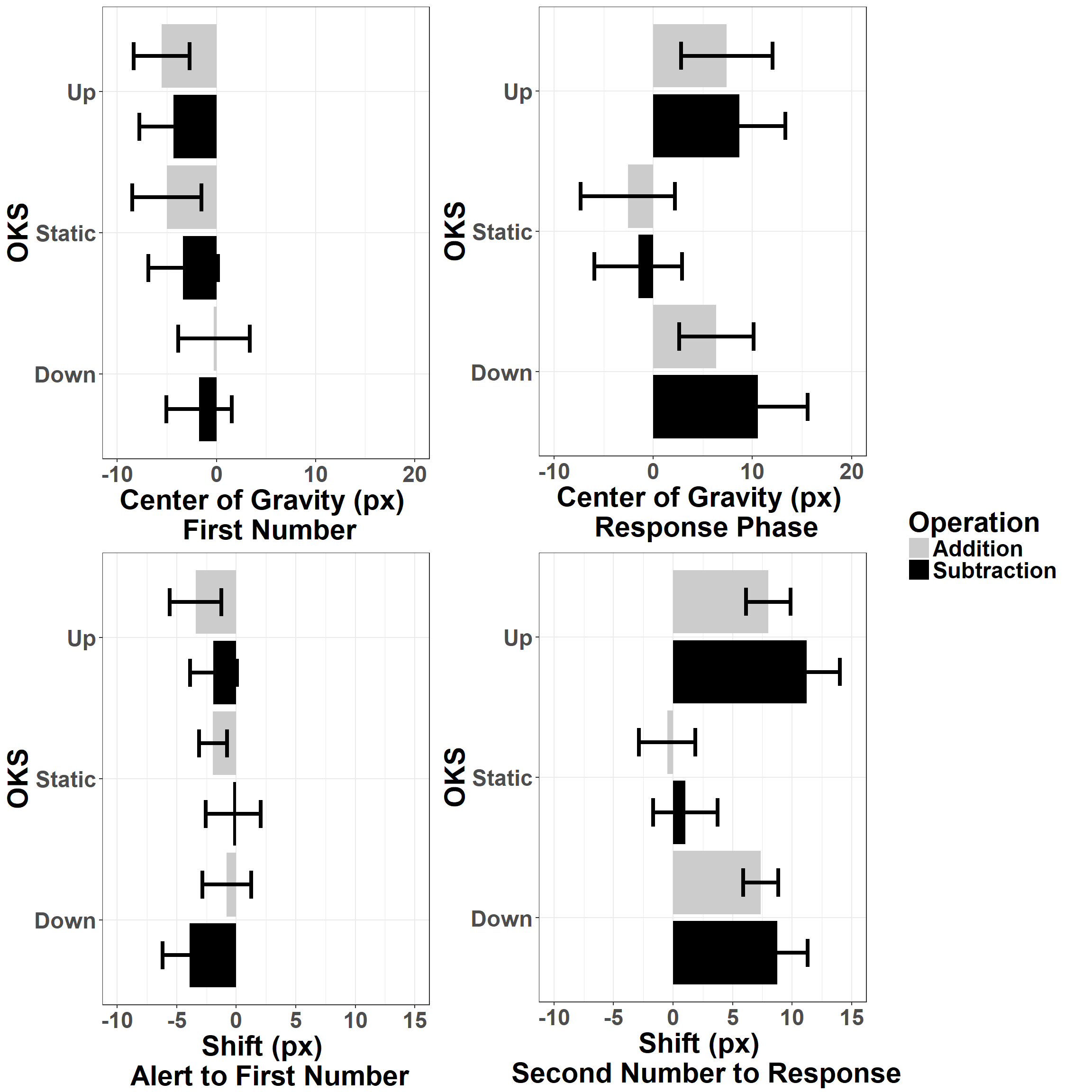

Supplement: Supplementary file 5 — Supplementary material 5 Fig. S5 Pattern of eye movements along the horizontal axis. Top panels: mean position of the eyes (in pixels with respect to the center) during the presentation of the first operand and during the response phase. Bottom panels: mean shift of gaze position in the same phases with respect to the preceding ones. There was no effect of Operation type in modulating oculomotor behaviour along the horizontal axis, unlike in experiment 1. Error bars represent within-subjects SEM (Morey, 2008) (PNG 81 KB) [file 426_2018_1053_MOESM5_ESM.png]
