# Supplementary material for: Spatial grounding of symbolic arithmetic: an investigation with optokinetic stimulation
Source: Psychol Res. 2018 Jul 18;83(1):64–83. doi: 10.1007/s00426-018-1053-0 (PMC6373542; doi:10.1007/s00426-018-1053-0)
Supplement: Supplementary file 6 — Supplementary material 6 (HTML 2378 KB) [file 426_2018_1053_MOESM6_ESM.html]

Spatial grounding of mental arithmetic - Exp 1, v0


# Spatial grounding of mental arithmetic - Exp 1, v0

#### *Elvio Blini INSERM U1028, ImpAct team, CRNL, and University of Lyon elvio.blini@gmail.com*

#### *10 May 2017*

# Experiment 1

# Behavioural data

This document includes data and analyses for the first experiment described in the companion paper.

We administered horizontal OKS while participants were cuncurrently asked to perform mental arithmetic.

We focus on the distribution of responses, isolating estimation and decade errors, when assessing behavioural performance.

For eye-tracking data, we focus on the center of gravity of gaze during different phases of calculation, and the displacement occurring between two consecutive phases.

I am very very sorry for variables having Italian names… Early career scripts in E-Prime… I’ll provide relevant translations in the text.

For any request or inquiry don’t hesitate contacting: elvio.blini@gmail.com

## Preliminary setup

It’s sometimes good to clean the current environment to avoid conflicts. You can do it with `rm(list=ls())` (but be sure everything is properly saved for future use).

In order to run this script we need a few packages available on CRAN. You might need to install them first, e.g. by typing `install.packages("BayesFactor")` in the console.

```
#list packages
packages= c("plyr", "BayesFactor", "reshape", "gridExtra", "ez", "tidyverse")

#load them
lapply(packages, require, character.only= T)
```

Thanks to the function retrieved here, not displayed, the following hyperlink downloads the Rdata file:

That can be loaded then with:

```
load("Exp 1 data.RData")
```

Now all relevant variables are stored in the `data` data.frame, that you can navigate and explore with the usual commands, e.g. `str(data)`.

## Useful functions

This is a convenient (minimal) list of ggplot attributes shared by different plots.

```
#ggplot defaults
commonTheme = list(theme_bw(),
                   theme(text= element_text(size=22, face="bold")),
                   scale_fill_grey(start = 0, end = .8),
                   guides(fill= F))
```

The package ez, and the function ezANOVA, does not return partial eta square by default. So, here it is:

```
extract.pes= function(EZ){
  return(
    cbind(Effects= EZ$ANOVA$Effect, pes= EZ$ANOVA$SSn/(EZ$ANOVA$SSn + EZ$ANOVA$SSd)))
}
```

Then (NOT SHOWN in the html file but shown in the appendix) we have a convenient summarising function that produces means and within subjects SEM as in Morey (2008). I retrieved it here: link

## Preprocessing

The factors OKS (SOK) and Deviant eye movement (Mosso) are to be converted into factors.

Response (Risposta.RESP) and Correct response (Risposta.CRESP) are numeric instead (NAs from coercion are expected).

```
#declare
data$SOK<-factor(data$SOK)
data$Mosso<-factor(data$Mosso)

data$Risposta.RESP<-as.numeric(as.character(data$Risposta.RESP))
data$Risposta.CRESP<-as.numeric(as.character(data$Risposta.CRESP))
```

We create an Accuracy (Accuratezza) variable and a convenience variable storing only “1” (Uno).

```
data$Accuratezza<-ifelse(data$Risposta.RESP == data$Risposta.CRESP, 1, 0)
data$Uno<-seq(1,1,length=nrow(data))
```

We drop practice trials.

```
 data<-data[data$Procedure=="TrialProc",]
```

I rename levels of Sign (Segno) as to be intelligible!

```
if(sum(levels(data$Segno)==c("meno", "piu"))==2)(
levels(data$Segno)= c("Subtraction", "Addition")) else(stop("Unordered levels"))
```

```
## [1] "Subtraction" "Addition"
```

```
#now reorder levels
data$Segno= relevel(data$Segno, "Addition")
```

Same for OKS (SOK).

```
data$SOK= factor(data$SOK, levels=c("sinistra", "staticaO", "destra"), 
                 labels=c("Left", "Static", "Right"))
```

Trials in which participants refused categorically to respond (dropped, return percentage dropped).

```
#subj refuses to respond
numtrial1<-sum(data$Uno)
data<-data[!is.na(data$Risposta.RESP),]
numtrial2<-sum(data$Uno)

rispostenonfornite<-((numtrial2-numtrial1)/numtrial1)*100
rispostenonfornite
```

```
## [1] -0.1844168
```

So we were scary enough to force them!

### Accuracy

We process and summarise accuracy:

```
ACC= data %>% 
  filter(Mosso== 0) %>% #invalid OKS
  ddply(c("Segno", "SOK", "Subject"), summarise, N= length(SecondoO.RT), ACC= mean(Accuratezza), .drop=F)
```

Overall, mean accuracy was:

```
ACC %>% with(tapply(ACC, list(Segno, SOK), mean))
```

```
##                  Left    Static     Right
## Addition    0.4354493 0.4704568 0.4849318
## Subtraction 0.2994411 0.3340682 0.2976223
```

```
ACC %>% with(tapply(ACC, list(Segno, SOK), sd))
```

```
##                  Left    Static     Right
## Addition    0.2471245 0.2488949 0.2425046
## Subtraction 0.2442596 0.2543708 0.1937434
```

### RTs

We process and summarise reaction times:

```
RTs= data %>% 
  mutate(SecondoO.RT= as.numeric(as.character(SecondoO.RT))) %>% #is actually a number, not a factor
  filter(Mosso== 0) %>% #invalid OKS
  filter(SecondoO.RT> 500) %>% #exclude anticipations
  filter(Accuratezza== 1) %>% #only accurate responses
  ddply(c("Segno", "SOK", "Subject"), summarise, N= length(SecondoO.RT), RT= mean(SecondoO.RT), .drop=F) %>%
  filter(!Subject %in% c(2, 21)) #not enough trials per cell
```

Overall, RTs are:

```
RTs %>% with(tapply(RT, list(Segno, SOK), mean))
```

```
##                 Left   Static    Right
## Addition    3628.256 3661.031 3582.643
## Subtraction 3625.457 3776.048 3561.872
```

```
RTs %>% with(tapply(RT, list(Segno, SOK), sd))
```

```
##                  Left   Static    Right
## Addition     978.7219 915.7327 908.8275
## Subtraction 1051.2374 980.5267 838.3557
```

### Errors distribution

I create a variable for the shift from the correct response.

```
#pure shift measure
data$Shift<-data$Risposta.RESP-data$Risposta.CRESP
```

And assess its distribution.

```
ggplot(data, aes(x= Shift)) + geom_density() + facet_wrap("Segno") + 
  commonTheme + scale_x_continuous(breaks= seq(-30, 30, 10), limits = c(-30, 30))
```

```
data$Error_type= with(data, ifelse(Shift== 0, "Correct Response", 
                                   ifelse(abs(Shift)== 10 | abs(Shift)== 20 | abs(Shift)== 30 | abs(Shift)== 40,  "Decade Error",
                                          "Estimation Error")))

data %>% ddply("Subject", count, Error_type) %>%
  ddply("Subject", mutate, percentage= n/sum(n)) %>%
  ddply("Error_type", summarise, Error_Type= mean(percentage)) %>%
  ggplot(aes(x= "", y= Error_Type, fill= Error_type)) + 
  xlab(NULL) + ylab(NULL) + labs(fill="Error Type") +
  geom_bar(width = 1, stat = "identity") +
  coord_polar("y", start= 0) + commonTheme
```

There are multiple peaks in correspondence of decades. Exclude deviant eye movements (Mosso==1), return percentage of trials dropped.

```
#exclude eye movements
data<-data[! is.na (data$Shift),]
A<-sum(data$Uno)
data<-data[data$Mosso=="0",]
B<-sum(data$Uno)
Escl.mossi.shift<-((A-B)/A)*100
Escl.mossi.shift
```

```
## [1] 3.163972
```

## Analyses

### Accuracy

```
(EZ.acc=ezANOVA(data= ACC, dv = .(ACC), within = .("SOK","Segno"), 
        type = 3, detailed = T, wid= .(Subject)))
```

```
## $ANOVA
##        Effect DFn DFd         SSn       SSd         F            p p<.05
## 1 (Intercept)   1  23 21.56616926 6.5421950 75.818879 9.726761e-09     *
## 2         SOK   2  46  0.03041403 0.6258252  1.117761 3.357296e-01      
## 3       Segno   1  23  0.84531999 0.3759979 51.708688 2.540552e-07     *
## 4   SOK:Segno   2  46  0.02089969 0.3618055  1.328595 2.748202e-01      
##           ges
## 1 0.731751305
## 2 0.003832299
## 3 0.096595374
## 4 0.002636612
## 
## $`Mauchly's Test for Sphericity`
##      Effect         W         p p<.05
## 2       SOK 0.9782569 0.7852034      
## 4 SOK:Segno 0.9279008 0.4390530      
## 
## $`Sphericity Corrections`
##      Effect       GGe     p[GG] p[GG]<.05      HFe     p[HF] p[HF]<.05
## 2       SOK 0.9787196 0.3349156           1.068752 0.3357296          
## 4 SOK:Segno 0.9327495 0.2744511           1.011899 0.2748202
```

We now plot results:

```
ACC %>% 
  summarySEwithin(measurevar = "ACC", withinvars = c("Segno", "SOK"), idvar = "Subject") %>%
  ggplot(aes(y= ACC, x= SOK, fill= SOK)) +  
  geom_errorbar(aes(ymin= ACC-se, ymax= ACC+se), width= .4, size= 1.5, colour= "black") +
  geom_point(shape= 21, size= 6, stroke= 2) + facet_wrap("Segno", ncol= 2) + xlab("OKS") + 
  scale_fill_discrete(guide=F) + ylab("Accuracy") +
  commonTheme
```

We only found a main effect of Operation type, Additions being more accurate than subtractions, but no OKS effects.

### RTs

```
(EZ.rts=ezANOVA(data= RTs, dv = .(RT), within = .("SOK","Segno"), 
        type = 3, detailed = T, wid= .(Subject)))
```

```
## $ANOVA
##        Effect DFn DFd          SSn      SSd           F            p p<.05
## 1 (Intercept)   1  21 1.748196e+09 99559481 368.7454772 8.447364e-15     *
## 2         SOK   2  42 4.808497e+05  5473268   1.8449387 1.706142e-01      
## 3       Segno   1  21 3.066232e+04  4150931   0.1551239 6.976568e-01      
## 4   SOK:Segno   2  42 1.196874e+05  4044196   0.6214920 5.420088e-01      
##            ges
## 1 0.9391713518
## 2 0.0042287850
## 3 0.0002707285
## 4 0.0010559328
## 
## $`Mauchly's Test for Sphericity`
##      Effect         W         p p<.05
## 2       SOK 0.9269414 0.4682984      
## 4 SOK:Segno 0.9576884 0.6489967      
## 
## $`Sphericity Corrections`
##      Effect       GGe     p[GG] p[GG]<.05      HFe     p[HF] p[HF]<.05
## 2       SOK 0.9319155 0.1736792           1.019125 0.1706142          
## 4 SOK:Segno 0.9594060 0.5357780           1.053757 0.5420088
```

We now plot results:

```
RTs %>% 
  summarySEwithin(measurevar = "RT", withinvars = c("Segno", "SOK"), idvar = "Subject") %>%
  ggplot(aes(y= RT, x= SOK, fill= SOK)) +  
  geom_errorbar(aes(ymin= RT-se, ymax= RT+se), width= .4, size= 1.5, colour= "black") +
  geom_point(shape= 21, size= 6, stroke= 2) + facet_wrap("Segno", ncol= 2) + xlab("OKS") + ylab("RTs (ms)")+ 
  scale_fill_discrete(guide=F) +
  commonTheme
```

Nothing was found to be significant here, instead.

### Displacement - estimation errors

We can now prepare data frames for ANOVA purposes.

```
X<- data #safe version of "data" 
X$Subject=as.factor(X$Subject)
```

We identify positive & negative decades errors, then exclude them to isolate estimations.

```
X$PDE<-ifelse(X$Shift==10 |X$Shift==20|X$Shift==30|X$Shift==40, 1, 0)
X$NDE<-ifelse(X$Shift==-10|X$Shift==-20|X$Shift==-30|X$Shift==-40, 1, 0)

Y=X[X$PDE==0 & X$NDE==0,]
1-nrow(Y)/nrow(X) #decade errors, proportion
```

```
## [1] 0.1094682
```

We summarise and run ANOVA.

```
#summarise
DFshift=ddply(Y, c("Subject","SOK","Segno"), summarise,
              Shift = mean(Shift))
DFshift$Subject=as.factor(DFshift$Subject)

(EZ1=ezANOVA(data=DFshift, dv = .(Shift), within = .("SOK","Segno"), 
        type = 3, detailed = T, wid= .(Subject)))
```

```
## $ANOVA
##        Effect DFn DFd         SSn       SSd         F           p p<.05
## 1 (Intercept)   1  23   0.5044603  87.03317 0.1333123 0.718358308      
## 2         SOK   2  46   2.3112158  92.99672 0.5716112 0.568574079      
## 3       Segno   1  23 153.7914729 391.62795 9.0320516 0.006310741     *
## 4   SOK:Segno   2  46   3.8991747 101.22975 0.8859157 0.419253458      
##            ges
## 1 0.0007491332
## 2 0.0034230152
## 3 0.1860352819
## 4 0.0057613047
## 
## $`Mauchly's Test for Sphericity`
##      Effect         W          p p<.05
## 2       SOK 0.9626612 0.65797329      
## 4 SOK:Segno 0.7989837 0.08470661      
## 
## $`Sphericity Corrections`
##      Effect       GGe     p[GG] p[GG]<.05       HFe     p[HF] p[HF]<.05
## 2       SOK 0.9640052 0.5625830           1.0505000 0.5685741          
## 4 SOK:Segno 0.8326282 0.4033462           0.8897729 0.4091687
```

Only a main effect of Sign (thus, operation type). Let’s have partial eta squared:

```
extract.pes(EZ1)
```

```
##      Effects       pes                 
## [1,] "(Intercept)" "0.0057627826522204"
## [2,] "SOK"         "0.0242499826373588"
## [3,] "Segno"       "0.281969187657516" 
## [4,] "SOK:Segno"   "0.0370894578965539"
```

A Bayesian counterpart:

```
BF1= anovaBF(formula = Shift ~ SOK*Segno + Subject, data = DFshift,whichModels = "all", whichRandom = "Subject")
head(BF1)
```

When compared against the model 

...

Enter one or more search terms in the box to filter the models in the table. If more than one term is included, matching will be performed with `or`. Special search terms are allowed:

| Code | Function | Example | What example does |
| --- | --- | --- | --- |
| + | Require this term in search results | +shape | Requires all models to include âshapeâ |
| - | Require this term NOT to appear in search results | -shape | Requires all models to exclude âshapeâ |
| # (with number) | Return results with certain number of terms | #2 | Requires all models to have two terms |
| @ (with :, ::, â¦) | Return results containing interactions of certain size | @:: | Requires all models to have a three-way interaction |
| < or > | Limits sizes of Bayes factors | >2 | Returns models whose Bayes factor is greater than 2 |

Click on a row in the Bayes factor table to make that model the denominator. Sort by clicking on the arrows in the column headers.

|  |  |  |
| --- | --- | --- |
| ...the model below... | ...is preferred by... |  |

Main effect of Operation type confirmed. We can now plot the results (first summarised with `summarySE`).

```
#summarise then plot shift
DF= summarySEwithin(data = DFshift, measurevar = "Shift", withinvars = c("Segno", "SOK"),
                    idvar = "Subject")

ggplot(DF, aes(y= Shift, x= SOK, fill= SOK)) + geom_bar(position= "dodge", stat= "identity") + 
  facet_wrap("Segno", nrow= 2) + xlab("OKS") + scale_fill_discrete(guide=F) + ylim(c(-2, 3)) +
  geom_errorbar(aes(ymin= Shift-se, ymax= Shift+se), width= .4, size= 1.5, colour= "black") +
  coord_flip() + commonTheme
```

## Positive decade errors

I’m summarising asking for the proportion (mean, since this values is binomial) of positive errors. Then we use asin transform on data.

```
positive=ddply(X, c("Subject","SOK","Segno"), summarise,
               PDE = mean(PDE))
positive$asinPDE=asin(sqrt(positive$PDE))
```

Now anova and partial eta square.

```
(EZ2= ezANOVA(data=positive, dv = .(asinPDE), within = .("SOK","Segno"), 
        type = 3, detailed = T, wid= .(Subject)))
```

```
## $ANOVA
##        Effect DFn DFd          SSn       SSd            F            p
## 1 (Intercept)   1  23 3.9243580077 0.8043154 112.21995064 2.548282e-10
## 2         SOK   2  46 0.0307935139 0.4974338   1.42380909 2.512072e-01
## 3       Segno   1  23 0.3021429730 0.8115359   8.56313128 7.592878e-03
## 4   SOK:Segno   2  46 0.0006681772 0.4362939   0.03522414 9.654150e-01
##   p<.05          ges
## 1     * 0.6061779695
## 2       0.0119337473
## 3     * 0.1059510614
## 4       0.0002620049
## 
## $`Mauchly's Test for Sphericity`
##      Effect         W         p p<.05
## 2       SOK 0.9816977 0.8161224      
## 4 SOK:Segno 0.9388602 0.4995857      
## 
## $`Sphericity Corrections`
##      Effect       GGe     p[GG] p[GG]<.05      HFe     p[HF] p[HF]<.05
## 2       SOK 0.9820266 0.2513576           1.072861 0.2512072          
## 4 SOK:Segno 0.9423829 0.9593954           1.023772 0.9654150
```

```
extract.pes(EZ2)
```

```
##      Effects       pes                  
## [1,] "(Intercept)" "0.829906756426206"  
## [2,] "SOK"         "0.0582959473749748" 
## [3,] "Segno"       "0.271301703367031"  
## [4,] "SOK:Segno"   "0.00152914232350002"
```

Again, only Operation type (Segno). Note that this does not change at all when using non-asin transformed values (not shown). Confirmed by Bayesian counterpart:

```
BF2= anovaBF(formula = asinPDE ~ SOK*Segno + Subject, data = positive,whichModels = "all", whichRandom = "Subject" )
head(BF2)
```

When compared against the model 

...

Enter one or more search terms in the box to filter the models in the table. If more than one term is included, matching will be performed with `or`. Special search terms are allowed:

| Code | Function | Example | What example does |
| --- | --- | --- | --- |
| + | Require this term in search results | +shape | Requires all models to include âshapeâ |
| - | Require this term NOT to appear in search results | -shape | Requires all models to exclude âshapeâ |
| # (with number) | Return results with certain number of terms | #2 | Requires all models to have two terms |
| @ (with :, ::, â¦) | Return results containing interactions of certain size | @:: | Requires all models to have a three-way interaction |
| < or > | Limits sizes of Bayes factors | >2 | Returns models whose Bayes factor is greater than 2 |

Click on a row in the Bayes factor table to make that model the denominator. Sort by clicking on the arrows in the column headers.

|  |  |  |
| --- | --- | --- |
| ...the model below... | ...is preferred by... |  |

## Negative decade errors

I’m summarising asking for the proportion (mean, since this values is binomial) of negative errors. Then we use asin transform on data.

```
negative=ddply(X, c("Subject","SOK","Segno"), summarise,
               NDE = mean(NDE))
negative$asinNDE=asin(sqrt(negative$NDE))
```

Now anova and partial eta square.

```
(EZ3= ezANOVA(data=negative, dv = .(asinNDE), within = .("SOK","Segno"), 
        type = 3, detailed = T, wid= .(Subject)))
```

```
## $ANOVA
##        Effect DFn DFd         SSn       SSd           F            p p<.05
## 1 (Intercept)   1  23 5.991620257 1.2700231 108.5076823 3.519014e-10     *
## 2         SOK   2  46 0.053786405 0.6411828   1.9293833 1.568104e-01      
## 3       Segno   1  23 0.369194332 0.7101258  11.9576970 2.135689e-03     *
## 4   SOK:Segno   2  46 0.004299244 0.6927206   0.1427453 8.673574e-01      
##           ges
## 1 0.643867514
## 2 0.015970600
## 3 0.100236115
## 4 0.001295596
## 
## $`Mauchly's Test for Sphericity`
##      Effect         W         p p<.05
## 2       SOK 0.8546443 0.1776804      
## 4 SOK:Segno 0.9363304 0.4849762      
## 
## $`Sphericity Corrections`
##      Effect       GGe     p[GG] p[GG]<.05      HFe     p[HF] p[HF]<.05
## 2       SOK 0.8730912 0.1630496           0.938852 0.1598305          
## 4 SOK:Segno 0.9401416 0.8551773           1.021008 0.8673574
```

```
extract.pes(EZ3)
```

```
##      Effects       pes                 
## [1,] "(Intercept)" "0.825105274525697" 
## [2,] "SOK"         "0.0773939451232607"
## [3,] "Segno"       "0.342061921278811" 
## [4,] "SOK:Segno"   "0.0061680363397956"
```

Again, only Operation type (Segno). Note that this does not change at all when using non-asin transformed values (not shown). Confirmed by Bayesian counterpart:

```
BF3= anovaBF(formula = asinNDE ~ SOK*Segno + Subject, data = negative,whichModels = "all", whichRandom = "Subject" )
head(BF3)
```

When compared against the model 

...

Enter one or more search terms in the box to filter the models in the table. If more than one term is included, matching will be performed with `or`. Special search terms are allowed:

| Code | Function | Example | What example does |
| --- | --- | --- | --- |
| + | Require this term in search results | +shape | Requires all models to include âshapeâ |
| - | Require this term NOT to appear in search results | -shape | Requires all models to exclude âshapeâ |
| # (with number) | Return results with certain number of terms | #2 | Requires all models to have two terms |
| @ (with :, ::, â¦) | Return results containing interactions of certain size | @:: | Requires all models to have a three-way interaction |
| < or > | Limits sizes of Bayes factors | >2 | Returns models whose Bayes factor is greater than 2 |

Click on a row in the Bayes factor table to make that model the denominator. Sort by clicking on the arrows in the column headers.

|  |  |  |
| --- | --- | --- |
| ...the model below... | ...is preferred by... |  |

## Plot decade errors

Switch to percentage.

```
positive$PDE= positive$PDE*100
negative$NDE= negative$NDE*100
```

Distribution/skewness:

```
positive %>% ddply(c("Subject"), summarise, PDE= mean(PDE)) %>% 
  ggplot(aes(x= PDE)) + 
  geom_histogram(bins= 10, fill=I("blue"), col=I("red"), alpha=I(.2), size= 1.2) + 
  commonTheme
```

```
negative %>% ddply(c("Subject"), summarise, NDE= mean(NDE)) %>% 
  ggplot(aes(x= NDE)) + 
  geom_histogram(bins= 10, fill=I("blue"), col=I("red"), alpha=I(.2), size= 1.2) + 
  commonTheme
```

Negative errors (summarise + plot):

```
DFn= summarySEwithin(data = negative, measurevar = "NDE", withinvars = c("Segno", "SOK"),
                     idvar = "Subject")

n= ggplot(DFn, aes(y= NDE, x= SOK, fill= SOK)) + geom_bar(position= "dodge", stat= "identity") + 
  facet_wrap("Segno", ncol= 2) + xlab("OKS") + scale_fill_discrete(guide=F) + ylim(c(0, 12.5)) +
  geom_errorbar(aes(ymin= NDE-se, ymax= NDE+se), width= .4, size= 1.5, colour= "black") +
  ylab("Percent of Negative Decade Errors (%)") + commonTheme
```

Now same for positive:

```
DFp= summarySEwithin(data = positive, measurevar = "PDE", withinvars = c("Segno", "SOK"),
                     idvar = "Subject")

p= ggplot(DFp, aes(y= PDE, x= SOK, fill= SOK)) + geom_bar(position= "dodge", stat= "identity") + 
  facet_wrap("Segno", ncol= 2) + xlab("OKS") + scale_fill_discrete(guide=F) + ylim(c(0, 12.5)) +
  geom_errorbar(aes(ymin= PDE-se, ymax= PDE+se), width= .4, size= 1.5, colour= "black") +
  ylab("Percent of Positive Decade Errors (%)") + commonTheme
```

Arrange the two plots:

```
grid.arrange(p, n, ncol=2)
```

# Eye-tracking data

I suggest to run `rm(list=ls())` again. We will need to load behavioural data again (we need the rawest form possible) and then eye movements data. For behavioural data:

```
load("Exp 1 data.RData")
```

You can download eye tracking data here:

That can be loaded then with:

```
load("Exp 1 data eye tracking.RData")
```

Now all relevant eye tracking variables are stored in the `MOC` data.frame, that you can navigate and explore with the usual commands, e.g. `str(MOC)`. Please note that eye tracking data have been summarised already for each subject and trial because each individual raw file weights up to >60-70 MB…

## Preprocessing

For behavioural data similar as above.

```
#declare
data$SOK<-factor(data$SOK)
data$Mosso<-factor(data$Mosso)

data$Risposta.RESP<-as.numeric(as.character(data$Risposta.RESP))
data$Risposta.CRESP<-as.numeric(as.character(data$Risposta.CRESP))

data$Accuratezza<-ifelse(data$Risposta.RESP == data$Risposta.CRESP, 1, 0)
data$Shift<-data$Risposta.RESP-data$Risposta.CRESP

data$Uno<-seq(1,1,length=nrow(data))

#exclude practice
data<-data[data$Procedure=="TrialProc",]
```

I miss a variable indicating the trial number:

```
PONTE<-{}
for (i in 1:max(as.numeric(as.character(data$Subject)))) {
  TEMP<-data[data$Subject== i,]
  
  TEMP$TrialN<-seq(1:max(nrow(TEMP)))
  PONTE<-rbind(PONTE, TEMP)
}
data<-PONTE
```

We need to retain only trials for which we also have eye tracking data (thus, no eye tracking failures of some sort).

```
PONTE<-{}
for (i in 1:max(as.numeric(as.character(data$Subject)))) {
  TEMPED<-data[data$Subject== i,]
  TEMPMOC<-MOC[MOC$Subject== i,]
  
  TEMPED<-TEMPED[TEMPMOC$NumTrial,]
  PONTE<-rbind(PONTE, TEMPED)
}
data<-PONTE
```

We can now start working on the MOC dataframe. First all variables indicating the center of gravity must be declared numeric.

```
names(MOC)
```

```
##  [1] "NumTrial"     "MeanAX"       "MeanN1X"      "MeanSX"      
##  [5] "MeanN2X"      "MeanRX"       "MeanAY"       "MeanN1Y"     
##  [9] "MeanSY"       "MeanN2Y"      "MeanRY"       "ShiftNRX"    
## [13] "ShiftNRY"     "SumDifRX"     "SumDifRY"     "ReacTim"     
## [17] "Mosso"        "Corretta"     "Segno"        "SOK"         
## [21] "SecondoORESP" "Grand"        "Uno"          "RispostaRESP"
## [25] "Shift"        "Subject"
```

```
for(i in 1:(ncol(MOC)-11)) {
  MOC[,i] <- as.numeric(as.character(MOC[,i]))
}
```

Values refers to gaze on the x and y axis. We want to code it with respect to center of the screen, so that the center of gravity refers to the geometrical coordinates of it. We thus subtract half the horizontal screen resolution from x and y, and we additionally flip y (because 0 was coded as the upper corner of the screen which is quite confusing to me).

```
for(i in 2:6) {
  MOC[,i] <- (MOC[,i]) - 512
}

for(i in 7:11) {
  MOC[,i] <- (((MOC[,i]) - 768) * (-1))-384
}

#shiftY only has to be flipped
MOC$ShiftNRY<-(-1)*MOC$ShiftNRY
```

Renaming levels.

```
if(sum(levels(MOC$Segno)==c("meno", "piu"))==2)(
  levels(MOC$Segno)= c("Subtraction", "Addition")) else(stop("Unordered levels"))
```

```
## [1] "Subtraction" "Addition"
```

```
  MOC$Segno= relevel(MOC$Segno, "Addition")
  
  MOC$SOK= factor(MOC$SOK, levels=c("sinistra", "staticaO", "destra"), 
                   labels=c("Left", "Static", "Right"))

#rename variables
colnames(MOC)[colnames(MOC)=="SOK"]= "OKS"  
colnames(MOC)[colnames(MOC)=="Segno"]= "Type"
```

## Summarise

A custom function that returns a tapply object for a given parameter as a function of OKS and Operation type, by subject, and print necessary omissions in computing it (i.e. absence of samples).

```
my_Summary= function(DF= MOC, dv){
  conta<-sum(DF$Uno)
  SenzaNA<-DF[! is.na(DF[,dv]),]
  conta2<-sum(SenzaNA$Uno)
  perc.escl<- ((conta2-conta)/conta)*100
  print(perc.escl)
  MeanSOGG<-tapply(na.omit(SenzaNA[, dv]), list(SenzaNA$Subject, SenzaNA$OKS,SenzaNA$Type),mean)
  return(MeanSOGG)
}
```

It can be used for any index. Here we focus on those reported in the paper.

```
#center of gravity, first number (x and y)
MeanN1X= my_Summary(dv= "MeanN1X")
```

```
## [1] -1.133284
```

```
MeanN1Y= my_Summary(dv= "MeanN1Y")
```

```
## [1] -1.133284
```

```
#printed values, omissions, are the same because of course samples x and y are coupled

#center of gravity, first number (x and y)
MeanRX= my_Summary(dv= "MeanRX")
```

```
## [1] 0
```

```
MeanRY= my_Summary(dv= "MeanRY")
```

```
## [1] 0
```

```
#here values are zero because trials without samples for this last phase were dismissed already in the preprocessing phase
```

Same for shift values (bur shift from alert to first number is not yet in the dataframe).

```
MOC$ShiftN1AX<-MOC$MeanN1X-MOC$MeanAX
MOC$ShiftN1AY<-MOC$MeanN1Y-MOC$MeanAY

#shift, alert to first number (x and y)
ShiftN1AX= my_Summary(dv= "ShiftN1AX")
```

```
## [1] -2.68539
```

```
ShiftN1AY= my_Summary(dv= "ShiftN1AY")
```

```
## [1] -2.68539
```

```
#shift, second number to response (x and y)
ShiftNRX= my_Summary(dv= "ShiftNRX")
```

```
## [1] -1.946292
```

```
ShiftNRY= my_Summary(dv= "ShiftNRY")
```

```
## [1] -1.946292
```

## Analyses

Another function to ease analyses. It takes one of the tapply objects created before and returns frequentist anova (plus partial eta square) and Bayesian one.

```
my_analysis= function(X){
  
  X= melt(X)
  colnames(X)= c("Subject", "OKS", "Type", "DV")
  
  X$Subject= as.factor(X$Subject)
 
  EZ= ezANOVA(data=X, dv = .(DV), within = .("OKS","Type"), 
          type = 3, detailed = T, wid= .(Subject))
  print(EZ)
  print(extract.pes(EZ))
  BF= anovaBF(formula = DV ~ OKS*Type + Subject, data = X,
          whichModels = "all", whichRandom = "Subject")
  print(head(BF))
  
}
```

Here we go:

```
my_analysis(MeanN1X)
```

```
## $ANOVA
##        Effect DFn DFd         SSn        SSd          F            p p<.05
## 1 (Intercept)   1  23   7000.7517 155096.096  1.0381776 3.188445e-01      
## 2         OKS   2  46 287706.4144 270568.470 24.4568317 5.819279e-08     *
## 3        Type   1  23    114.6525   1365.692  1.9308941 1.779667e-01      
## 4    OKS:Type   2  46    195.5500   5873.217  0.7657897 4.708022e-01      
##            ges
## 1 0.0159142634
## 2 0.3992540462
## 3 0.0002647752
## 4 0.0004515133
## 
## $`Mauchly's Test for Sphericity`
##     Effect         W            p p<.05
## 2      OKS 0.5296654 0.0009204879     *
## 4 OKS:Type 0.5801836 0.0025073804     *
## 
## $`Sphericity Corrections`
##     Effect       GGe        p[GG] p[GG]<.05       HFe        p[HF]
## 2      OKS 0.6801173 4.516650e-06         * 0.7080860 3.082307e-06
## 4 OKS:Type 0.7043164 4.305005e-01           0.7365719 4.356780e-01
##   p[HF]<.05
## 2         *
## 4          
## 
##      Effects       pes                 
## [1,] "(Intercept)" "0.0431886975645266"
## [2,] "OKS"         "0.515349019541689" 
## [3,] "Type"        "0.0774498530384701"
## [4,] "OKS:Type"    "0.0322223527731965"
## Bayes factor analysis
## --------------
## [1] OKS + Subject                   : 3.498083e+16 ±0.71%
## [2] OKS + Type + Subject            : 6.310522e+15 ±1.54%
## [3] OKS + OKS:Type + Subject        : 4.138147e+15 ±1%
## [4] OKS + Type + OKS:Type + Subject : 7.650861e+14 ±2.19%
## [5] Type + Subject                  : 0.1865804    ±2.67%
## [6] OKS:Type + Subject              : 0.1185171    ±1.22%
## 
## Against denominator:
##   DV ~ Subject 
## ---
## Bayes factor type: BFlinearModel, JZS
```

```
my_analysis(MeanRX)
```

```
## $ANOVA
##        Effect DFn DFd        SSn        SSd         F           p p<.05
## 1 (Intercept)   1  23  1471.6917 221171.411 0.1530438 0.699244044      
## 2         OKS   2  46 94085.9985 281112.685 7.6979022 0.001306893     *
## 3        Type   1  23   637.1169   3997.106 3.6660752 0.068050260      
## 4    OKS:Type   2  46   334.6264   3831.071 2.0089441 0.145729356      
##            ges
## 1 0.0028767355
## 2 0.1557204036
## 3 0.0012474159
## 4 0.0006555558
## 
## $`Mauchly's Test for Sphericity`
##     Effect         W          p p<.05
## 2      OKS 0.7725004 0.05846368      
## 4 OKS:Type 0.7637953 0.05161157      
## 
## $`Sphericity Corrections`
##     Effect       GGe       p[GG] p[GG]<.05       HFe       p[HF] p[HF]<.05
## 2      OKS 0.8146642 0.002842763         * 0.8681029 0.002270665         *
## 4 OKS:Type 0.8089276 0.155818803           0.8611980 0.153077818          
## 
##      Effects       pes                  
## [1,] "(Intercept)" "0.00661009359281377"
## [2,] "OKS"         "0.25076313638999"   
## [3,] "Type"        "0.137480868598783"  
## [4,] "OKS:Type"    "0.0803290251058024" 
## Bayes factor analysis
## --------------
## [1] OKS + Subject                   : 255656.8  ±0.66%
## [2] OKS + Type + Subject            : 50834.8   ±1.24%
## [3] OKS + OKS:Type + Subject        : 31108.85  ±1.09%
## [4] OKS + Type + OKS:Type + Subject : 6213.145  ±2.11%
## [5] Type + Subject                  : 0.19225   ±0.87%
## [6] OKS:Type + Subject              : 0.1222406 ±0.71%
## 
## Against denominator:
##   DV ~ Subject 
## ---
## Bayes factor type: BFlinearModel, JZS
```

```
my_analysis(ShiftN1AX)
```

```
## $ANOVA
##        Effect DFn DFd       SSn      SSd         F         p p<.05
## 1 (Intercept)   1  23 441.78351 4594.922 2.2113585 0.1505796      
## 2         OKS   2  46 293.71133 8490.607 0.7956275 0.4574098      
## 3        Type   1  23  29.17423 1561.638 0.4296818 0.5186487      
## 4    OKS:Type   2  46  72.95898 5787.316 0.2899542 0.7496553      
##           ges
## 1 0.021161998
## 2 0.014169654
## 3 0.001425661
## 4 0.003557683
## 
## $`Mauchly's Test for Sphericity`
##     Effect         W            p p<.05
## 2      OKS 0.6145957 4.725899e-03     *
## 4 OKS:Type 0.2839210 9.664338e-07     *
## 
## $`Sphericity Corrections`
##     Effect       GGe     p[GG] p[GG]<.05       HFe     p[HF] p[HF]<.05
## 2      OKS 0.7218109 0.4222024           0.7572451 0.4274238          
## 4 OKS:Type 0.5827237 0.6298429           0.5947166 0.6344282          
## 
##      Effects       pes                 
## [1,] "(Intercept)" "0.087712787170609" 
## [2,] "OKS"         "0.0334358707118714"
## [3,] "Type"        "0.0183392058519465"
## [4,] "OKS:Type"    "0.0124497542517412"
## Bayes factor analysis
## --------------
## [1] Type + Subject            : 0.197974   ±1.49%
## [2] OKS + Subject             : 0.177461   ±0.6%
## [3] OKS:Type + Subject        : 0.1467741  ±1.35%
## [4] OKS + Type + Subject      : 0.03451117 ±1.28%
## [5] Type + OKS:Type + Subject : 0.02756931 ±1.16%
## [6] OKS + OKS:Type + Subject  : 0.026167   ±1.25%
## 
## Against denominator:
##   DV ~ Subject 
## ---
## Bayes factor type: BFlinearModel, JZS
```

```
my_analysis(ShiftNRX)
```

```
## $ANOVA
##        Effect DFn DFd       SSn       SSd         F           p p<.05
## 1 (Intercept)   1  23  2809.530 31878.440 2.0270499 0.167945901      
## 2         OKS   2  46 17930.529 57130.676 7.2185766 0.001876887     *
## 3        Type   1  23  1095.075  2687.363 9.3722804 0.005529925     *
## 4    OKS:Type   2  46   118.067  9094.277 0.2985989 0.743283909      
##           ges
## 1 0.027118940
## 2 0.151030447
## 3 0.010748057
## 4 0.001170036
## 
## $`Mauchly's Test for Sphericity`
##     Effect         W            p p<.05
## 2      OKS 0.7081473 0.0224573978     *
## 4 OKS:Type 0.5089017 0.0005928886     *
## 
## $`Sphericity Corrections`
##     Effect       GGe       p[GG] p[GG]<.05       HFe       p[HF] p[HF]<.05
## 2      OKS 0.7740820 0.004504656         * 0.8194156 0.003776296         *
## 4 OKS:Type 0.6706466 0.655505600           0.6969723 0.664082878          
## 
##      Effects       pes                 
## [1,] "(Intercept)" "0.0809943594230068"
## [2,] "OKS"         "0.2388787757145"   
## [3,] "Type"        "0.289515607014846" 
## [4,] "OKS:Type"    "0.0128161728193786"
## Bayes factor analysis
## --------------
## [1] OKS + Subject                   : 17520.45  ±0.8%
## [2] OKS + Type + Subject            : 7361.03   ±1.51%
## [3] OKS + OKS:Type + Subject        : 2220.32   ±1.33%
## [4] OKS + Type + OKS:Type + Subject : 998.6429  ±4.54%
## [5] Type + Subject                  : 0.3555879 ±0.81%
## [6] OKS:Type + Subject              : 0.1241783 ±0.78%
## 
## Against denominator:
##   DV ~ Subject 
## ---
## Bayes factor type: BFlinearModel, JZS
```

The effect of interest is Operation type. When exploring the horizontal plane we observe that it is significant for the shift occurring between the second number presentation and response, addition bringing rigtward shifts and subtraction leftward ones.

```
my_analysis(MeanN1Y)
```

```
## $ANOVA
##        Effect DFn DFd        SSn        SSd         F          p p<.05
## 1 (Intercept)   1  23 49454.9211 240349.888 4.7325305 0.04012686     *
## 2         OKS   2  46   663.0999  27494.255 0.5547085 0.57803261      
## 3        Type   1  23   134.8918   1052.019 2.9491019 0.09936302      
## 4    OKS:Type   2  46   114.2767   1176.363 2.2343131 0.11855940      
##            ges
## 1 0.1547751900
## 2 0.0024492523
## 3 0.0004992156
## 4 0.0004229544
## 
## $`Mauchly's Test for Sphericity`
##     Effect         W         p p<.05
## 2      OKS 0.8627398 0.1970962      
## 4 OKS:Type 0.8928509 0.2874538      
## 
## $`Sphericity Corrections`
##     Effect       GGe     p[GG] p[GG]<.05       HFe     p[HF] p[HF]<.05
## 2      OKS 0.8793063 0.5565226           0.9464236 0.5688065          
## 4 OKS:Type 0.9032207 0.1243833           0.9756406 0.1200086          
## 
##      Effects       pes                 
## [1,] "(Intercept)" "0.170649069916598" 
## [2,] "OKS"         "0.0235497928188631"
## [3,] "Type"        "0.113649479935616" 
## [4,] "OKS:Type"    "0.0885426570455254"
## Bayes factor analysis
## --------------
## [1] Type + Subject            : 0.2273755  ±1.8%
## [2] OKS + Subject             : 0.2016636  ±0.84%
## [3] OKS:Type + Subject        : 0.1386287  ±1.17%
## [4] OKS + Type + Subject      : 0.04676855 ±1.67%
## [5] Type + OKS:Type + Subject : 0.03111407 ±2.18%
## [6] OKS + OKS:Type + Subject  : 0.02778218 ±1.08%
## 
## Against denominator:
##   DV ~ Subject 
## ---
## Bayes factor type: BFlinearModel, JZS
```

```
my_analysis(MeanRY)
```

```
## $ANOVA
##        Effect DFn DFd        SSn        SSd          F            p p<.05
## 1 (Intercept)   1  23 59894.3652 258131.971  5.3366904 0.0301996432     *
## 2         OKS   2  46  1671.8590  40604.465  0.9470081 0.3953313108      
## 3        Type   1  23  1348.5347   1880.616 16.4926265 0.0004832884     *
## 4    OKS:Type   2  46   183.5933   2016.491  2.0940565 0.1347752625      
##            ges
## 1 0.1652131159
## 2 0.0054940170
## 3 0.0044362309
## 4 0.0006062843
## 
## $`Mauchly's Test for Sphericity`
##     Effect         W         p p<.05
## 2      OKS 0.9845742 0.8428167      
## 4 OKS:Type 0.8958954 0.2984217      
## 
## $`Sphericity Corrections`
##     Effect       GGe     p[GG] p[GG]<.05       HFe     p[HF] p[HF]<.05
## 2      OKS 0.9848086 0.3942301           1.0763192 0.3953313          
## 4 OKS:Type 0.9057113 0.1400757           0.9786911 0.1359664          
## 
##      Effects       pes                 
## [1,] "(Intercept)" "0.188331463179584" 
## [2,] "OKS"         "0.0395459892367814"
## [3,] "Type"        "0.417612803926501" 
## [4,] "OKS:Type"    "0.0834483055934803"
## Bayes factor analysis
## --------------
## [1] Type + Subject            : 0.8634068  ±1.68%
## [2] OKS + Subject             : 0.3990639  ±0.55%
## [3] OKS + Type + Subject      : 0.3713146  ±2.2%
## [4] OKS:Type + Subject        : 0.1406391  ±1.1%
## [5] Type + OKS:Type + Subject : 0.1237111  ±4.45%
## [6] OKS + OKS:Type + Subject  : 0.05562135 ±0.95%
## 
## Against denominator:
##   DV ~ Subject 
## ---
## Bayes factor type: BFlinearModel, JZS
```

```
my_analysis(ShiftN1AY)
```

```
## $ANOVA
##        Effect DFn DFd       SSn      SSd          F           p p<.05
## 1 (Intercept)   1  23 625.84240 1113.803 12.9236316 0.001528861     *
## 2         OKS   2  46 299.29951 1816.435  3.7897794 0.029953673     *
## 3        Type   1  23  16.07905 1264.702  0.2924153 0.593875783      
## 4    OKS:Type   2  46 170.41124 2290.665  1.7110570 0.191972855      
##           ges
## 1 0.088004933
## 2 0.044112564
## 3 0.002473059
## 4 0.025602589
## 
## $`Mauchly's Test for Sphericity`
##     Effect         W          p p<.05
## 2      OKS 0.8357216 0.13889230      
## 4 OKS:Type 0.6676585 0.01175163     *
## 
## $`Sphericity Corrections`
##     Effect       GGe      p[GG] p[GG]<.05       HFe      p[HF] p[HF]<.05
## 2      OKS 0.8589011 0.03713238         * 0.9215978 0.03374918         *
## 4 OKS:Type 0.7505583 0.20012075           0.7913620 0.19896650          
## 
##      Effects       pes                 
## [1,] "(Intercept)" "0.359752926173285" 
## [2,] "OKS"         "0.141463627069528" 
## [3,] "Type"        "0.0125541009733948"
## [4,] "OKS:Type"    "0.069242565134114" 
## Bayes factor analysis
## --------------
## [1] OKS + Subject                   : 1.24054   ±1.62%
## [2] OKS + OKS:Type + Subject        : 0.673458  ±1.72%
## [3] OKS:Type + Subject              : 0.560012  ±8.29%
## [4] OKS + Type + Subject            : 0.2606012 ±1.41%
## [5] Type + Subject                  : 0.208219  ±1.56%
## [6] OKS + Type + OKS:Type + Subject : 0.1384626 ±1.56%
## 
## Against denominator:
##   DV ~ Subject 
## ---
## Bayes factor type: BFlinearModel, JZS
```

```
my_analysis(ShiftNRY)
```

```
## $ANOVA
##        Effect DFn DFd       SSn      SSd          F           p p<.05
## 1 (Intercept)   1  23 898.29573 9536.091  2.1665903 0.154590779      
## 2         OKS   2  46  35.22685 3264.180  0.2482147 0.781230583      
## 3        Type   1  23 645.97069 1187.076 12.5158971 0.001758276     *
## 4    OKS:Type   2  46  37.03801 2608.419  0.3265864 0.723040926      
##           ges
## 1 0.051348608
## 2 0.002118145
## 3 0.037465523
## 4 0.002226805
## 
## $`Mauchly's Test for Sphericity`
##     Effect         W         p p<.05
## 2      OKS 0.9486456 0.5599430      
## 4 OKS:Type 0.9052968 0.3347349      
## 
## $`Sphericity Corrections`
##     Effect       GGe     p[GG] p[GG]<.05      HFe     p[HF] p[HF]<.05
## 2      OKS 0.9511540 0.7703823           1.034601 0.7812306          
## 4 OKS:Type 0.9134896 0.7037359           0.988227 0.7205252          
## 
##      Effects       pes                 
## [1,] "(Intercept)" "0.0860899421133626"
## [2,] "OKS"         "0.0106767223038936"
## [3,] "Type"        "0.352402674228931" 
## [4,] "OKS:Type"    "0.0140006083948165"
## Bayes factor analysis
## --------------
## [1] Type + Subject                  : 21.21326   ±1.24%
## [2] Type + OKS:Type + Subject       : 3.076649   ±1.1%
## [3] OKS + Type + Subject            : 1.831289   ±1.68%
## [4] OKS + Type + OKS:Type + Subject : 0.3100446  ±11.95%
## [5] OKS:Type + Subject              : 0.1436118  ±0.64%
## [6] OKS + Subject                   : 0.08447566 ±0.74%
## 
## Against denominator:
##   DV ~ Subject 
## ---
## Bayes factor type: BFlinearModel, JZS
```

Interestingly, this is true also for the vertical plane, thus addition also consistently bring updward shift whereas subtraction brings a downward one.

## Plot results

Another function:

```
my_plot= function(X, lab, legend= F, h= T, lims){
  
  X= melt(X)
  colnames(X)= c("Subject", "OKS", "Type", "DV")
  
  DF= summarySEwithin(data = X, measurevar = "DV", 
                      withinvars = c("Type", "OKS"),
                      idvar = "Subject")
  DF$Type= relevel(DF$Type, "Subtraction")
  
  DF$OKS= factor(DF$OKS, levels= c("Left", "Static", "Right"))
  
  pd=position_dodge(1)
  
  p= ggplot(DF, aes(y= DV, x= OKS, fill= Type)) + 
    geom_bar(position= pd, stat= "identity") + ylab(lab) + commonTheme + 
    geom_errorbar(aes(ymin= DV-se, ymax= DV+se), position= pd, width= .4, size= 1.5, colour= "black") +
    ylim(lims)
  
  #if not
  if(!legend)(p= p + guides(fill= F))
  #if yes
  if(legend) (p= p + theme(legend.position= c(0.5, 0.5),
                      legend.justification= c(0.5, 0.5)) +
              guides(fill= guide_legend(reverse=T, title= "Operation")))
  
    if (h) (p= p  + coord_flip()) 
  
  return(p)
}
```

Horizontal displacement:

```
g_legend<-function(a.gplot){
  tmp <- ggplot_gtable(ggplot_build(a.gplot))
  leg <- which(sapply(tmp$grobs, function(x) x$name) == "guide-box")
  legend <- tmp$grobs[[leg]]
  return(legend)}

grid.arrange(
  a= my_plot(MeanN1X, "Center of Gravity (px) \n First Number", lims= c(-75, 70)),
  b= my_plot(MeanRX, "Center of Gravity (px) \n Response Phase", lims= c(-75, 70)),
  c= my_plot(ShiftN1AX, "Shift (px) \n Alert to First Number", lims= c(-20, 35)),
  d= my_plot(ShiftNRX, "Shift (px) \n Second Number to Response", lims= c(-20, 35)),
  e= g_legend(my_plot(MeanN1X, "I only need the legend here",legend=T, lims= c(-75, 70))),
  layout_matrix = rbind(c(1, 1, 2, 2, 5), c(3, 3, 4, 4, 5)))
```

Vertical Displacement:

```
grid.arrange(
  a= my_plot(MeanN1Y, "Center of Gravity (px) \n First Number", h= F, lims= c(-35, 0)),
  b= my_plot(MeanRY, "Center of Gravity (px) \n Response Phase", h= F, lims= c(-35, 0)),
  c= my_plot(ShiftN1AY, "Shift (px) \n Alert to First Number", h= F, lims= c(-10, 10)),
  d= my_plot(ShiftNRY, "Shift (px) \n Second Number to Response", h= F, lims= c(-10, 10)),
  e= g_legend(my_plot(MeanN1Y, "I only need the legend here",legend=T, h= F, lims= c(-35, 0))),
  layout_matrix = rbind(c(1, 1, 2, 2, 5), c(3, 3, 4, 4, 5)))
```

## Appendix

This is the function to summarise data (`summarySEwithin`):

```
## Summarizes data.
## Gives count, mean, standard deviation, standard error of the mean, and confidence interval (default 95%).
##   data: a data frame.
##   measurevar: the name of a column that contains the variable to be summariezed
##   groupvars: a vector containing names of columns that contain grouping variables
##   na.rm: a boolean that indicates whether to ignore NA's
##   conf.interval: the percent range of the confidence interval (default is 95%)
summarySE <- function(data=NULL, measurevar, groupvars=NULL, na.rm=FALSE,
                      conf.interval=.95, .drop=TRUE) {
  library(plyr)
  
  # New version of length which can handle NA's: if na.rm==T, don't count them
  length2 <- function (x, na.rm=FALSE) {
    if (na.rm) sum(!is.na(x))
    else       length(x)
  }
  
  # This does the summary. For each group's data frame, return a vector with
  # N, mean, and sd
  datac <- ddply(data, groupvars, .drop=.drop,
                 .fun = function(xx, col) {
                   c(N    = length2(xx[[col]], na.rm=na.rm),
                     mean = mean   (xx[[col]], na.rm=na.rm),
                     sd   = sd     (xx[[col]], na.rm=na.rm)
                   )
                 },
                 measurevar
  )
  
  # Rename the "mean" column    
  datac <- plyr::rename(datac, c(mean = measurevar))
  
  datac$se <- datac$sd / sqrt(datac$N)  # Calculate standard error of the mean
  
  # Confidence interval multiplier for standard error
  # Calculate t-statistic for confidence interval: 
  # e.g., if conf.interval is .95, use .975 (above/below), and use df=N-1
  ciMult <- qt(conf.interval/2 + .5, datac$N-1)
  datac$ci <- datac$se * ciMult
  
  return(datac)
}


## Norms the data within specified groups in a data frame; it normalizes each
## subject (identified by idvar) so that they have the same mean, within each group
## specified by betweenvars.
##   data: a data frame.
##   idvar: the name of a column that identifies each subject (or matched subjects)
##   measurevar: the name of a column that contains the variable to be summariezed
##   betweenvars: a vector containing names of columns that are between-subjects variables
##   na.rm: a boolean that indicates whether to ignore NA's
normDataWithin <- function(data=NULL, idvar, measurevar, betweenvars=NULL,
                           na.rm=FALSE, .drop=TRUE) {
  library(plyr)
  
  # Measure var on left, idvar + between vars on right of formula.
  data.subjMean <- ddply(data, c(idvar, betweenvars), .drop=.drop,
                         .fun = function(xx, col, na.rm) {
                           c(subjMean = mean(xx[,col], na.rm=na.rm))
                         },
                         measurevar,
                         na.rm
  )
  
  # Put the subject means with original data
  data <- merge(data, data.subjMean)
  
  # Get the normalized data in a new column
  measureNormedVar <- paste(measurevar, "_norm", sep="")
  data[,measureNormedVar] <- data[,measurevar] - data[,"subjMean"] +
    mean(data[,measurevar], na.rm=na.rm)
  
  # Remove this subject mean column
  data$subjMean <- NULL
  
  return(data)
}


## Retrieved here: http://www.cookbook-r.com/Graphs/Plotting_means_and_error_bars_(ggplot2)/
## Summarizes data, handling within-subjects variables by removing inter-subject variability.
## It will still work if there are no within-S variables.
## Gives count, un-normed mean, normed mean (with same between-group mean),
##   standard deviation, standard error of the mean, and confidence interval.
## If there are within-subject variables, calculate adjusted values using method from Morey (2008).
##   data: a data frame.
##   measurevar: the name of a column that contains the variable to be summariezed
##   betweenvars: a vector containing names of columns that are between-subjects variables
##   withinvars: a vector containing names of columns that are within-subjects variables
##   idvar: the name of a column that identifies each subject (or matched subjects)
##   na.rm: a boolean that indicates whether to ignore NA's
##   conf.interval: the percent range of the confidence interval (default is 95%)
summarySEwithin <- function(data=NULL, measurevar, betweenvars=NULL, withinvars=NULL,
                            idvar=NULL, na.rm=FALSE, conf.interval=.95, .drop=TRUE) {
  
  # Ensure that the betweenvars and withinvars are factors
  factorvars <- vapply(data[, c(betweenvars, withinvars), drop=FALSE],
                       FUN=is.factor, FUN.VALUE=logical(1))
  
  if (!all(factorvars)) {
    nonfactorvars <- names(factorvars)[!factorvars]
    message("Automatically converting the following non-factors to factors: ",
            paste(nonfactorvars, collapse = ", "))
    data[nonfactorvars] <- lapply(data[nonfactorvars], factor)
  }
  
  # Get the means from the un-normed data
  datac <- summarySE(data, measurevar, groupvars=c(betweenvars, withinvars),
                     na.rm=na.rm, conf.interval=conf.interval, .drop=.drop)
  
  # Drop all the unused columns (these will be calculated with normed data)
  datac$sd <- NULL
  datac$se <- NULL
  datac$ci <- NULL
  
  # Norm each subject's data
  ndata <- normDataWithin(data, idvar, measurevar, betweenvars, na.rm, .drop=.drop)
  
  # This is the name of the new column
  measurevar_n <- paste(measurevar, "_norm", sep="")
  
  # Collapse the normed data - now we can treat between and within vars the same
  ndatac <- summarySE(ndata, measurevar_n, groupvars=c(betweenvars, withinvars),
                      na.rm=na.rm, conf.interval=conf.interval, .drop=.drop)
  
  # Apply correction from Morey (2008) to the standard error and confidence interval
  #  Get the product of the number of conditions of within-S variables
  nWithinGroups    <- prod(vapply(ndatac[,withinvars, drop=FALSE], FUN=nlevels,
                                  FUN.VALUE=numeric(1)))
  correctionFactor <- sqrt( nWithinGroups / (nWithinGroups-1) )
  
  # Apply the correction factor
  ndatac$sd <- ndatac$sd * correctionFactor
  ndatac$se <- ndatac$se * correctionFactor
  ndatac$ci <- ndatac$ci * correctionFactor
  
  # Combine the un-normed means with the normed results
  merge(datac, ndatac)
}
```
