# Supplementary material for: Spatial grounding of symbolic arithmetic: an investigation with optokinetic stimulation
Source: Psychol Res. 2018 Jul 18;83(1):64–83. doi: 10.1007/s00426-018-1053-0 (PMC6373542; doi:10.1007/s00426-018-1053-0)
Supplement: Supplementary file 7 — Supplementary material 7 (HTML 2362 KB) [file 426_2018_1053_MOESM7_ESM.html]

Spatial grounding of mental arithmetic - Exp 2, v0


# Spatial grounding of mental arithmetic - Exp 2, v0

#### *Elvio Blini INSERM U1028, ImpAct team, CRNL, and University of Lyon elvio.blini@gmail.com*

#### *10 May 2017*

# Experiment 2

# Behavioural data

This document includes data and analyses for the second experiment described in the companion paper.

We administered vertical OKS while participants were cuncurrently asked to perform mental arithmetic.

We focus, as in Exp 1, on the distribution of responses, isolating estimation and decade errors, when assessing behavioural performance.

For eye-tracking data, we focus on the center of gravity of gaze during different phases of calculation, and the displacement occurring between two consecutive phases.

We observed, in Exp 1, that gaze was affected by the Operation type (subtraction leading to leftward and downward gaze shifts, addition to rightward and upward ones). This was interesting given that the vertical dimension was not manipulated by OKS (which was horizontal). It follows that the vertical dimension might be more relevant, and here’s why we administered a vertical stimulation.

I am very very sorry for variables having Italian names… Early career scripts in E-Prime… I’ll provide relevant translations in the text.

For any request or inquiry don’t hesitate contacting: elvio.blini@gmail.com

## Preliminary setup

It’s sometimes good to clean the current environment to avoid conflicts. You can do it with `rm(list=ls())` (but be sure everything is properly saved for future use).

In order to run this script we need a few packages available on CRAN. You might need to install them first, e.g. by typing `install.packages("BayesFactor")` in the console.

```
#list packages
packages= c("plyr", "BayesFactor", "reshape", "gridExtra", "ez", "tidyverse")

#load them
lapply(packages, require, character.only= T)
```

Thanks to the function retrieved here, not displayed, the following hyperlink downloads the Rdata file:

That can be loaded then with:

```
load("Exp 2 data.RData")
```

Now all relevant variables are stored in the `data` data.frame, that you can navigate and explore with the usual commands, e.g. `str(data)`.

## Useful functions

This is a convenient (minimal) list of ggplot attributes shared by different plots.

```
#ggplot defaults
commonTheme = list(theme_bw(),
                   theme(text= element_text(size=22, face="bold")),
                   scale_fill_grey(start = 0, end = .8),
                   guides(fill= F))
```

The package ez, and the function ezANOVA, does not return partial eta square by default. So, here it is:

```
extract.pes= function(EZ){
  return(
    cbind(Effects= EZ$ANOVA$Effect, pes= EZ$ANOVA$SSn/(EZ$ANOVA$SSn + EZ$ANOVA$SSd)))
}
```

Then (NOT SHOWN in the html file but shown in the source) we have a convenient summarising function that produces means and within subjects SEM as in Morey (2008). I retrieved it here: link

## Preprocessing

The factors OKS (SOK) and Deviant eye movement (Mosso) are to be converted into factors.

Response (Risposta.RESP) and Correct response (Risposta.CRESP) are numeric instead (NAs from coercion are expected).

```
#declare
data$SOK<-factor(data$SOK)
data$Mosso<-factor(data$Mosso)

data$Risposta.RESP<-as.numeric(as.character(data$Risposta.RESP))
data$Risposta.CRESP<-as.numeric(as.character(data$Risposta.CRESP))
```

We create an Accuracy (Accuratezza) variable and a convenience variable storing only “1” (Uno).

```
data$Accuratezza<-ifelse(data$Risposta.RESP == data$Risposta.CRESP, 1, 0)
data$Uno<-seq(1,1,length=nrow(data))
```

We drop practice trials.

```
 data<-data[data$Procedure=="TrialProc",]
```

I rename levels of Sign (Segno) as to be intelligible!

```
if(sum(levels(data$Segno)==c("meno", "piu"))==2)(
levels(data$Segno)= c("Subtraction", "Addition")) else(stop("Unordered levels"))
```

```
## [1] "Subtraction" "Addition"
```

```
#now reorder levels
data$Segno= relevel(data$Segno, "Addition")
```

Same for OKS (SOK).

```
data$SOK<-factor(data$SOK)
data$SOK= factor(data$SOK, levels=c("basso", "staticaV", "alto"), 
                           labels=c("Down", "Static", "Up"))
```

Trials in which participants refused categorically to respond (dropped, return percentage dropped).

```
#subj refuses to respond
numtrial1<-sum(data$Uno)
data<-data[!is.na(data$Risposta.RESP),]
numtrial2<-sum(data$Uno)

rispostenonfornite<-((numtrial2-numtrial1)/numtrial1)*100
rispostenonfornite
```

```
## [1] -0.06936416
```

So we were scary enough to force them!

### Accuracy

We process and summarise accuracy:

```
ACC= data %>% 
  filter(Mosso== 0) %>% #invalid OKS
  ddply(c("Segno", "SOK", "Subject"), summarise, N= length(SecondoO.RT), ACC= mean(Accuratezza), .drop=F)
```

Overall, mean accuracy was:

```
ACC %>% with(tapply(ACC, list(Segno, SOK), mean))
```

```
##                  Down    Static        Up
## Addition    0.6670311 0.6632547 0.6393671
## Subtraction 0.5162867 0.4719891 0.4967342
```

```
ACC %>% with(tapply(ACC, list(Segno, SOK), sd))
```

```
##                  Down    Static        Up
## Addition    0.2588031 0.2638211 0.2410421
## Subtraction 0.2955892 0.2827901 0.3001045
```

### RTs

We process and summarise reaction times:

```
RTs= data %>% 
  mutate(SecondoO.RT= as.numeric(as.character(SecondoO.RT))) %>% #is actually a number, not a factor
  filter(Mosso== 0) %>% #invalid OKS
  filter(SecondoO.RT> 500) %>% #exclude anticipations
  filter(Accuratezza== 1) %>% #only accurate responses
  ddply(c("Segno", "SOK", "Subject"), summarise, N= length(SecondoO.RT), RT= mean(SecondoO.RT), .drop=F) %>%
  filter(!Subject %in% c(10, 12, 23)) #not enough trials per cell
```

Overall, RTs are:

```
RTs %>% with(tapply(RT, list(Segno, SOK), mean))
```

```
##                 Down   Static       Up
## Addition    4043.143 3869.706 3885.579
## Subtraction 4163.312 4036.940 4067.968
```

```
RTs %>% with(tapply(RT, list(Segno, SOK), sd))
```

```
##                 Down   Static       Up
## Addition    501.3486 474.4009 624.7407
## Subtraction 575.4171 790.3079 511.4947
```

### Errors distribution

I create a variable for the shift from the correct response.

```
#pure shift measure
data$Shift<-data$Risposta.RESP-data$Risposta.CRESP
```

And assess its distribution.

```
ggplot(data, aes(x= Shift)) + geom_density() + facet_wrap("Segno") + 
  commonTheme + scale_x_continuous(breaks= seq(-30, 30, 10), limits = c(-30, 30))
```

```
data$Error_type= with(data, ifelse(Shift== 0, "Correct Response", 
                                   ifelse(abs(Shift)== 10 | abs(Shift)== 20 | abs(Shift)== 30 | abs(Shift)== 40,  "Decade Error",
                                          "Estimation Error")))

data %>% ddply("Subject", count, Error_type) %>%
  ddply("Subject", mutate, percentage= n/sum(n)) %>%
  ddply("Error_type", summarise, Error_Type= mean(percentage)) %>%
  ggplot(aes(x= "", y= Error_Type, fill= Error_type)) + 
  xlab(NULL) + ylab(NULL) + labs(fill="Error Type") +
  geom_bar(width = 1, stat = "identity") +
  coord_polar("y", start= 0) + commonTheme
```

There are multiple peaks in correspondence of decades. Exclude deviant eye movements (Mosso==1), return percentage of trials dropped.

```
#exclude eye movements
data<-data[! is.na (data$Shift),]
A<-sum(data$Uno)
data<-data[data$Mosso=="0",]
B<-sum(data$Uno)
Escl.mossi.shift<-((A-B)/A)*100
Escl.mossi.shift
```

```
## [1] 9.231837
```

## Analyses

### Accuracy

```
(EZ.acc=ezANOVA(data= ACC, dv = .(ACC), within = .("SOK","Segno"), 
        type = 3, detailed = T, wid= .(Subject)))
```

```
## $ANOVA
##        Effect DFn DFd         SSn       SSd           F            p p<.05
## 1 (Intercept)   1  23 47.73878255 8.8262494 124.4007453 9.379072e-11     *
## 2         SOK   2  46  0.01816497 0.6410400   0.6517446 5.258805e-01      
## 3       Segno   1  23  0.93951505 0.5818114  37.1406373 3.237786e-06     *
## 4   SOK:Segno   2  46  0.01629165 0.3489209   1.0739050 3.500820e-01      
##           ges
## 1 0.821145627
## 2 0.001743918
## 3 0.082867653
## 4 0.001564351
## 
## $`Mauchly's Test for Sphericity`
##      Effect         W         p p<.05
## 2       SOK 0.8240766 0.1190265      
## 4 SOK:Segno 0.9137169 0.3706193      
## 
## $`Sphericity Corrections`
##      Effect       GGe     p[GG] p[GG]<.05       HFe     p[HF] p[HF]<.05
## 2       SOK 0.8503955 0.5027398           0.9112776 0.5125963          
## 4 SOK:Segno 0.9205703 0.3461902           0.9969199 0.3499409
```

We now plot results:

```
ACC %>% 
  summarySEwithin(measurevar = "ACC", withinvars = c("Segno", "SOK"), idvar = "Subject") %>%
  ggplot(aes(y= ACC, x= SOK, fill= SOK)) +  
  geom_errorbar(aes(ymin= ACC-se, ymax= ACC+se), width= .4, size= 1.5, colour= "black") +
  geom_point(shape= 21, size= 6, stroke= 2) + facet_wrap("Segno", ncol= 2) + xlab("OKS") + 
  scale_fill_discrete(guide=F) + ylab("Accuracy") +
  commonTheme
```

We only found a main effect of Operation type, Additions being more accurate than subtractions, but no OKS effects.

### RTs

```
(EZ.rts=ezANOVA(data= RTs, dv = .(RT), within = .("SOK","Segno"), 
        type = 3, detailed = T, wid= .(Subject)))
```

```
## $ANOVA
##        Effect DFn DFd          SSn      SSd            F            p
## 1 (Intercept)   1  20 2.027212e+09 26642208 1521.8051232 2.391002e-20
## 2         SOK   2  40 5.461669e+05  4608633    2.3701904 1.064649e-01
## 3       Segno   1  20 7.724713e+05  2655037    5.8189129 2.558043e-02
## 4   SOK:Segno   2  40 2.210629e+04  7774640    0.0568677 9.447953e-01
##   p<.05          ges
## 1     * 0.9798537100
## 2       0.0129341652
## 3     * 0.0181959229
## 4       0.0005300936
## 
## $`Mauchly's Test for Sphericity`
##      Effect         W          p p<.05
## 2       SOK 0.9357871 0.53233113      
## 4 SOK:Segno 0.7078004 0.03751048     *
## 
## $`Sphericity Corrections`
##      Effect       GGe     p[GG] p[GG]<.05       HFe     p[HF] p[HF]<.05
## 2       SOK 0.9396616 0.1102460           1.0337855 0.1064649          
## 4 SOK:Segno 0.7738742 0.9060026           0.8265311 0.9171236
```

We now plot results:

```
RTs %>% 
  summarySEwithin(measurevar = "RT", withinvars = c("Segno", "SOK"), idvar = "Subject") %>%
  ggplot(aes(y= RT, x= SOK, fill= SOK)) +  
  geom_errorbar(aes(ymin= RT-se, ymax= RT+se), width= .4, size= 1.5, colour= "black") +
  geom_point(shape= 21, size= 6, stroke= 2) + facet_wrap("Segno", ncol= 2) + xlab("OKS") + 
  scale_fill_discrete(guide=F) +
  commonTheme
```

We only found a main effect of Operation type, Additions being faster than subtractions, but no OKS effects.

### Displacement - estimation errors

We can now prepare data frames for ANOVA purposes.

```
X<- data #safe version of "data" 
X$Subject=as.factor(X$Subject)
```

We identify positive & negative decades errors, then exclude them to isolate estimations.

```
X$PDE<-ifelse(X$Shift==10 |X$Shift==20|X$Shift==30|X$Shift==40, 1, 0)
X$NDE<-ifelse(X$Shift==-10|X$Shift==-20|X$Shift==-30|X$Shift==-40, 1, 0)

Y=X[X$PDE==0 & X$NDE==0,]
1-nrow(Y)/nrow(X) #decade errors, proportion
```

```
## [1] 0.09686464
```

We summarise and run ANOVA.

```
#summarise
DFshift=ddply(Y, c("Subject","SOK","Segno"), summarise,
              Shift = mean(Shift))
DFshift$Subject=as.factor(DFshift$Subject)

(EZ1=ezANOVA(data=DFshift, dv = .(Shift), within = .("SOK","Segno"), 
        type = 3, detailed = T, wid= .(Subject)))
```

```
## $ANOVA
##        Effect DFn DFd       SSn       SSd         F           p p<.05
## 1 (Intercept)   1  23 11.445508  89.14659 2.9529640 0.099154617      
## 2         SOK   2  46  2.676695  72.49581 0.8492075 0.434349494      
## 3       Segno   1  23 53.971481 154.32058 8.0439306 0.009357142     *
## 4   SOK:Segno   2  46  3.819631  54.18130 1.6214358 0.208704871      
##           ges
## 1 0.029994271
## 2 0.007179572
## 3 0.127256481
## 4 0.010213902
## 
## $`Mauchly's Test for Sphericity`
##      Effect         W         p p<.05
## 2       SOK 0.9893535 0.8889277      
## 4 SOK:Segno 0.9358528 0.4822622      
## 
## $`Sphericity Corrections`
##      Effect       GGe     p[GG] p[GG]<.05      HFe     p[HF] p[HF]<.05
## 2       SOK 0.9894657 0.4333526           1.082113 0.4343495          
## 4 SOK:Segno 0.9397197 0.2104137           1.020488 0.2087049
```

Only a main effect of Sign (thus, operation type). Let’s have partial eta squared:

```
extract.pes(EZ1)
```

```
##      Effects       pes                 
## [1,] "(Intercept)" "0.113781376650084" 
## [2,] "SOK"         "0.035607368474202" 
## [3,] "Segno"       "0.259114435939073" 
## [4,] "SOK:Segno"   "0.0658546415964156"
```

A Bayesian counterpart:

```
BF1= anovaBF(formula = Shift ~ SOK*Segno + Subject, data = DFshift,whichModels = "all", whichRandom = "Subject")
head(BF1)
```

When compared against the model 

...

Enter one or more search terms in the box to filter the models in the table. If more than one term is included, matching will be performed with `or`. Special search terms are allowed:

| Code | Function | Example | What example does |
| --- | --- | --- | --- |
| + | Require this term in search results | +shape | Requires all models to include âshapeâ |
| - | Require this term NOT to appear in search results | -shape | Requires all models to exclude âshapeâ |
| # (with number) | Return results with certain number of terms | #2 | Requires all models to have two terms |
| @ (with :, ::, â¦) | Return results containing interactions of certain size | @:: | Requires all models to have a three-way interaction |
| < or > | Limits sizes of Bayes factors | >2 | Returns models whose Bayes factor is greater than 2 |

Click on a row in the Bayes factor table to make that model the denominator. Sort by clicking on the arrows in the column headers.

|  |  |  |
| --- | --- | --- |
| ...the model below... | ...is preferred by... |  |

Main effect of Operation type confirmed. We can now plot the results (first summarised with `summarySE`).

```
#summarise then plot shift
DF= summarySEwithin(data = DFshift, measurevar = "Shift", withinvars = c("Segno", "SOK"),
                    idvar = "Subject")

ggplot(DF, aes(y= Shift, x= SOK, fill= SOK)) + geom_bar(position= "dodge", stat= "identity") + 
  facet_wrap("Segno", nrow= 2) + xlab("OKS") + scale_fill_discrete(guide=F) + ylim(c(-2, 3)) +
  geom_errorbar(aes(ymin= Shift-se, ymax= Shift+se), width= .4, size= 1.5, colour= "black") +
  coord_flip() + commonTheme
```

## Positive decade errors

I’m summarising asking for the proportion (mean, since this values is binomial) of positive errors. Then we use asin transform on data.

```
positive=ddply(X, c("Subject","SOK","Segno"), summarise,
               PDE = mean(PDE))
positive$asinPDE=asin(sqrt(positive$PDE))
```

Now anova and partial eta square.

```
(EZ2= ezANOVA(data=positive, dv = .(asinPDE), within = .("SOK","Segno"), 
        type = 3, detailed = T, wid= .(Subject)))
```

```
## $ANOVA
##        Effect DFn DFd       SSn       SSd          F            p p<.05
## 1 (Intercept)   1  23 2.9479048 1.0379237 65.3244689 3.596391e-08     *
## 2         SOK   2  46 0.0211450 0.6485729  0.7498541 4.781217e-01      
## 3       Segno   1  23 0.2343825 0.3957426 13.6219776 1.208349e-03     *
## 4   SOK:Segno   2  46 0.1402571 0.5119373  6.3013837 3.813408e-03     *
##           ges
## 1 0.531912958
## 2 0.008085048
## 3 0.082862862
## 4 0.051292926
## 
## $`Mauchly's Test for Sphericity`
##      Effect         W         p p<.05
## 2       SOK 0.9390116 0.5004731      
## 4 SOK:Segno 0.9757650 0.7634798      
## 
## $`Sphericity Corrections`
##      Effect       GGe      p[GG] p[GG]<.05      HFe       p[HF] p[HF]<.05
## 2       SOK 0.9425174 0.47111410           1.023938 0.478121737          
## 4 SOK:Segno 0.9763384 0.00411716         * 1.065795 0.003813408         *
```

```
extract.pes(EZ2)
```

```
##      Effects       pes                
## [1,] "(Intercept)" "0.739596509558849"
## [2,] "SOK"         "0.031572996417804"
## [3,] "Segno"       "0.371961823714416"
## [4,] "SOK:Segno"   "0.215054134233053"
```

This time we found, beside the effect of Operation type (Segno), an interaction with OKS!

Note that this is confirmed when using non-asin transformed values:

```
ezANOVA(data=positive, dv = .(PDE), within = .("SOK","Segno"), 
        type = 3, detailed = T, wid= .(Subject))
```

```
## $ANOVA
##        Effect DFn DFd         SSn        SSd         F            p p<.05
## 1 (Intercept)   1  23 0.227314495 0.11960074 43.714056 9.567298e-07     *
## 2         SOK   2  46 0.003894614 0.06894719  1.299199 2.825694e-01      
## 3       Segno   1  23 0.041883139 0.06770869 14.227304 9.894892e-04     *
## 4   SOK:Segno   2  46 0.010666489 0.05946780  4.125413 2.249489e-02     *
##          ges
## 1 0.41859706
## 2 0.01218518
## 3 0.11712040
## 4 0.03268011
## 
## $`Mauchly's Test for Sphericity`
##      Effect         W         p p<.05
## 2       SOK 0.8821322 0.2516927      
## 4 SOK:Segno 0.9802872 0.8033168      
## 
## $`Sphericity Corrections`
##      Effect       GGe      p[GG] p[GG]<.05       HFe      p[HF] p[HF]<.05
## 2       SOK 0.8945602 0.28140810           0.9650446 0.28226305          
## 4 SOK:Segno 0.9806683 0.02326861         * 1.0711726 0.02249489         *
```

Confirmed also by a Bayesian counterpart:

```
BF2= anovaBF(formula = asinPDE ~ SOK*Segno + Subject, data = positive,whichModels = "all", whichRandom = "Subject" )
head(BF2)
```

When compared against the model 

...

Enter one or more search terms in the box to filter the models in the table. If more than one term is included, matching will be performed with `or`. Special search terms are allowed:

| Code | Function | Example | What example does |
| --- | --- | --- | --- |
| + | Require this term in search results | +shape | Requires all models to include âshapeâ |
| - | Require this term NOT to appear in search results | -shape | Requires all models to exclude âshapeâ |
| # (with number) | Return results with certain number of terms | #2 | Requires all models to have two terms |
| @ (with :, ::, â¦) | Return results containing interactions of certain size | @:: | Requires all models to have a three-way interaction |
| < or > | Limits sizes of Bayes factors | >2 | Returns models whose Bayes factor is greater than 2 |

Click on a row in the Bayes factor table to make that model the denominator. Sort by clicking on the arrows in the column headers.

|  |  |  |
| --- | --- | --- |
| ...the model below... | ...is preferred by... |  |

```
head(BF2)[1]/head(BF2)[2] #model with the interaction 6.5 times more likely than the one without it
```

When compared against the model 

...

Enter one or more search terms in the box to filter the models in the table. If more than one term is included, matching will be performed with `or`. Special search terms are allowed:

| Code | Function | Example | What example does |
| --- | --- | --- | --- |
| + | Require this term in search results | +shape | Requires all models to include âshapeâ |
| - | Require this term NOT to appear in search results | -shape | Requires all models to exclude âshapeâ |
| # (with number) | Return results with certain number of terms | #2 | Requires all models to have two terms |
| @ (with :, ::, â¦) | Return results containing interactions of certain size | @:: | Requires all models to have a three-way interaction |
| < or > | Limits sizes of Bayes factors | >2 | Returns models whose Bayes factor is greater than 2 |

Click on a row in the Bayes factor table to make that model the denominator. Sort by clicking on the arrows in the column headers.

|  |  |  |
| --- | --- | --- |
| ...the model below... | ...is preferred by... |  |

We now run post-hoc analyses. We focus on the difference between addition and subtraction (in terms of proportion of positive decade errors) for each OKS condition.

For downward OKS:

```
t.test(positive$asinPDE[positive$Segno=="Subtraction" & positive$SOK=="Down"], 
        positive$asinPDE[positive$Segno=="Addition" & positive$SOK=="Down"], paired=T)
```

```
## 
##  Paired t-test
## 
## data:  positive$asinPDE[positive$Segno == "Subtraction" & positive$SOK ==  and positive$asinPDE[positive$Segno == "Addition" & positive$SOK ==     "Down"] and     "Down"]
## t = -0.15874, df = 23, p-value = 0.8753
## alternative hypothesis: true difference in means is not equal to 0
## 95 percent confidence interval:
##  -0.05994676  0.05140245
## sample estimates:
## mean of the differences 
##            -0.004272154
```

```
ttestBF(positive$asinPDE[positive$Segno=="Subtraction" & positive$SOK=="Down"], 
        positive$asinPDE[positive$Segno=="Addition" & positive$SOK=="Down"], paired=T)
```

When compared against the model 

...

Enter one or more search terms in the box to filter the models in the table. If more than one term is included, matching will be performed with `or`. Special search terms are allowed:

| Code | Function | Example | What example does |
| --- | --- | --- | --- |
| + | Require this term in search results | +shape | Requires all models to include âshapeâ |
| - | Require this term NOT to appear in search results | -shape | Requires all models to exclude âshapeâ |
| # (with number) | Return results with certain number of terms | #2 | Requires all models to have two terms |
| @ (with :, ::, â¦) | Return results containing interactions of certain size | @:: | Requires all models to have a three-way interaction |
| < or > | Limits sizes of Bayes factors | >2 | Returns models whose Bayes factor is greater than 2 |

Click on a row in the Bayes factor table to make that model the denominator. Sort by clicking on the arrows in the column headers.

|  |  |  |
| --- | --- | --- |
| ...the model below... | ...is preferred by... |  |

There is no difference in the proportion of decade errors. Actually, the absence of a difference is more likely (BF<0.3). But this difference exists in the baseline - static condition:

```
t.test(positive$asinPDE[positive$Segno=="Subtraction" & positive$SOK=="Static"], 
        positive$asinPDE[positive$Segno=="Addition" & positive$SOK=="Static"], paired=T)
```

```
## 
##  Paired t-test
## 
## data:  positive$asinPDE[positive$Segno == "Subtraction" & positive$SOK ==  and positive$asinPDE[positive$Segno == "Addition" & positive$SOK ==     "Static"] and     "Static"]
## t = 2.9909, df = 23, p-value = 0.006528
## alternative hypothesis: true difference in means is not equal to 0
## 95 percent confidence interval:
##  0.03158239 0.17326685
## sample estimates:
## mean of the differences 
##               0.1024246
```

```
ttestBF(positive$asinPDE[positive$Segno=="Subtraction" & positive$SOK=="Static"], 
        positive$asinPDE[positive$Segno=="Addition" & positive$SOK=="Static"], paired=T)
```

When compared against the model 

...

Enter one or more search terms in the box to filter the models in the table. If more than one term is included, matching will be performed with `or`. Special search terms are allowed:

| Code | Function | Example | What example does |
| --- | --- | --- | --- |
| + | Require this term in search results | +shape | Requires all models to include âshapeâ |
| - | Require this term NOT to appear in search results | -shape | Requires all models to exclude âshapeâ |
| # (with number) | Return results with certain number of terms | #2 | Requires all models to have two terms |
| @ (with :, ::, â¦) | Return results containing interactions of certain size | @:: | Requires all models to have a three-way interaction |
| < or > | Limits sizes of Bayes factors | >2 | Returns models whose Bayes factor is greater than 2 |

Click on a row in the Bayes factor table to make that model the denominator. Sort by clicking on the arrows in the column headers.

|  |  |  |
| --- | --- | --- |
| ...the model below... | ...is preferred by... |  |

And it is magnified by upward OKS:

```
t.test(positive$asinPDE[positive$Segno=="Subtraction" & positive$SOK=="Up"], 
        positive$asinPDE[positive$Segno=="Addition" & positive$SOK=="Up"], paired=T)
```

```
## 
##  Paired t-test
## 
## data:  positive$asinPDE[positive$Segno == "Subtraction" & positive$SOK ==  and positive$asinPDE[positive$Segno == "Addition" & positive$SOK ==     "Up"] and     "Up"]
## t = 3.8578, df = 23, p-value = 0.0008003
## alternative hypothesis: true difference in means is not equal to 0
## 95 percent confidence interval:
##  0.06674307 0.22108265
## sample estimates:
## mean of the differences 
##               0.1439129
```

```
ttestBF(positive$asinPDE[positive$Segno=="Subtraction" & positive$SOK=="Up"], 
        positive$asinPDE[positive$Segno=="Addition" & positive$SOK=="Up"], paired=T)
```

When compared against the model 

...

Enter one or more search terms in the box to filter the models in the table. If more than one term is included, matching will be performed with `or`. Special search terms are allowed:

| Code | Function | Example | What example does |
| --- | --- | --- | --- |
| + | Require this term in search results | +shape | Requires all models to include âshapeâ |
| - | Require this term NOT to appear in search results | -shape | Requires all models to exclude âshapeâ |
| # (with number) | Return results with certain number of terms | #2 | Requires all models to have two terms |
| @ (with :, ::, â¦) | Return results containing interactions of certain size | @:: | Requires all models to have a three-way interaction |
| < or > | Limits sizes of Bayes factors | >2 | Returns models whose Bayes factor is greater than 2 |

Click on a row in the Bayes factor table to make that model the denominator. Sort by clicking on the arrows in the column headers.

|  |  |  |
| --- | --- | --- |
| ...the model below... | ...is preferred by... |  |

## Negative decade errors

I’m summarising asking for the proportion (mean, since this values is binomial) of negative errors. Then we use asin transform on data.

```
negative=ddply(X, c("Subject","SOK","Segno"), summarise,
               NDE = mean(NDE))
negative$asinNDE=asin(sqrt(negative$NDE))
```

Now anova and partial eta square.

```
(EZ3= ezANOVA(data=negative, dv = .(asinNDE), within = .("SOK","Segno"), 
        type = 3, detailed = T, wid= .(Subject)))
```

```
## $ANOVA
##        Effect DFn DFd        SSn       SSd           F            p p<.05
## 1 (Intercept)   1  23 5.78313962 1.3116961 101.4047468 6.700849e-10     *
## 2         SOK   2  46 0.01571405 0.7965696   0.4537246 6.380700e-01      
## 3       Segno   1  23 0.02088354 0.4845447   0.9912839 3.297889e-01      
## 4   SOK:Segno   2  46 0.03759199 0.5246370   1.6480266 2.035873e-01      
##           ges
## 1 0.649748109
## 2 0.005015398
## 3 0.006654345
## 4 0.011914904
## 
## $`Mauchly's Test for Sphericity`
##      Effect         W         p p<.05
## 2       SOK 0.8947492 0.2942487      
## 4 SOK:Segno 0.9431288 0.5251469      
## 
## $`Sphericity Corrections`
##      Effect      GGe     p[GG] p[GG]<.05       HFe     p[HF] p[HF]<.05
## 2       SOK 0.904772 0.6189459           0.9775405 0.6337260          
## 4 SOK:Segno 0.946189 0.2052434           1.0284691 0.2035873
```

```
extract.pes(EZ3)
```

```
##      Effects       pes                 
## [1,] "(Intercept)" "0.815119594793363" 
## [2,] "SOK"         "0.0193455234132262"
## [3,] "Segno"       "0.0413185018194721"
## [4,] "SOK:Segno"   "0.066862417413413"
```

This time we found nothing to be significant. A Bayesian counterpart:

```
BF3= anovaBF(formula = asinNDE ~ SOK*Segno + Subject, data = negative,whichModels = "all", whichRandom = "Subject" )
head(BF3)
```

When compared against the model 

...

Enter one or more search terms in the box to filter the models in the table. If more than one term is included, matching will be performed with `or`. Special search terms are allowed:

| Code | Function | Example | What example does |
| --- | --- | --- | --- |
| + | Require this term in search results | +shape | Requires all models to include âshapeâ |
| - | Require this term NOT to appear in search results | -shape | Requires all models to exclude âshapeâ |
| # (with number) | Return results with certain number of terms | #2 | Requires all models to have two terms |
| @ (with :, ::, â¦) | Return results containing interactions of certain size | @:: | Requires all models to have a three-way interaction |
| < or > | Limits sizes of Bayes factors | >2 | Returns models whose Bayes factor is greater than 2 |

Click on a row in the Bayes factor table to make that model the denominator. Sort by clicking on the arrows in the column headers.

|  |  |  |
| --- | --- | --- |
| ...the model below... | ...is preferred by... |  |

## Plot decade errors

Switch to percentage.

```
positive$PDE= positive$PDE*100
negative$NDE= negative$NDE*100
```

Distribution/skewness:

```
positive %>% ddply(c("Subject"), summarise, PDE= mean(PDE)) %>% 
  ggplot(aes(x= PDE)) + 
  geom_histogram(bins= 10, fill=I("blue"), col=I("red"), alpha=I(.2), size= 1.2) + 
  commonTheme
```

```
negative %>% ddply(c("Subject"), summarise, NDE= mean(NDE)) %>% 
  ggplot(aes(x= NDE)) + 
  geom_histogram(bins= 10, fill=I("blue"), col=I("red"), alpha=I(.2), size= 1.2) + 
  commonTheme
```

Negative errors (summarise + plot):

```
DFn= summarySEwithin(data = negative, measurevar = "NDE", withinvars = c("Segno", "SOK"),
                     idvar = "Subject")

n= ggplot(DFn, aes(y= NDE, x= SOK, fill= SOK)) + geom_bar(position= "dodge", stat= "identity") + 
  facet_wrap("Segno", ncol= 2) + xlab("OKS") + scale_fill_discrete(guide=F) + ylim(c(0, 12.5)) +
  geom_errorbar(aes(ymin= NDE-se, ymax= NDE+se), width= .4, size= 1.5, colour= "black") +
  ylab("Percent of Negative Decade Errors (%)") + commonTheme
```

Now same for positive:

```
DFp= summarySEwithin(data = positive, measurevar = "PDE", withinvars = c("Segno", "SOK"),
                     idvar = "Subject")

p= ggplot(DFp, aes(y= PDE, x= SOK, fill= SOK)) + geom_bar(position= "dodge", stat= "identity") + 
  facet_wrap("Segno", ncol= 2) + xlab("OKS") + scale_fill_discrete(guide=F) + ylim(c(0, 12.5)) +
  geom_errorbar(aes(ymin= PDE-se, ymax= PDE+se), width= .4, size= 1.5, colour= "black") +
  ylab("Percent of Positive Decade Errors (%)") + commonTheme
```

Arrange the two plots:

```
grid.arrange(p, n, ncol=2)
```

# Eye-tracking data

I suggest to run `rm(list=ls())` again. We will need to load behavioural data again (we need the rawest form possible) and then eye movements data. For behavioural data:

```
load("Exp 2 data.RData")
```

You can download eye tracking data here:

That can be loaded then with:

```
load("Exp 2 data eye tracking.RData")
```

Now all relevant eye tracking variables are stored in the `MOC` data.frame, that you can navigate and explore with the usual commands, e.g. `str(MOC)`. Please note that eye tracking data have been summarised already for each subject and trial because each individual raw file weights up to >60-70 MB…

## Preprocessing

For behavioural data similar as above.

```
#declare
data$SOK<-factor(data$SOK)
data$Mosso<-factor(data$Mosso)

data$Risposta.RESP<-as.numeric(as.character(data$Risposta.RESP))
data$Risposta.CRESP<-as.numeric(as.character(data$Risposta.CRESP))

data$Accuratezza<-ifelse(data$Risposta.RESP == data$Risposta.CRESP, 1, 0)
data$Shift<-data$Risposta.RESP-data$Risposta.CRESP

data$Uno<-seq(1,1,length=nrow(data))

#exclude practice
data<-data[data$Procedure=="TrialProc",]
```

I miss a variable indicating the trial number:

```
PONTE<-{}
for (i in 1:max(as.numeric(as.character(data$Subject)))) {
  TEMP<-data[data$Subject== i,]
  
  TEMP$TrialN<-seq(1:max(nrow(TEMP)))
  PONTE<-rbind(PONTE, TEMP)
}
data<-PONTE
```

We need to retain only trials for which we also have eye tracking data (thus, no eye tracking failures of some sort).

```
PONTE<-{}
for (i in 1:max(as.numeric(as.character(data$Subject)))) {
  TEMPED<-data[data$Subject== i,]
  TEMPMOC<-MOC[MOC$Subject== i,]
  
  TEMPED<-TEMPED[TEMPMOC$NumTrial,]
  PONTE<-rbind(PONTE, TEMPED)
}
data<-PONTE
```

We can now start working on the MOC dataframe. First all variables indicating the center of gravity must be declared numeric.

```
names(MOC)
```

```
##  [1] "NumTrial"     "MeanAX"       "MeanN1X"      "MeanSX"      
##  [5] "MeanN2X"      "MeanRX"       "MeanAY"       "MeanN1Y"     
##  [9] "MeanSY"       "MeanN2Y"      "MeanRY"       "ShiftNRX"    
## [13] "ShiftNRY"     "SumDifRX"     "SumDifRY"     "ReacTim"     
## [17] "Mosso"        "Corretta"     "Segno"        "SOK"         
## [21] "SecondoORESP" "Grand"        "Uno"          "RispostaRESP"
## [25] "Shift"        "Subject"
```

```
for(i in 1:(ncol(MOC)-11)) {
  MOC[,i] <- as.numeric(as.character(MOC[,i]))
}
```

Values refers to gaze on the x and y axis. We want to code it with respect to center of the screen, so that the center of gravity refers to the geometrical coordinates of it. We thus subtract half the horizontal screen resolution from x and y, and we additionally flip y (because 0 was coded as the upper corner of the screen which is quite confusing to me).

```
for(i in 2:6) {
  MOC[,i] <- (MOC[,i]) - 512
}

for(i in 7:11) {
  MOC[,i] <- (((MOC[,i]) - 768) * (-1))-384
}

#shiftY only has to be flipped
MOC$ShiftNRY<-(-1)*MOC$ShiftNRY
```

Renaming levels.

```
if(sum(levels(MOC$Segno)==c("meno", "piu"))==2)(
  levels(MOC$Segno)= c("Subtraction", "Addition")) else(stop("Unordered levels"))
```

```
## [1] "Subtraction" "Addition"
```

```
  MOC$Segno= relevel(MOC$Segno, "Addition")
  
  MOC$SOK= factor(MOC$SOK, levels=c("basso", "staticaV", "alto"), 
                   labels=c("Down", "Static", "Up"))

#rename variables
colnames(MOC)[colnames(MOC)=="SOK"]= "OKS"  
colnames(MOC)[colnames(MOC)=="Segno"]= "Type"
```

## Summarise

A custom function that returns a tapply object for a given parameter as a function of OKS and Operation type, by subject, and print necessary omissions in computing it (i.e. absence of samples).

```
my_Summary= function(DF= MOC, dv){
  conta<-sum(DF$Uno)
  SenzaNA<-DF[! is.na(DF[,dv]),]
  conta2<-sum(SenzaNA$Uno)
  perc.escl<- ((conta2-conta)/conta)*100
  print(perc.escl)
  MeanSOGG<-tapply(na.omit(SenzaNA[, dv]), list(SenzaNA$Subject, SenzaNA$OKS,SenzaNA$Type),mean)
  return(MeanSOGG)
}
```

It can be used for any index. Here we focus on those reported in the paper.

```
#center of gravity, first number (x and y)
MeanN1X= my_Summary(dv= "MeanN1X")
```

```
## [1] -0.6390593
```

```
MeanN1Y= my_Summary(dv= "MeanN1Y")
```

```
## [1] -0.6390593
```

```
#printed values, omissions, are the same because of course samples x and y are coupled

#center of gravity, first number (x and y)
MeanRX= my_Summary(dv= "MeanRX")
```

```
## [1] 0
```

```
MeanRY= my_Summary(dv= "MeanRY")
```

```
## [1] 0
```

```
#here values are zero because trials without samples for this last phase were dismissed already in the preprocessing phase
```

Same for shift values (bur shift from alert to first number is not yet in the dataframe).

```
MOC$ShiftN1AX<-MOC$MeanN1X-MOC$MeanAX
MOC$ShiftN1AY<-MOC$MeanN1Y-MOC$MeanAY

#shift, alert to first number (x and y)
ShiftN1AX= my_Summary(dv= "ShiftN1AX")
```

```
## [1] -1.738241
```

```
ShiftN1AY= my_Summary(dv= "ShiftN1AY")
```

```
## [1] -1.738241
```

```
#shift, second number to response (x and y)
ShiftNRX= my_Summary(dv= "ShiftNRX")
```

```
## [1] -0.4856851
```

```
ShiftNRY= my_Summary(dv= "ShiftNRY")
```

```
## [1] -0.4856851
```

## Analyses

Another function to ease analyses. It takes one of the tapply objects created before and returns frequentist anova (plus partial eta square) and Bayesian one.

```
my_analysis= function(X){
  
  X= melt(X)
  colnames(X)= c("Subject", "OKS", "Type", "DV")
  X$Subject= as.factor(X$Subject)
  
  EZ= ezANOVA(data=X, dv = .(DV), within = .("OKS","Type"), 
          type = 3, detailed = T, wid= .(Subject))
  print(EZ)
  print(extract.pes(EZ))
  BF= anovaBF(formula = DV ~ OKS*Type + Subject, data = X,
          whichModels = "all", whichRandom = "Subject")
  print(head(BF))
  
}
```

Here we go:

```
my_analysis(MeanN1X)
```

```
## $ANOVA
##        Effect DFn DFd         SSn       SSd         F         p p<.05
## 1 (Intercept)   1  23 1628.714323 59852.156 0.6258827 0.4369523      
## 2         OKS   2  46  416.810789 29377.440 0.3263269 0.7232260      
## 3        Type   1  23    6.827456   822.883 0.1908309 0.6662998      
## 4    OKS:Type   2  46   69.090458  1127.709 1.4091223 0.2547067      
##            ges
## 1 1.754912e-02
## 2 4.550485e-03
## 3 7.487312e-05
## 4 7.571617e-04
## 
## $`Mauchly's Test for Sphericity`
##     Effect         W         p p<.05
## 2      OKS 0.9810046 0.8098068      
## 4 OKS:Type 0.9425507 0.5216172      
## 
## $`Sphericity Corrections`
##     Effect       GGe     p[GG] p[GG]<.05      HFe     p[HF] p[HF]<.05
## 2      OKS 0.9813587 0.7192265           1.072030 0.7232260          
## 4 OKS:Type 0.9456718 0.2550148           1.027831 0.2547067          
## 
##      Effects       pes                 
## [1,] "(Intercept)" "0.0264914000053495"
## [2,] "OKS"         "0.0139896382609797"
## [3,] "Type"        "0.008228721951647" 
## [4,] "OKS:Type"    "0.0577293326702235"
## Bayes factor analysis
## --------------
## [1] Type + Subject            : 0.1747003  ±0.75%
## [2] OKS + Subject             : 0.1326892  ±1.09%
## [3] OKS:Type + Subject        : 0.1293261  ±0.83%
## [4] OKS + Type + Subject      : 0.02343207 ±1.68%
## [5] Type + OKS:Type + Subject : 0.02239703 ±1.15%
## [6] OKS + OKS:Type + Subject  : 0.01664502 ±0.83%
## 
## Against denominator:
##   DV ~ Subject 
## ---
## Bayes factor type: BFlinearModel, JZS
```

```
my_analysis(MeanRX)
```

```
## $ANOVA
##        Effect DFn DFd       SSn       SSd         F         p p<.05
## 1 (Intercept)   1  23 3353.0383 93354.818 0.8260943 0.3728305      
## 2         OKS   2  46 3390.9304 50605.741 1.5411571 0.2249854      
## 3        Type   1  23  170.8845  2381.987 1.6500270 0.2117429      
## 4    OKS:Type   2  46   75.0704  4273.885 0.4039929 0.6699944      
##            ges
## 1 0.0217772935
## 2 0.0220179762
## 3 0.0011332816
## 4 0.0004981728
## 
## $`Mauchly's Test for Sphericity`
##     Effect         W         p p<.05
## 2      OKS 0.9918937 0.9143585      
## 4 OKS:Type 0.8229270 0.1172127      
## 
## $`Sphericity Corrections`
##     Effect       GGe     p[GG] p[GG]<.05       HFe     p[HF] p[HF]<.05
## 2      OKS 0.9919589 0.2251589           1.0852172 0.2249854          
## 4 OKS:Type 0.8495650 0.6371677           0.9102708 0.6510709          
## 
##      Effects       pes                 
## [1,] "(Intercept)" "0.0346718297633345"
## [2,] "OKS"         "0.0627988786543915"
## [3,] "Type"        "0.0669381399798642"
## [4,] "OKS:Type"    "0.0172617094437775"
## Bayes factor analysis
## --------------
## [1] OKS + Subject                   : 1.22742    ±0.76%
## [2] OKS + Type + Subject            : 0.2521955  ±1.12%
## [3] Type + Subject                  : 0.2088778  ±2.49%
## [4] OKS + OKS:Type + Subject        : 0.1530915  ±1.35%
## [5] OKS:Type + Subject              : 0.1228423  ±0.64%
## [6] OKS + Type + OKS:Type + Subject : 0.03326701 ±4.23%
## 
## Against denominator:
##   DV ~ Subject 
## ---
## Bayes factor type: BFlinearModel, JZS
```

```
my_analysis(ShiftN1AX)
```

```
## $ANOVA
##        Effect DFn DFd          SSn       SSd          F         p p<.05
## 1 (Intercept)   1  23 598.37818145 6317.2351 2.17859522 0.1535027      
## 2         OKS   2  46  65.48330209 6766.2621 0.22259202 0.8012984      
## 3        Type   1  23   0.05083782  952.9583 0.00122699 0.9723593      
## 4    OKS:Type   2  46 176.96900446 3600.2956 1.13054249 0.3316632      
##            ges
## 1 3.281458e-02
## 2 3.699155e-03
## 3 2.882485e-06
## 4 9.934422e-03
## 
## $`Mauchly's Test for Sphericity`
##     Effect         W         p p<.05
## 2      OKS 0.9775457 0.7789467      
## 4 OKS:Type 0.8808618 0.2477340      
## 
## $`Sphericity Corrections`
##     Effect       GGe     p[GG] p[GG]<.05       HFe     p[HF] p[HF]<.05
## 2      OKS 0.9780388 0.7965437           1.0679060 0.8012984          
## 4 OKS:Type 0.8935447 0.3274739           0.9638033 0.3303425          
## 
##      Effects       pes                   
## [1,] "(Intercept)" "0.0865256859437682"  
## [2,] "OKS"         "0.00958514961351636" 
## [3,] "Type"        "5.33445266750873e-05"
## [4,] "OKS:Type"    "0.0468511013257122"  
## Bayes factor analysis
## --------------
## [1] OKS:Type + Subject        : 0.2362197  ±0.67%
## [2] Type + Subject            : 0.1775779  ±1.61%
## [3] OKS + Subject             : 0.08994522 ±0.7%
## [4] Type + OKS:Type + Subject : 0.04183251 ±2.21%
## [5] OKS + OKS:Type + Subject  : 0.02191429 ±1.57%
## [6] OKS + Type + Subject      : 0.01640786 ±2.86%
## 
## Against denominator:
##   DV ~ Subject 
## ---
## Bayes factor type: BFlinearModel, JZS
```

```
my_analysis(ShiftNRX)
```

```
## $ANOVA
##        Effect DFn DFd        SSn       SSd         F           p p<.05
## 1 (Intercept)   1  23 5155.69066 30757.999 3.8552860 0.061790975      
## 2         OKS   2  46 2405.51697  8819.087 6.2735396 0.003897712     *
## 3        Type   1  23  152.86868  2857.744 1.2303338 0.278805779      
## 4    OKS:Type   2  46   24.28486  3485.732 0.1602394 0.852413246      
##            ges
## 1 0.1009410520
## 2 0.0497767877
## 3 0.0033179356
## 4 0.0005285654
## 
## $`Mauchly's Test for Sphericity`
##     Effect         W          p p<.05
## 2      OKS 0.8857842 0.26339466      
## 4 OKS:Type 0.7519507 0.04345931     *
## 
## $`Sphericity Corrections`
##     Effect       GGe       p[GG] p[GG]<.05       HFe       p[HF] p[HF]<.05
## 2      OKS 0.8974922 0.005425078         * 0.9686299 0.004312082         *
## 4 OKS:Type 0.8012504 0.805427326           0.8519691 0.818941922          
## 
##      Effects       pes                  
## [1,] "(Intercept)" "0.143557809731838"  
## [2,] "OKS"         "0.214307517491334"  
## [3,] "Type"        "0.0507765949047986" 
## [4,] "OKS:Type"    "0.00691873024570595"
## Bayes factor analysis
## --------------
## [1] OKS + Subject                   : 127.363   ±0.64%
## [2] OKS + Type + Subject            : 40.04969  ±2.95%
## [3] OKS + OKS:Type + Subject        : 16.10382  ±1.21%
## [4] OKS + Type + OKS:Type + Subject : 5.136097  ±3.61%
## [5] Type + Subject                  : 0.2803085 ±1.28%
## [6] OKS:Type + Subject              : 0.1250595 ±0.93%
## 
## Against denominator:
##   DV ~ Subject 
## ---
## Bayes factor type: BFlinearModel, JZS
```

The effect of interest is Operation type. When exploring the horizontal plane, differently from Exp 1, we did not observe a significant shift occurring between the second number presentation and response.

```
my_analysis(MeanN1Y)
```

```
## $ANOVA
##        Effect DFn DFd         SSn        SSd           F         p p<.05
## 1 (Intercept)   1  23    24.46224 304337.507 0.001848709 0.9660753      
## 2         OKS   2  46 20704.19590 310419.338 1.534042655 0.2264908      
## 3        Type   1  23    24.87220   2592.383 0.220669765 0.6429534      
## 4    OKS:Type   2  46   220.83093   4186.929 1.213087487 0.3066100      
##            ges
## 1 3.935616e-05
## 2 3.223746e-02
## 3 4.001570e-05
## 4 3.551724e-04
## 
## $`Mauchly's Test for Sphericity`
##     Effect         W            p p<.05
## 2      OKS 0.4700285 0.0002473807     *
## 4 OKS:Type 0.8891091 0.2744766596      
## 
## $`Sphericity Corrections`
##     Effect       GGe     p[GG] p[GG]<.05       HFe     p[HF] p[HF]<.05
## 2      OKS 0.6536069 0.2304503           0.6770254 0.2304951          
## 4 OKS:Type 0.9001784 0.3041652           0.9719164 0.3059945          
## 
##      Effects       pes                   
## [1,] "(Intercept)" "8.03721885548033e-05"
## [2,] "OKS"         "0.0625271047339847"  
## [3,] "Type"        "0.00950316107682457" 
## [4,] "OKS:Type"    "0.0501004874849497"  
## Bayes factor analysis
## --------------
## [1] OKS + Subject                   : 1.817779   ±3.25%
## [2] OKS + Type + Subject            : 0.2957611  ±1.55%
## [3] OKS + OKS:Type + Subject        : 0.2060486  ±1.11%
## [4] Type + Subject                  : 0.1758626  ±1.44%
## [5] OKS:Type + Subject              : 0.1218758  ±1.63%
## [6] OKS + Type + OKS:Type + Subject : 0.03717083 ±3.25%
## 
## Against denominator:
##   DV ~ Subject 
## ---
## Bayes factor type: BFlinearModel, JZS
```

```
my_analysis(MeanRY)
```

```
## $ANOVA
##        Effect DFn DFd        SSn        SSd         F          p p<.05
## 1 (Intercept)   1  23  2567.2685 455394.286 0.1296617 0.72206867      
## 2         OKS   2  46 10523.9834 452723.308 0.5346569 0.58946661      
## 3        Type   1  23   686.4614   2353.870 6.7075113 0.01637622     *
## 4    OKS:Type   2  46   529.9960   6100.124 1.9983048 0.14716257      
##            ges
## 1 0.0027931237
## 2 0.0113515626
## 3 0.0007483842
## 4 0.0005779032
## 
## $`Mauchly's Test for Sphericity`
##     Effect         W            p p<.05
## 2      OKS 0.4741515 0.0002723259     *
## 4 OKS:Type 0.8006705 0.0866945626      
## 
## $`Sphericity Corrections`
##     Effect       GGe     p[GG] p[GG]<.05       HFe     p[HF] p[HF]<.05
## 2      OKS 0.6553731 0.5172884           0.6790899 0.5232944          
## 4 OKS:Type 0.8337992 0.1558709           0.8911880 0.1528766          
## 
##      Effects       pes                 
## [1,] "(Intercept)" "0.0056058603143528"
## [2,] "OKS"         "0.0227178519919617"
## [3,] "Type"        "0.225785028533568" 
## [4,] "OKS:Type"    "0.0799376131470773"
## Bayes factor analysis
## --------------
## [1] OKS + Subject             : 0.2120621  ±1.02%
## [2] Type + Subject            : 0.1929329  ±1.39%
## [3] OKS:Type + Subject        : 0.1222793  ±0.74%
## [4] OKS + Type + Subject      : 0.0399356  ±1.21%
## [5] OKS + OKS:Type + Subject  : 0.02563314 ±1.21%
## [6] Type + OKS:Type + Subject : 0.02380828 ±2.32%
## 
## Against denominator:
##   DV ~ Subject 
## ---
## Bayes factor type: BFlinearModel, JZS
```

```
my_analysis(ShiftN1AY)
```

```
## $ANOVA
##        Effect DFn DFd        SSn       SSd         F          p p<.05
## 1 (Intercept)   1  23 2716.73424 13903.024 4.4943378 0.04501045     *
## 2         OKS   2  46  560.70417  7765.381 1.6607294 0.20118896      
## 3        Type   1  23   13.62933  2228.738 0.1406511 0.71107070      
## 4    OKS:Type   2  46  255.14852  2508.482 2.3394289 0.10774902      
##            ges
## 1 0.0932868845
## 2 0.0207927502
## 3 0.0005158862
## 4 0.0095701843
## 
## $`Mauchly's Test for Sphericity`
##     Effect         W            p p<.05
## 2      OKS 0.5245480 0.0008272523     *
## 4 OKS:Type 0.9522424 0.5837443205      
## 
## $`Sphericity Corrections`
##     Effect       GGe     p[GG] p[GG]<.05       HFe     p[HF] p[HF]<.05
## 2      OKS 0.6777584 0.2095931           0.7053161 0.2091226          
## 4 OKS:Type 0.9544192 0.1105520           1.0386370 0.1077490          
## 
##      Effects       pes                  
## [1,] "(Intercept)" "0.163464122098749"  
## [2,] "OKS"         "0.0673430753795903" 
## [3,] "Type"        "0.00607809675749195"
## [4,] "OKS:Type"    "0.0923236616287691" 
## Bayes factor analysis
## --------------
## [1] OKS + Subject             : 0.598063   ±2.2%
## [2] OKS:Type + Subject        : 0.2818413  ±0.81%
## [3] Type + Subject            : 0.1851382  ±0.89%
## [4] OKS + OKS:Type + Subject  : 0.1714652  ±1.24%
## [5] OKS + Type + Subject      : 0.1090477  ±1.18%
## [6] Type + OKS:Type + Subject : 0.05171429 ±1.87%
## 
## Against denominator:
##   DV ~ Subject 
## ---
## Bayes factor type: BFlinearModel, JZS
```

```
my_analysis(ShiftNRY)
```

```
## $ANOVA
##        Effect DFn DFd        SSn       SSd          F            p p<.05
## 1 (Intercept)   1  23  1118.3776 27683.099  0.9291837 3.451014e-01      
## 2         OKS   2  46 22698.4068 32316.411 16.1547444 4.849525e-06     *
## 3        Type   1  23  1457.9742  2081.258 16.1120828 5.433369e-04     *
## 4    OKS:Type   2  46   964.8226  5109.866  4.3427599 1.872260e-02     *
##          ges
## 1 0.01637233
## 2 0.25251584
## 3 0.02123822
## 4 0.01415620
## 
## $`Mauchly's Test for Sphericity`
##     Effect         W            p p<.05
## 2      OKS 0.6775496 1.381504e-02     *
## 4 OKS:Type 0.4014895 4.369348e-05     *
## 
## $`Sphericity Corrections`
##     Effect       GGe        p[GG] p[GG]<.05       HFe       p[HF]
## 2      OKS 0.7561720 4.758761e-05         * 0.7980456 0.000032107
## 4 OKS:Type 0.6255824 3.821270e-02         * 0.6443553 0.036870527
##   p[HF]<.05
## 2         *
## 4         *
## 
##      Effects       pes                 
## [1,] "(Intercept)" "0.0388305656332446"
## [2,] "OKS"         "0.412587150455885" 
## [3,] "Type"        "0.411946427539615" 
## [4,] "OKS:Type"    "0.158826685762075" 
## Bayes factor analysis
## --------------
## [1] OKS + Type + Subject            : 2029243053 ±1.71%
## [2] OKS + Subject                   : 1680424448 ±0.73%
## [3] OKS + Type + OKS:Type + Subject : 752715643  ±6.89%
## [4] OKS + OKS:Type + Subject        : 566602061  ±1.15%
## [5] Type + Subject                  : 0.6404481  ±1.14%
## [6] OKS:Type + Subject              : 0.2342181  ±0.67%
## 
## Against denominator:
##   DV ~ Subject 
## ---
## Bayes factor type: BFlinearModel, JZS
```

However, we did replicate the effect for the vertical plane: addition consistently bring updward shifts whereas subtraction brings a downward one. For the shift parameter we also report an interaction OKS by Operation type, that we now explore with post-hocs.

```
#prepare data frame
X= melt(ShiftNRY)
colnames(X)= c("Subject", "OKS", "Type", "DV")
```

For downward OKS there’s not much evidence:

```
t.test(X$DV[X$Type=="Subtraction" & X$OKS=="Down"], 
        X$DV[X$Type=="Addition" & X$OKS=="Down"], paired=T)
```

```
## 
##  Paired t-test
## 
## data:  X$DV[X$Type == "Subtraction" & X$OKS == "Down"] and X$DV[X$Type == "Addition" & X$OKS == "Down"]
## t = -1.8224, df = 23, p-value = 0.08143
## alternative hypothesis: true difference in means is not equal to 0
## 95 percent confidence interval:
##  -8.7971097  0.5568322
## sample estimates:
## mean of the differences 
##               -4.120139
```

```
ttestBF(X$DV[X$Type=="Subtraction" & X$OKS=="Down"], 
        X$DV[X$Type=="Addition" & X$OKS=="Down"], paired=T)
```

When compared against the model 

...

Enter one or more search terms in the box to filter the models in the table. If more than one term is included, matching will be performed with `or`. Special search terms are allowed:

| Code | Function | Example | What example does |
| --- | --- | --- | --- |
| + | Require this term in search results | +shape | Requires all models to include âshapeâ |
| - | Require this term NOT to appear in search results | -shape | Requires all models to exclude âshapeâ |
| # (with number) | Return results with certain number of terms | #2 | Requires all models to have two terms |
| @ (with :, ::, â¦) | Return results containing interactions of certain size | @:: | Requires all models to have a three-way interaction |
| < or > | Limits sizes of Bayes factors | >2 | Returns models whose Bayes factor is greater than 2 |

Click on a row in the Bayes factor table to make that model the denominator. Sort by clicking on the arrows in the column headers.

|  |  |  |
| --- | --- | --- |
| ...the model below... | ...is preferred by... |  |

For static (baseline) OKS, similarly, there’s not much evidence:

```
t.test(X$DV[X$Type=="Subtraction" & X$OKS=="Static"], 
        X$DV[X$Type=="Addition" & X$OKS=="Static"], paired=T)
```

```
## 
##  Paired t-test
## 
## data:  X$DV[X$Type == "Subtraction" & X$OKS == "Static"] and X$DV[X$Type == "Addition" & X$OKS == "Static"]
## t = -1.1347, df = 23, p-value = 0.2682
## alternative hypothesis: true difference in means is not equal to 0
## 95 percent confidence interval:
##  -4.094726  1.193789
## sample estimates:
## mean of the differences 
##               -1.450469
```

```
ttestBF(X$DV[X$Type=="Subtraction" & X$OKS=="Static"], 
        X$DV[X$Type=="Addition" & X$OKS=="Static"], paired=T)
```

When compared against the model 

...

Enter one or more search terms in the box to filter the models in the table. If more than one term is included, matching will be performed with `or`. Special search terms are allowed:

| Code | Function | Example | What example does |
| --- | --- | --- | --- |
| + | Require this term in search results | +shape | Requires all models to include âshapeâ |
| - | Require this term NOT to appear in search results | -shape | Requires all models to exclude âshapeâ |
| # (with number) | Return results with certain number of terms | #2 | Requires all models to have two terms |
| @ (with :, ::, â¦) | Return results containing interactions of certain size | @:: | Requires all models to have a three-way interaction |
| < or > | Limits sizes of Bayes factors | >2 | Returns models whose Bayes factor is greater than 2 |

Click on a row in the Bayes factor table to make that model the denominator. Sort by clicking on the arrows in the column headers.

|  |  |  |
| --- | --- | --- |
| ...the model below... | ...is preferred by... |  |

For upward OKS, instead, the difference seems more evident:

```
t.test(X$DV[X$Type=="Subtraction" & X$OKS=="Up"], 
        X$DV[X$Type=="Addition" & X$OKS=="Up"], paired=T)
```

```
## 
##  Paired t-test
## 
## data:  X$DV[X$Type == "Subtraction" & X$OKS == "Up"] and X$DV[X$Type == "Addition" & X$OKS == "Up"]
## t = -3.077, df = 23, p-value = 0.00533
## alternative hypothesis: true difference in means is not equal to 0
## 95 percent confidence interval:
##  -22.611282  -4.430931
## sample estimates:
## mean of the differences 
##               -13.52111
```

```
ttestBF(X$DV[X$Type=="Subtraction" & X$OKS=="Up"], 
        X$DV[X$Type=="Addition" & X$OKS=="Up"], paired=T)
```

When compared against the model 

...

Enter one or more search terms in the box to filter the models in the table. If more than one term is included, matching will be performed with `or`. Special search terms are allowed:

| Code | Function | Example | What example does |
| --- | --- | --- | --- |
| + | Require this term in search results | +shape | Requires all models to include âshapeâ |
| - | Require this term NOT to appear in search results | -shape | Requires all models to exclude âshapeâ |
| # (with number) | Return results with certain number of terms | #2 | Requires all models to have two terms |
| @ (with :, ::, â¦) | Return results containing interactions of certain size | @:: | Requires all models to have a three-way interaction |
| < or > | Limits sizes of Bayes factors | >2 | Returns models whose Bayes factor is greater than 2 |

Click on a row in the Bayes factor table to make that model the denominator. Sort by clicking on the arrows in the column headers.

|  |  |  |
| --- | --- | --- |
| ...the model below... | ...is preferred by... |  |

Note that all post hocs in this page were not bonferroni corrected, i.e. should be multiplied by 3. Results hold still.

## Plot results

Another function:

```
my_plot= function(X, lab, legend= F, h= T, lims){
  
  X= melt(X)
  colnames(X)= c("Subject", "OKS", "Type", "DV")
  
  DF= summarySEwithin(data = X, measurevar = "DV", 
                      withinvars = c("Type", "OKS"),
                      idvar = "Subject")
  DF$Type= relevel(DF$Type, "Subtraction")
  
  DF$OKS= factor(DF$OKS, levels= c("Down", "Static", "Up"))
  
  pd=position_dodge(1)
  
  p= ggplot(DF, aes(y= DV, x= OKS, fill= Type)) + 
    geom_bar(position= pd, stat= "identity") + ylab(lab) + commonTheme + 
    geom_errorbar(aes(ymin= DV-se, ymax= DV+se), position= pd, width= .4, size= 1.5, colour= "black") +
    ylim(lims) 
  
  #if not
  if(!legend)(p= p + guides(fill= F))
  #if yes
  if(legend) (p= p + theme(legend.position= c(0.5, 0.5),
                      legend.justification= c(0.5, 0.5)) +
              guides(fill= guide_legend(reverse=T, title= "Operation")))
  
    if (h) (p= p  + coord_flip()) 
  
  return(p)
}
```

Horizontal displacement:

```
g_legend<-function(a.gplot){
  tmp <- ggplot_gtable(ggplot_build(a.gplot))
  leg <- which(sapply(tmp$grobs, function(x) x$name) == "guide-box")
  legend <- tmp$grobs[[leg]]
  return(legend)}

grid.arrange(
  a= my_plot(MeanN1X, "Center of Gravity (px) \n First Number", lims= c(-10, 20)),
  b= my_plot(MeanRX, "Center of Gravity (px) \n Response Phase", lims= c(-10, 20)),
  c= my_plot(ShiftN1AX, "Shift (px) \n Alert to First Number", lims= c(-10, 15)),
  d= my_plot(ShiftNRX, "Shift (px) \n Second Number to Response", lims= c(-10, 15)),
  e= g_legend(my_plot(MeanN1X, "I only need the legend here",legend=T, lims= c(-10, 20))),
  layout_matrix = rbind(c(1, 1, 2, 2, 5), c(3, 3, 4, 4, 5)))
```

Vertical Displacement:

```
grid.arrange(
  a= my_plot(MeanN1Y, "Center of Gravity (px) \n First Number", h= F, lims= c(-30, 40)),
  b= my_plot(MeanRY, "Center of Gravity (px) \n Response Phase", h= F, lims= c(-30, 40)),
  c= my_plot(ShiftN1AY, "Shift (px) \n Alert to First Number", h= F, lims= c(-30, 25)),
  d= my_plot(ShiftNRY, "Shift (px) \n Second Number to Response", h= F, lims= c(-30, 25)),
  e= g_legend(my_plot(MeanN1Y, "I only need the legend here",legend=T, h= F, lims= c(-30, 40))),
  layout_matrix = rbind(c(1, 1, 2, 2, 5), c(3, 3, 4, 4, 5)))
```

## Appendix

This is the function to summarise data (`summarySEwithin`):

```
## Summarizes data.
## Gives count, mean, standard deviation, standard error of the mean, and confidence interval (default 95%).
##   data: a data frame.
##   measurevar: the name of a column that contains the variable to be summariezed
##   groupvars: a vector containing names of columns that contain grouping variables
##   na.rm: a boolean that indicates whether to ignore NA's
##   conf.interval: the percent range of the confidence interval (default is 95%)
summarySE <- function(data=NULL, measurevar, groupvars=NULL, na.rm=FALSE,
                      conf.interval=.95, .drop=TRUE) {
  library(plyr)
  
  # New version of length which can handle NA's: if na.rm==T, don't count them
  length2 <- function (x, na.rm=FALSE) {
    if (na.rm) sum(!is.na(x))
    else       length(x)
  }
  
  # This does the summary. For each group's data frame, return a vector with
  # N, mean, and sd
  datac <- ddply(data, groupvars, .drop=.drop,
                 .fun = function(xx, col) {
                   c(N    = length2(xx[[col]], na.rm=na.rm),
                     mean = mean   (xx[[col]], na.rm=na.rm),
                     sd   = sd     (xx[[col]], na.rm=na.rm)
                   )
                 },
                 measurevar
  )
  
  # Rename the "mean" column    
  datac <- plyr::rename(datac, c(mean = measurevar))
  
  datac$se <- datac$sd / sqrt(datac$N)  # Calculate standard error of the mean
  
  # Confidence interval multiplier for standard error
  # Calculate t-statistic for confidence interval: 
  # e.g., if conf.interval is .95, use .975 (above/below), and use df=N-1
  ciMult <- qt(conf.interval/2 + .5, datac$N-1)
  datac$ci <- datac$se * ciMult
  
  return(datac)
}


## Norms the data within specified groups in a data frame; it normalizes each
## subject (identified by idvar) so that they have the same mean, within each group
## specified by betweenvars.
##   data: a data frame.
##   idvar: the name of a column that identifies each subject (or matched subjects)
##   measurevar: the name of a column that contains the variable to be summariezed
##   betweenvars: a vector containing names of columns that are between-subjects variables
##   na.rm: a boolean that indicates whether to ignore NA's
normDataWithin <- function(data=NULL, idvar, measurevar, betweenvars=NULL,
                           na.rm=FALSE, .drop=TRUE) {
  library(plyr)
  
  # Measure var on left, idvar + between vars on right of formula.
  data.subjMean <- ddply(data, c(idvar, betweenvars), .drop=.drop,
                         .fun = function(xx, col, na.rm) {
                           c(subjMean = mean(xx[,col], na.rm=na.rm))
                         },
                         measurevar,
                         na.rm
  )
  
  # Put the subject means with original data
  data <- merge(data, data.subjMean)
  
  # Get the normalized data in a new column
  measureNormedVar <- paste(measurevar, "_norm", sep="")
  data[,measureNormedVar] <- data[,measurevar] - data[,"subjMean"] +
    mean(data[,measurevar], na.rm=na.rm)
  
  # Remove this subject mean column
  data$subjMean <- NULL
  
  return(data)
}


## Retrieved here: http://www.cookbook-r.com/Graphs/Plotting_means_and_error_bars_(ggplot2)/
## Summarizes data, handling within-subjects variables by removing inter-subject variability.
## It will still work if there are no within-S variables.
## Gives count, un-normed mean, normed mean (with same between-group mean),
##   standard deviation, standard error of the mean, and confidence interval.
## If there are within-subject variables, calculate adjusted values using method from Morey (2008).
##   data: a data frame.
##   measurevar: the name of a column that contains the variable to be summariezed
##   betweenvars: a vector containing names of columns that are between-subjects variables
##   withinvars: a vector containing names of columns that are within-subjects variables
##   idvar: the name of a column that identifies each subject (or matched subjects)
##   na.rm: a boolean that indicates whether to ignore NA's
##   conf.interval: the percent range of the confidence interval (default is 95%)
summarySEwithin <- function(data=NULL, measurevar, betweenvars=NULL, withinvars=NULL,
                            idvar=NULL, na.rm=FALSE, conf.interval=.95, .drop=TRUE) {
  
  # Ensure that the betweenvars and withinvars are factors
  factorvars <- vapply(data[, c(betweenvars, withinvars), drop=FALSE],
                       FUN=is.factor, FUN.VALUE=logical(1))
  
  if (!all(factorvars)) {
    nonfactorvars <- names(factorvars)[!factorvars]
    message("Automatically converting the following non-factors to factors: ",
            paste(nonfactorvars, collapse = ", "))
    data[nonfactorvars] <- lapply(data[nonfactorvars], factor)
  }
  
  # Get the means from the un-normed data
  datac <- summarySE(data, measurevar, groupvars=c(betweenvars, withinvars),
                     na.rm=na.rm, conf.interval=conf.interval, .drop=.drop)
  
  # Drop all the unused columns (these will be calculated with normed data)
  datac$sd <- NULL
  datac$se <- NULL
  datac$ci <- NULL
  
  # Norm each subject's data
  ndata <- normDataWithin(data, idvar, measurevar, betweenvars, na.rm, .drop=.drop)
  
  # This is the name of the new column
  measurevar_n <- paste(measurevar, "_norm", sep="")
  
  # Collapse the normed data - now we can treat between and within vars the same
  ndatac <- summarySE(ndata, measurevar_n, groupvars=c(betweenvars, withinvars),
                      na.rm=na.rm, conf.interval=conf.interval, .drop=.drop)
  
  # Apply correction from Morey (2008) to the standard error and confidence interval
  #  Get the product of the number of conditions of within-S variables
  nWithinGroups    <- prod(vapply(ndatac[,withinvars, drop=FALSE], FUN=nlevels,
                                  FUN.VALUE=numeric(1)))
  correctionFactor <- sqrt( nWithinGroups / (nWithinGroups-1) )
  
  # Apply the correction factor
  ndatac$sd <- ndatac$sd * correctionFactor
  ndatac$se <- ndatac$se * correctionFactor
  ndatac$ci <- ndatac$ci * correctionFactor
  
  # Combine the un-normed means with the normed results
  merge(datac, ndatac)
}
```
